# Clinically Relevant Characterization of Lung Adenocarcinoma Subtypes Based on Cellular Pathways: An International Validation Study

**DOI:** 10.1371/journal.pone.0011712

**Published:** 2010-07-22

**Authors:** Christopher M. Bryant, Daniel L. Albertus, Sinae Kim, Guoan Chen, Christian Brambilla, Mickael Guedj, Chinatsu Arima, William D. Travis, Yasushi Yatabe, Takashi Takahashi, Elisabeth Brambilla, David G. Beer

**Affiliations:** 1 Thoracic Surgery, Department of Surgery, University of Michigan Medical School, Ann Arbor, Michigan, United States of America; 2 Comprehensive Cancer Center, University of Michigan Medical School, Ann Arbor, Michigan, United States of America; 3 Department of Biostatistics, University of Michigan Medical School, Ann Arbor, Michigan, United States of America; 4 Ligue Nationale Contre le Cancer, Programme Cartes d'Identité des Tumeurs, Université Joseph Fourier, Grenoble, France; 5 Department of Pathology, Memorial Sloan-Kettering Cancer Center, New York, New York, United States of America; 6 Pathology and Molecular Diagnostics, Aichi Cancer Center Hospital, Nagoya, Japan; 7 Division of Molecular Carcinogenesis, Center for Neurological Diseases and Cancer, Nagoya University Graduate School of Medicine, Nagoya, Japan; 8 Département d'Anatomie et Cytologie Pathologiques, Université Joseph Fourier, Grenoble, France; Washington University, United States of America

## Abstract

Lung adenocarcinoma (AD) represents a predominant type of lung cancer demonstrating significant morphologic and molecular heterogeneity. We sought to understand this heterogeneity by utilizing gene expression analyses of 432 AD samples and examining associations between 27 known cancer-related pathways and the AD subtype, clinical characteristics and patient survival. Unsupervised clustering of AD and gene expression enrichment analysis reveals that cell proliferation is the most important pathway separating tumors into subgroups. Further, AD with increased cell proliferation demonstrate significantly poorer outcome and an increased solid AD subtype component. Additionally, we find that tumors with any solid component have decreased survival as compared to tumors without a solid component. These results lead to the potential to use a relatively simple pathological examination of a tumor in order to determine its aggressiveness and the patient's prognosis. Additional results suggest the ability to use a similar approach to determine a patient's sensitivity to targeted treatment. We then demonstrated the consistency of these findings using two independent AD cohorts from Asia (N = 87) and Europe (N = 89) using the identical analytic procedures.

## Introduction

Lung cancer is the second most common cancer in the United States and the most common cause of cancer-related death [1]. The overall five-year survival rate is only 15% for lung cancer patients and more than half of patients present with metastatic disease at time of first diagnosis [2]. Patients with early stage disease have a significantly better prognosis, therefore detecting and diagnosing lung cancer early is extremely important [2]. Unfortunately, one third of patients with the earliest stage IA lung cancer will succumb to their disease. Thus identifying high-risk individuals and characterizing the cellular pathways underlying aggressive lung cancer behavior may lead to better therapeutic approaches to increase patient survival.

Non-small cell lung cancer (NSCLC) accounts for the majority of lung cancer and adenocarcinomas (AD) and squamous cell carcinomas (SCC) represent the most common types of NSCLC. AD are increasing in incidence and we and others have recently comprehensively examined large numbers of AD by gene expression profiling [3], DNA copy number variation [4] and the mutational status of key cancer-related genes [5]. Clinical covariates such as age, gender and tumor stage offer prognostic information and these factors were found to improve the prognostic performance of gene-expression based predictors for AD survival [3]. The molecular as well as the pathological heterogeneity of lung adenocarcinomas (AD) has been described [6], however the exact relationships between the specific AD subtypes to each other, or to clinical and molecular variables has not been adequately addressed. The foundation for classification of AD is pathology with several subtypes being recognized [7]. These include carcinoma *in situ* or CIS (formerly called bronchioalveolar carcinoma or BAC), which retain the normal alveolar architecture but with neoplastic cell replacement and a lepidic growth pattern. The acinar AD subtype demonstrate the characteristic glandular pattern, the papillary AD subtype shows finger-like tumor cell projections with a sparce stromal core and the solid AD subtype demonstrate a more compressed structure without features associated with the other main subtypes.

**Figure 1:**
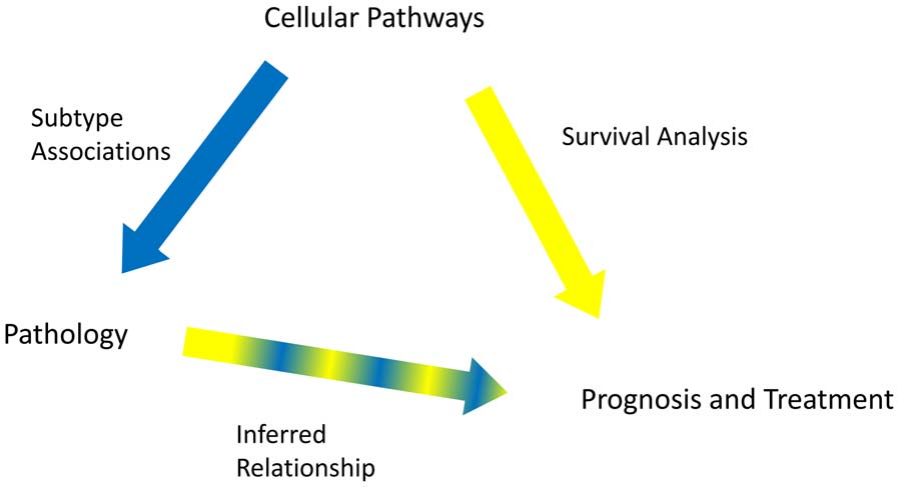
Overall Study Design. We developed cellular pathway expression summaries and tested the relationship of each to pathological subtypes of lung adenocarcinoma (AD). We also tested each pathway's association with survival. Because the cellular pathways are driving the pathological differences, the relationship between pathology and prognosis is secondary to the relationship between the cellular pathway and prognosis (indicated by a thinner line with both component colors). We also directly tested the relationship between pathology and prognosis to examine the need for molecular information.

The current system of AD classification does not adequately capture the heterogeneity of these tumors and classification using clinical, pathological and gene-expression based approaches tend to be treated as separate modalities. Investigating the associations and interactions between them could yield powerful new insights into more effective and clinically-relevant ways to classify AD but has been hampered by previous gene expression studies examining only relatively small numbers of tumors. We have now combined our recent analysis of over 400 AD samples using gene expression profiling [3] with complete clinical information and a newly performed uniform pathological review of these tumors. We hypothesized that the heterogeneity of AD may reflect differences in the expression levels of cancer-related pathways. We have utilized sets of genes representing 27 separate cellular processes (referred to as *pathways*) to investigate relationships between tumors and separate AD subtypes. The relationships observed between gene expression, clinical information including survival, and AD pathology we suggest, have potential translational and clinical implications. Our overall study design is summarized in Figure 1 and was independently validated in two additional independent datasets.

## Methods

### Gene Expression Data

Affymetrix 133A data was obtained for the 443 lung AD described by Shedden et al [3] with the entire set of arrays quantile normalized for that study. A subset of 11 tumors were removed from further analysis based on neuroendocrine features following pathological review of all tumors by one of the study pathologists (W. Travis). The expression of CHGA, SCG2, CHGB (chromogranin), NCAM1 (CD56) and SYP (synaptophysin) in the microarray data were used to support this decision as they are highly expressed in large cell neuroendocrine carcinomas (LCNEC).

### Statistical Methods

Prior to any data analysis, individual tumor gene expression levels were log2-transformed to remove skewness and mean-centered within each of the four sites [3] to remove potential site effects not removed by the quantile normalization. The four sets of tumor data were combined and the dataset reduced by removing approximately 25% of the least-varying genes across all samples. The final dataset for analysis included 432 tumors with 16,660 (of the original 22,214) genes. All final regression models were chosen using stepwise model selection, based on Akaike's Information Criterion (AIC, lower means better fit). All source code is included in the Supplemental Data as R Code S1.

### Clustering

Tumors were hierarchically clustered based on all 16,660 genes in the final data set using Cluster and Treeview Eisen software [8]. Genes and arrays were median-centered within the Cluster program, with the average linkage method of clustering used. “Clusters” were identified using the zero correlation level as a separator of distinct groups, such that the level of correlation between clusters is negative but the nodes within a single cluster have positive correlation. Pearson correlations between each gene and an indicator variable for each cluster were computed to find those genes with significantly higher or lower expression within one group as compared to all other tumors, and the Bonferonni method was used to conservatively adjust the p-values for multiple comparisons. This analysis resulted in two lists of genes for each cluster, those over-expressed and those under-expressed within the cluster (as compared to the other clusters). Descriptive statistics by cluster were produced for each of the clinical variables (see Table 1). Gene enrichment analysis was used to test the gene sets for enrichment of the pathways described previously (see Table 2), using Fisher's Exact Tests to determine the pathways described in each list. Kaplan-Meier survival curves were plotted for each cluster and the associated log-rank test was computed to determine differences in outcome by cluster.

**Table 1:** Cluster Descriptives.

Variable	Overall (%)	Cluster 1 (%)	Cluster 2 (%)	Cluster 3 (%)	p-value
**Number of Tumors**	432 (100)	137 (32)	130 (30)	165 (38)	N/A
**Stage 1**	268 (62)	97 (36)	85 (32)	89 (33)	0.0098
**Stage 2**	93 (22)	23 (25)	25 (27)	45 (48)	0.064
**Stage 3**	69 (16)	16 (23)	23 (33)	30 (43)	0.26
**Unknown Stage**	2	1	0	1	N/A
**High Grade**	160 (37)	13 (8)	47 (29)	100 (63)	<0.0001
**Low/Intermediate Grade**	265 (61)	123 (46)	82 (31)	60 (23)	<0.0001
**Unknown Grade**	7 (2)	1	1	5	N/A
**Male**	217 (50)	70 (32)	62 (29)	88 (41)	0.59
**Female**	215 (50)	67 (31)	68 (32)	77 (36)	0.59
**Age at Diagnosis**	64.5	66.6	63.1	63.8	0.0095
**Percent CIS (Mean)**	6.5	12.2	5.4	1.8	<0.0001
**Percent Papillary (Mean)**	30.7	45.3	27.3	19.3	<0.0001
**Percent Acinar (Mean)**	34.3	32.4	39.7	31.4	0.042
**Percent Solid (Mean)**	25.9	9.0	25.9	42.4	<0.0001

Chi-squared tests showed significant differences between the clusters for the presence of stage 1 and stage 2 tumors as well as grade. There were no significant differences for stage 3 and sex between the clusters. Abbreviations: CIS, carcinoma *in situ*.

**Table 2:** Select Gene Enrichment p-values.

Pathway	Cluster 1 (+)	Cluster 2 (+)	Cluster 3 (+)	Cluster 1 (−)	Cluster 2 (−)	Cluster 3 (−)
Complement	0.015	NS	NS	NS	NS	<0.0001
Chemokine	NS	<0.0001	NS	<0.0001	NS	NS
T-cell	NS	<0.0001	NS	0.0019	NS	NS
Antigen	NS	0.00086	NS	NS	NS	0.028
NFKB	NS	0.0015	NS	NS	NS	NS
B-cell	NS	0.0087	NS	NS	NS	NS
ESC	NS	NS	<0.0001	<0.0001	NS	NS
Cell Cycle (+)	NS	NS	<0.0001	<0.0001	NS	NS

The complement pathway had a significant number of probe sets that were also in the Cluster 1 positively (over-expressed) correlated (p<0.05 after Bonferroni correction for multiple tests) probe set list. The complement pathway also had a significant number of probe sets that were also in the Cluster 3 negatively correlated probe set list. The chemokine, T-cell, antigen, NF-κB and B-cell pathways were significantly enriched in the positively correlated Cluster 2 probe set list. The chemokine and T-cell pathways were also significantly enriched in the Cluster 1 negatively correlated probe set list and the antigen pathway was significantly enriched in the Cluster 3 negatively correlated probe set list. The embryonic stem cell (ESC) and cell cycle stimulatory (CC+) pathways were significantly enriched in the Cluster 3 positively correlated probe set list as well as the Cluster 1 negatively correlated probe set list.

### Pathways

Pathway gene lists were developed based on primary literature sources, KEGG pathways and by referencing OMIM (http://www.ncbi.nlm.nih.gov/sites/entrez?db=OMIM). The pathways include only genes that are highly specific to the pathway and act to either stimulate or suppress the pathway (as indicated). The embryonic stem cell pathway (ESC) was based on genes associated as defined in the Ben-Porath et al. publication [9]. All pathway lists and references are provided in the Supplemental Data as Gene Lists S1 and References S1. Pathway expression data were formed as the arithmetic mean of all genes in the final dataset within the compiled lists, leaving one value for each tumor for each pathway.

### Cluster Membership

Prior to performing any further analyses, pathways that were highly predicted by other pathway(s) were removed from the analyses to protect against multicolinearity (redundancy among predictive variables). Each pathway was used as the outcome variable in a linear regression with all other pathways as predictors, and the pathway with the highest R^2^ was removed. This process was continued until no pathway was predicted with an R^2^ at least equal to 0.7. In order to determine general gene-based tumor profiles using pathways of interest and the clinical data at hand, logistic regression was used with cluster membership as the outcome. The pathways were used as covariates along with age, gender, stage, and tumor grade.

### Survival Analyses

A Cox proportional-hazards model was fit to assess differences in 5-year survival using the same covariates as described above. The proportional-hazards assumption was tested for the final model to examine the model's appropriateness. Additionally, a Cox proportional hazards model was fit to the AD subtypes. Kaplan-Meier curves and associated log-rank tests were computed for selected descriptive statistics as well as to compare over- and under-expression (from the mean) of selected pathways.

### Subtype Associations

The percentage of each AD subtype present in the primary tumor in 5% increments (as determined by W. Travis) was recorded for each tumor. Linear regression models were fit to the estimated percentage using the final set of pathways as covariates. Pathological review in 5% increments was not available for one AD cohort from [3], leaving the sample size of n = 323 for the analyses involving subtype. Logistic regression was used to model the odds of the presence of each particular subtype (separate model for each subtype) with the same covariates.

### Validation

Two independent cohorts of lung AD with gene expression data and new complete and comparable pathological review were used to assess the findings with one cohort from Nagoya, Japan (Takahashi et al [10]) containing 87 lung AD, and another 89 lung AD from Grenoble, France (Brambilla et al [11]). The same statistical and pathological analyses were performed separately on each of these groups of tumors for a qualitative validation of the results from the 432 AD analyzed in this study. Statisticians from each of these groups followed the methods described above as closely as possible.

## Results

### Overall

As graphically described in Figure 1, we developed cellular pathway expression summaries then tested each pathway to determine its relationship to pathologic subtypes of lung adenocarcinoma (AD). We also tested each pathway's association with survival. We hypothesize that the cellular pathways are likely driving the pathologic differences, therefore the relationship between pathology and prognosis is secondary to the relationship between the cellular pathway and prognosis. However, we also directly tested the relationship between pathology and prognosis to examine the need for molecular information.

### Clustering

**Figure 2:**
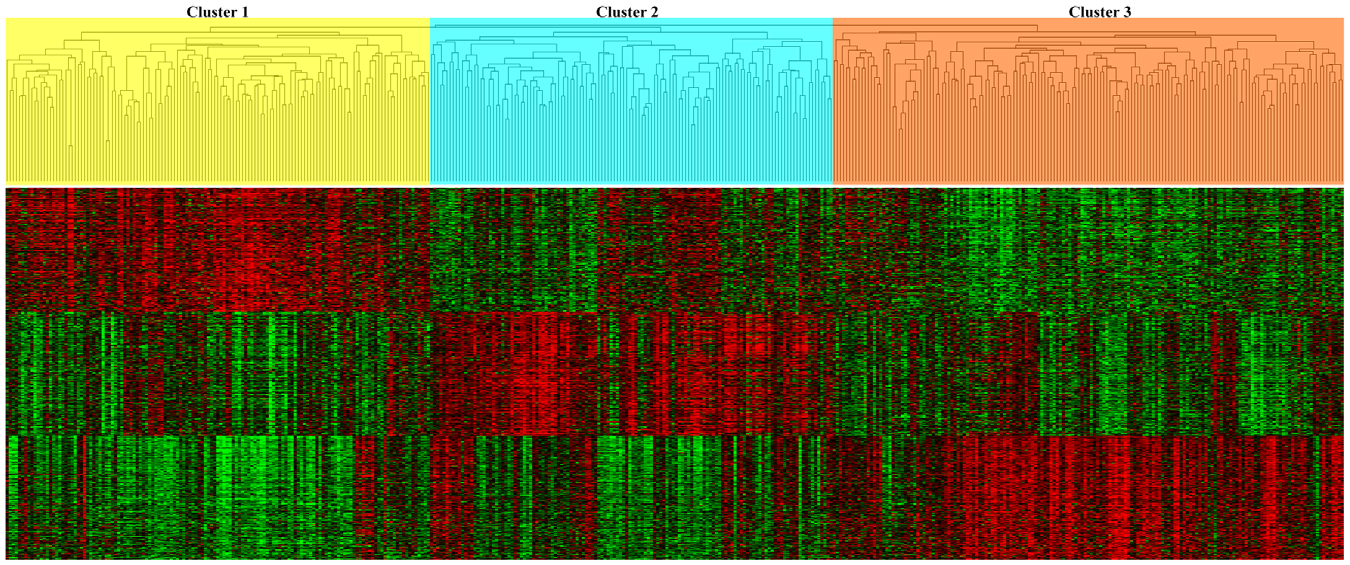
Hierarchical clustering yields three distinct groups. Hierarchical clustering of all 432 lung adenocarcinomas (AD) which shows three main groups of tumors denoted by yellow, blue and orange in the dendrogram. Below is a heat map that represents the 200 genes that are most highly correlated to the left most cluster followed by the middle cluster and the right most cluster. Red indicates relative over-expression (compared to the median) while green indicates relative under-expression.

**Figure 3:**
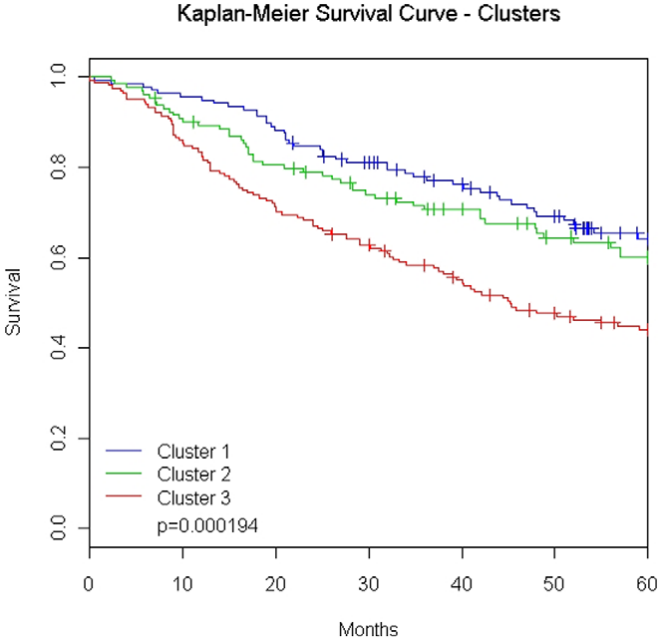
Clusters show survival differences. Kaplan-Meier survival curves for 432 lung adenocarcinomas (AD) showing a significant difference between clusters (log-rank test: p = 0.000194). Abbreviations: Cluster 1, left most cluster in Figure 2; Cluster 2, middle cluster is Figure 2; Cluster 3, right most cluster in Figure 2.

Hierarchical clustering of all 432 samples with the 16,660 most variably expressed genes yielded three distinct lung AD groups. A dendrogram of the three clusters including a heat-map of the 200 most significantly over-expressed genes in each cluster is shown in Figure 2. The patients within each cluster demonstrated a significant (p-value <0.001) difference in overall survival (Figure 3). Cluster 3 includes patients with worse overall survival and more poorly-differentiated whereas those in cluster 1 were more well-differentiated and had a more favorable outcome. Complete clinical and pathological descriptive statistics for each cluster are provided in Table 1.

Following selected gene enrichment and determination of the genes most associated with each cluster, the correlation of each cluster to the 27 separate pathways was assessed. Those pathways significantly associated with cluster membership are shown in Table 2. More detailed results are provided in Supplemental Data as Table S10. Cluster 3 demonstrating the poorest survival outcome showed significant enrichment of the embryonic stem cell (ESC) and cell cycle stimulatory (CC+) pathways. The high cell cycle expression is consistent with the poorer outcome and more aggressive AD in this group. Cluster 2 showed significant enrichment of several immune-related pathways (complement, T-cell, B-cell, antigen, NF-kB) potentially reflecting either increased immune cell presence, or their activity in these tumors. These tumors may be mounting a more successful immune response, given their improved outcome relative to Cluster 3. Cluster 1 also showed enrichment for several immune response pathways but most interestingly showed strong enrichment for the under-expression of the CC+ and ESC pathways indicating that cell proliferation is lowest in tumors within this cluster.

### Cluster Membership

Modeling the odds of cluster membership (performed separately for each cluster), the findings are consistent with the pathway enrichment results. The final logistic regression models are shown in Table 3. Tumors with higher cell cycle (CC+) pathway levels were far more likely to be in Cluster 3 than in the other two clusters. Tumors with greater complement pathway values were more likely to be in Cluster 2 than Cluster 1 or Cluster 3. Increased immune response pathways corresponded largely to a greater likelihood of tumor membership in Cluster 2.

**Table 3:** Clusters and Pathways.

	Cluster 1		Cluster 2		Cluster 3	
Pathway Name	Coefficient	P-value	Coefficient	P-value	Coefficient	P-value
**Intercept**	−2.95	0.0020	0.34	0.71	−0.74	0.0010
**Cell Cycle (+)**	−0.76	<0.0001	−0.70	<0.0001	1.69	<0.0001
**ESC**	NA	NA	NA	NA	NA	NA
**B-cell**	−0.57	0.0023	0.52	0.0049	NA	NA
**T-cell**	NA	NA	NA	NA	NA	NA
**Antigen**	NA	NA	0.87	0.0007	−0.47	0.013
**AKT/PI3K**	NA	NA	NA	NA	−0.61	0.0014
**IGF-1**	-0.88	<0.0001	0.99	<0.0001	NA	NA
**Chemokine**	NA	NA	NA	NA	NA	NA
**NF-κB**	NA	NA	NA	NA	NA	NA
**Notch**	NA	NA	0.34	0.048	NA	NA
**JAK/STAT**	NA	NA	0.48	0.016	NA	NA
**Complement**	0.56	0.0023	NA	NA	−0.81	<0.0001
**mTOR**	NA	NA	NA	NA	NA	NA
**Cell Cycle (−)**	NA	NA	NA	NA	NA	NA
**Angiogenesis**	−0.47	0.011	NA	NA	0.39	0.035
**IL-stimulatory**	NA	NA	−0.28	0.11	0.40	0.029
**IL-suppressive**	−0.37	0.030	NA	NA	NA	NA
**Interferon**	0.36	0.010	−0.27	0.056	0.30	0.073
**EGFR**	NA	NA	−0.26	0.11	NA	NA
**PDGF**	NA	NA	0.56	0.0007	−0.41	0.016
**Hypoxia**	0.35	0.033	NA	NA	−0.39	0.029
**PTEN**	NA	NA	NA	NA	NA	NA
**Pro-apoptosis**	NA	NA	NA	NA	NA	NA
**Anti-apoptosis**	0.40	0.041	−0.46	0.030	NA	NA
**TGF-β**	NA	NA	NA	NA	−0.36	0.045
**Hedgehog**	NA	NA	NA	NA	NA	NA
**Wnt**	0.38	0.022	NA	NA	−0.43	0.015
**Male**	NA	NA	NA	NA	−0.44	0.16
**Age**	0.032	0.023	−0.030	0.026	NA	NA
**Grade**	−1.49	<0.0001	0.50	0.13	NA	NA
**Stage**	NA	NA	NA	NA	NA	NA

Tumors with lower levels of the cell cycle stimulatory (CC+) were more likely to be in Cluster 1 or 2, other pathways held constant. Tumors with higher levels of the IGF-1 pathway were more likely to be in Cluster 2 and less likely to be in Cluster 1. Samples with increased antigen pathway expression were more likely to be in Cluster 2 and less likely to be in Cluster 3. Tumors with increased complement pathway expression were more likely to be in Cluster 1 and less likely to be in Cluster 3. NA indicates that the variable was excluded during model selection and thus deemed unimportant.

### Survival Analyses

**Figure 4:**
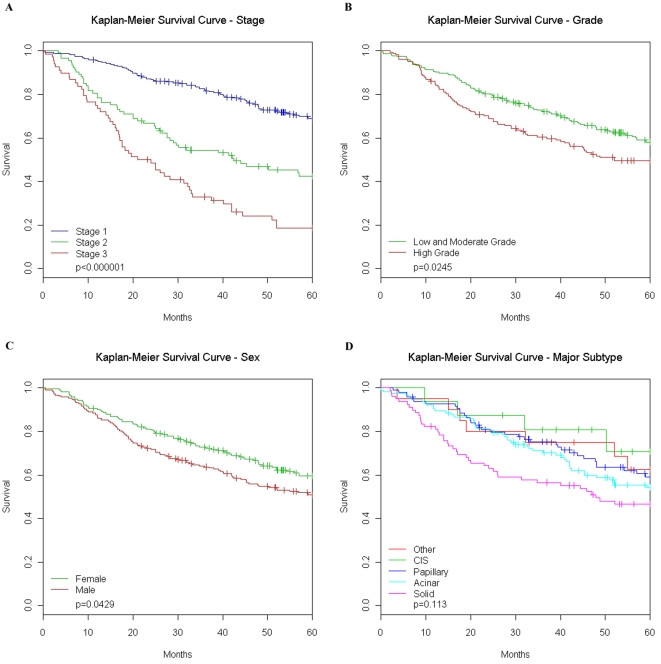
Stage, grade, sex and pathology effects on survival. Log-rank tests of differences between Kaplan Meier survival curves verify that the dataset is consistent with previous results. Higher stage patients do significantly poorer as compared to lower stages (**A**) and high grade patients (**B**) (poor differentiation) have increased hazards compared to low or intermediate grade patients. Gender is a marginally significant prognostic indicator; males have poorer survival (**C**). Additionally we examined pathology (**D**) defined by major lung adenocarcinoma (AD) subtype (plurality of tumor cross section). There was no significant overall difference between the four main subtypes as well as tumors that did not fall into one of these categories. Abbreviations: CIS, carcinoma *in situ*.

**Figure 5:**
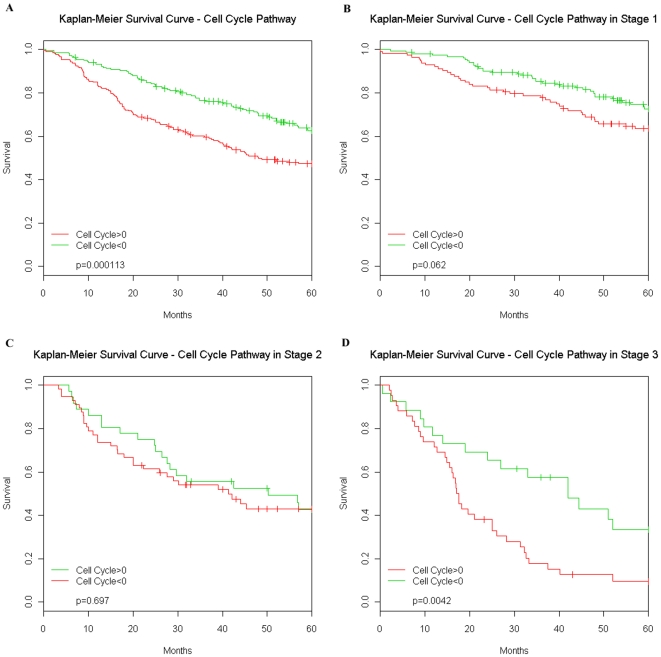
Stage specific survival differences of cell cycle pathway. (**A**) Kaplan-Meier survival analysis of the cell cycle stimulatory (CC+) pathway shows that patients with relative over-expression of CC+ do significantly worse (p = 0.000113). This trend is consistent inside each stage however is only marginally significant (p = 0.062) in stage 1 patients (**B**), not significant in stage 2 patients (**C**) and highly significant (p = 0.0042) in stage 3 patients (**D**). Abbreviations: w/, with; w/o, without.

The multivariate Cox proportional-hazards model confirmed results from previous studies that stage and age are strong prognostic indicators in AD (final model shown in Table 4 and Kaplan-Meier survival curves shown in Figure 4). However, several pathways gave additional prognostic information across all stages of tumors and some had significant interactions with stage. Increased expression of the CC+ pathway yielded a significantly increased hazard rate while increased expression of EGFR and B-cell pathways was associated with a decreased hazard rate, holding other covariates constant. Higher values of the Notch and the immunosuppressive interleukin (IL-) pathways were associated with poorer outcomes in stage 3 and stage 2 patients respectively. Kaplan-Meier survival curves based on either the over- or under- expression (relative to the mean of all tumors) of the CC+ pathway are shown in Figure 5. Additional information is given in the Supplemental Data as Table S15.

**Table 4:** Survival and Pathways.

Pathway Name or Other Variable	Coefficient	P-value
**Stage 2**	1.00	<0.0001
**Stage 3**	1.56	<0.0001
**Cell Cycle (+)**	0.37	<0.0001
**Notch**	0.015	0.91
**Hedgehog**	0.14	0.09
**B-cell**	−0.26	0.037
**Hypoxia**	−0.20	0.11
**EGFR**	−0.21	0.014
**IL-suppressive**	0.19	0.20
**Cell Cycle (−)**	−0.14	0.12
**Age**	0.035	<0.0001
**Stage 2 : Notch**	−0.056	0.78
**Stage 3 : Notch**	0.39	0.043
**Stage 2 : B-cell**	−0.39	0.11
**Stage 3 : B-cell**	0.21	0.36
**Stage 2 : Hypoxia**	0.26	0.17
**Stage 3 : Hypoxia**	0.42	0.063
**Stage 2 : IL-suppressive**	0.70	0.0045
**Stage 3 : IL-suppressive**	0.11	0.61

The cell cycle stimulatory (CC+) pathway gives additional prognostic information beyond standard clinical covariates such as stage and age, where patients with greater expression levels of the CC+ pathway have an increased hazard compared to those with relative underexpression. The B-cell and EGFR pathways also give additional information where patients with relative overexpression of either of these pathways to better although the relationship with the B-cell pathway only exists in stage 1 patients. In addition, stage 3 patients with relative overexpression of the Notch or the response to hypoxia pathway do worse while stage 2 patients with relative overexpression of pathway representing activity of immunosuppressive interleukins did poorer. Variables not listed were dropped during model selection.

### Subtype Associations

Final reduced models showing pathways correlated with the percent of each subtype are shown in Table 5. Results for each AD subtype using presence of subtype can be found in Supplemental Data as Table S11, Table S12, Table S13 and Table S14. Tumors with higher expression of the CC+ pathway, holding other covariates constant, tended to contain less of the carcinoma *in situ* (CIS) component. Greater values of the complement and PGDF pathways, as well as lower values of the EGFR pathway, were associated with a greater acinar component after adjusting for other covariates. Tumors with lower levels of CC+ pathway expression and higher levels of Hedgehog, Notch, and the EGFR pathway expression tended to have more of a papillary component. Finally, tumors with greater CC+, anti-apoptosis and angiogenic pathways as well as lower Notch and complement pathways tended to have a greater proportion of the solid component.

**Table 5:** Pathology and Pathways.

	CIS-ness (%)		Acinar-ness (%)		Papillary-ness (%)		Solid-ness (%)	
Pathway Name	Coefficient	P-value	Coefficient	P-value	Coefficient	P-value	Coefficient	P-value
**Intercept**	6.32	<0.0001	34.72	<0.0001	30.34	<0.0001	26.92	<0.0001
**Cell Cycle (+)**	−5.65	<0.0001	4.38	0.031	−8.89	<0.0001	7.41	0.0011
**ESC**	NA	NA	NA	NA	NA	NA	NA	NA
**B-cell**	NA	NA	−4.04	0.034	NA	NA	3.53	0.080
**T-cell**	NA	NA	NA	NA	NA	NA	NA	NA
**Antigen**	NA	NA	NA	NA	NA	NA	NA	NA
**AKT/PI3K**	1.58	0.072	NA	NA	NA	NA	NA	NA
**IGF-1**	NA	NA	6.81	0.0001	−3.95	0.028	NA	NA
**Chemokine**	NA	NA	NA	NA	NA	NA	NA	NA
**NF-κB**	NA	NA	NA	NA	NA	NA	NA	NA
**Notch**	NA	NA	NA	NA	5.28	0.0068	−6.85	0.0007
**JAK/STAT**	NA	NA	NA	NA	NA	NA	3.081	0.14
**Complement**	NA	NA	5.61	0.0014	NA	NA	−6.53	0.0015
**mTOR**	NA	NA	−4.99	0.015	NA	NA	3.65	0.087
**Cell Cycle (−)**	NA	NA	2.7	0.077	NA	NA	NA	NA
**Angiogenesis**	NA	NA	−3.39	0.042	−5.34	0.0052	8.21	<0.0001
**IL-stimulatory**	−2.32	0.012	NA	NA	NA	NA	NA	NA
**IL-suppressive**	NA	NA	NA	NA	NA	NA	NA	NA
**Interferon**	NA	NA	NA	NA	NA	NA	NA	NA
**EGFR**	NA	NA	−4.29	0.0035	5.58	0.0003	NA	NA
**PDGF**	NA	NA	3.68	0.025	NA	NA	NA	NA
**Hypoxia**	NA	NA	NA	NA	NA	NA	NA	NA
**PTEN**	NA	NA	NA	NA	NA	NA	NA	NA
**Pro-apoptosis**	NA	NA	NA	NA	NA	NA	−3.71	0.16
**Anti-apoptosis**	1.71	0.079	−3.40	0.075	−3.68	0.056	6.21	0.0079
**TGF-β**	NA	NA	NA	NA	NA	NA	NA	NA
**Hedgehog**	NA	NA	NA	NA	3.77	0.028	−4.45	0.010
**Wnt**	NA	NA	2.36	0.13	−3.10	0.087	−4.10	0.025

Carcinoma *in situ* (CIS) tumors are best described by relative underexpression of the cell cycle stimulatory (CC+) pathway. This relationship was consistent when taking into account only the presence or absence (+/−) of a CIS component of the tumor as well as the percentage (%) of the tumor that was CIS. Acinar tumors tended to have relative overexpression of the complement and PDGF pathways and relative underexpression of the angiogenesis pathway. Relative underexpression of the CC+ pathway was a strong indicator of papillary tumors as were relative overexpression of the EGFR and hedgehog pathways. Solid tumors were best defined by relative overexpression of the CC+, JAK/STAT and angiogenesis pathways and by relative underexpression of the Notch pathway. NA means the variable was excluded during model selection.

### Subtype Survival Analyses

**Figure 6:**
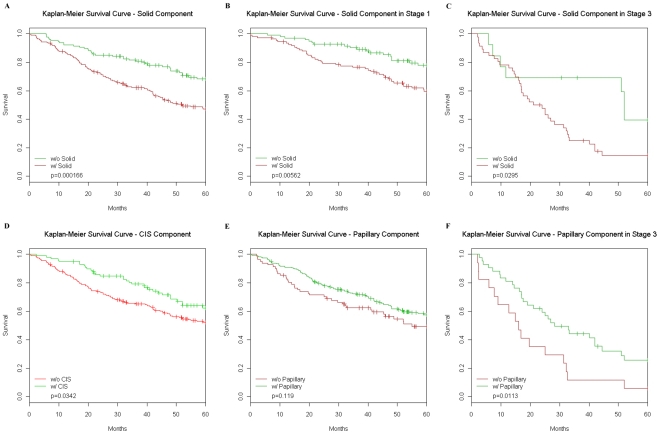
Survival differences for different pathologies. (**A**) Kaplan-Meier survival analysis of solid lung adenocarcinoma (AD) shows that patients with any solid component do significant worse (p = 0.000166) than those with no solid component. This trend is highly significant (p = 0.00562) in stage 1 patients (**B**) and marginally significant (p = 0.0295) in stage 3 patients (**C**). Those patients with any carcinoma *in situ* (CIS) component (**D**) did significantly better (p = 0.0342) than those without any CIS component. Comparing patients with papillary component (**E**) to those without any papillary component showed no significant difference but in stage 3 patients (**F**) those with some papillary component did significantly better than those without any papillary component. Abbreviations: w/, with; w/o, without.

Because the pathological subtypes are so highly associated with certain pathways they dropped out of the multivariate survival analysis described in Table 4 (see Methods) during model selection. Therefore we found that information on the pathological subtype gives little additional prognostic value, when using either the subtype presence indicator or the continuous variable. However, a multivariate Cox model including only the pathological subtypes suggested that patients with some solid component had almost twice the hazard as those with no solid component, with an associated p-value of 0.002 as shown in Table 6. We also observed that the presence of a solid component was associated with a poorer survival in all patients and within either stage 1 or stage 3 patients (Figure 6 A–C). The presence of BAC component was favorable for survival as was presence of a Papillary component in stage 3 patients of this cohort (Figure 6 D–F). Using the percent subtype variable, we found no significant survival differences as shown in Supplemental Data as Table S16.

**Table 6:** Pathology and Survival.

Pathological Subtype	Coefficient	P-value
**Acinar-ness >0**	0.88	0.66
**Solid-ness >0**	1.92	0.0020
**Papillary-ness >0**	0.89	0.57
**CIS-ness >0**	0.85	0.45

Using a multivariate analysis we found that the presence of any solid component lead to twice the hazard as compared to tumors without a solid component.

### Validation

In the Japanese cohort of 87 AD and in the French cohort of 89 AD, hierarchical clustering using the same parameters as described for the current analysis also produced three clusters. A significant difference was also found between survival rates of the different clusters via a log-rank test (p<0.001) in the Japanese cohort. Cluster 1 had significantly better survival than the other two clusters, with hazard ratios of greater than five in both cases (Cox-PH p-values <0.002 for each). Similarly, in the French cohort three clusters were found with a significant difference between survival rates (p<0.001). Using the same methods as mentioned above, pathway expression was summarized using the mean, and models were fit using these pathways as predictors. For both validation sites, logistic regression models on cluster membership could not be fit apparently due to an insufficient amount of data in these smaller cohorts. Gene enrichment on each set of data however, showed significant enrichment of the cell cycle stimulatory pathway and embryonic stem cell (ESC) pathway in one cluster (p<0.001 in each set of data). Differences between clusters were also seen in apoptosis (stimulatory and suppressive) and immune response (such as antigen) pathways in each set of data. Additional gene enrichment results can be seen in the Supplemental Data as Table S10.

In the pathway survival model using the Japanese cohort, the cell cycle stimulatory pathway (CC+) was found to be significantly related to poorer outcome (hazard ratio 1.61, p = 0.02) and the B-cell pathway marginally related to better outcome (hazard ratio 0.68, p = 0.14), which give evidence to support our results. Interactions were again not fit due to the smaller amount of data. In the French cohort, which consisted of mostly stage 1 cancers, the IL-suppressive pathway was found to be significantly related to poorer outcome (hazard ratio 3.78, p<0.001), the mTOR pathway was found to be significantly related to poorer outcome (hazard ratio 2.41, p = 0.004), and the antigen pathway was found to be significantly related to better survival (hazard ratio 0.19, p = 0.002) after adjusting for the other pathways in the final model. Once again, interactions were not included in the model due to the smaller sample size.

Gaussian regression models were fit to the percent of each AD subtype, as determined by pathological review for both the Japanese and French AD cohorts. As in our analyses, in the Japanese cohort AD with higher cell cycle stimulatory pathway expression tend to be more of the solid subtype (p<0.001) and less of the papillary and CIS subtypes (p = 0.02 and p<0.001 respectively), after adjusting for other pathways. Also, tumors higher in Wnt expression tended to contain less of the solid subtype (p<0.01), other pathways held constant. In the French cohort, AD with higher cell cycle stimulatory pathway expression tended to be marginally more acinar (p = 0.08), tumors with higher mTOR pathway expression were significantly more of the solid subtype (p = 0.009), and tumors with higher IL-stimulatory pathway expression had significantly less CIS and papillary components (p = 0.002 and p<0.0001 respectively), with significantly more acinar and solid components (p = 0.01 and p<0.0001 respectively). We provide qualitative assessments of both the pathological and pathway correspondence between the three datasets in Table 7. Also see validation results: Figure S1, Figure S2, Figure S3, Figure S4, Figure S5, Table S1, Table S2, Table S3, Table S4, Table S5, Table S6, Table S7, Table S8 and Table S9.

**Table 7:** Overall Qualitative Summary.

	Survival Relationship			Pathology Relationship		
Pathway	Michigan	France	Japan	Michigan	France	Japan
**Cell Cycle (+)**	*X (poor)*		*X (poor)*	*CIS (−);* Acinar (+); *Pap (−); Solid (+)*		*CIS (−); Pap (−); Solid (+)*
**ESC**						
**B-cell**	X (good)			Acinar (−)		
**T-cell**						
**Antigen**	X(good)					
**AKT/PI3K**		*X (poor)*	*X (poor)*		Pap (−)	Acinar (+); Solid (−)
**IGF-1**		X (good)		*Acinar (+);* Pap (−)		*Acinar (+);* Pap (+)
**Chemokine**						
**NF-κB**						
**Notch**	X (poor)			Pap (+); Solid (−)		Acinar (+)
**JAK/STAT**		X (poor)				
**Complement**			X (good)	Acinar (+); *Solid (−)*		Pap (+); *Solid (−)*
**mTOR**		*X (poor)*	*X (poor)*	Acinar (−)		Solid (+)
**Cell Cycle (−)**	X (good)				Acinar (−)	Acinar (+); Pap (−)
**Angiogenesis**		X (poor)		*Acinar (−);* Pap (−); Solid (+)	CIS (+); *Acinar (−)*	CIS (−)
**IL-stimulatory**		X (poor)		*CIS (−)*	*CIS (−);* Acinar (+); Pap (+); Solid (−)	Acinar (−)
**IL-suppressive**	X (poor)					
**Interferon**		X (good)				Pap (+)
**EGFR**	X (good)			Acinar (−); *Pap (+)*	*Pap (+)*	
**PDGF**			X (poor)	*Acinar (+)*	*Acinar (+)*	CIS (+); Pap (−); Solid (+)
**Hypoxia**	X (poor)				Pap (+)	Acinar (+)
**PTEN**						
**Pro-apoptosis**		X (good)				CIS (−)
**Anti-apoptosis**				Solid (+)		
**TGF-β**						Pap (−); Solid (+)
**Hedgehog**	X (poor)			Pap (+); Solid (−)		
**Wnt**				*Solid (−)*	Acinar (+); *Solid (−)*	*Solid (−)*

An overall qualitative summary. Items in *italics* indicate validation in two or three of the three datasets. Abbreviations: poor, statistically poor survival; good, statistically good survival; CIS, carcinoma *in situ*; Pap, papillary; (+), statistically significant positive association between pathway and given subtype; (−), statistically significant negative association between pathway and given subtype.

## Discussion

Lung adenocarcinomas (AD) are highly heterogeneous demonstrating a large number of genetic alterations [4], [5] and several well-recognized pathological subtypes [6]. A better understanding of this heterogeneity and potential clinical-pathological relationships is a necessary step in identifying new strategies for effectively treating patient subgroups. Although prior analyses of AD using gene expression have often revealed three subgroups [12], [13] no studies have integrated clinical covariates, pathological subtype [3]–[5], [7] and gene expression-based pathway analyses. Based on unsupervised analysis using 16,660 genes and the large AD dataset of 432 tumors, we observed three separate clusters of tumors. We found a significant difference in survival between clusters of tumors suggesting that tumors are meaningfully classified by their common features of gene expression while “major pathological subtype” categorization alone does not. Gene enrichment showed that of the selected pathways we examined, cell proliferation and immune response pathways were most responsible for the separation of the three clusters (see Table 2). Further analyses of additional pathways indicate that the tumors that comprise these separate groups largely share pathway expression profiles (see Table 3). Beyond the survival differences between clusters, a Cox proportional-hazards model also gave prognostic profiles based on the pathways that were, in addition to that given by stage and age, indicating the existence of further subgroups not captured by the hierarchical clustering.

We found relationships between pathways and the pathological-based subtypes of the AD tumors (see Table 5). This suggests that there are differences between AD subtypes in the activation and expression of cancer-related pathways. Among the most intriguing is the EGFR-papillary AD subtype connection, previously proposed [6]. Moreover, papillary tumors, which are characterized by papillae, tend to overexpress the hedgehog pathway. Hedgehog signaling is known to lead to the bifurcation of structures during development, which may influence this morphology [14]. In addition, solid tumors are best characterized by the highly-significant, over-expression of the cell cycle stimulatory (CC+) pathway and that tumors with any solid component had significantly higher hazard as compared to those without a solid component. They do not, however, show a strong relationship to a specific pathway indicating perhaps that there is a great deal of variability in which pathway is driving the cellular proliferation, yet the common feature is the increase in cell proliferation. Independent qualitative validation on two AD cohorts showed a group of tumors in each set with higher expression of cell proliferation genes than the other tumors. In the Japanese AD cohort, tumors with the cell cycle positive (CC+) pathway were associated with poorer outcome after adjusting for other pathways and for clinical variables such as stage. In the French cohort containing mostly stage 1 AD, this pathway was not significantly prognostic however we did not see a significant interaction with stage in our data so this could be due to the smaller sample in that validation set. Immune response pathways such as B-cell, complement, and antigen were found to be predictive of better survival in all three sets of AD tumors. Overall, it appears that cell proliferation and immune response combine to form a common predictor of survival, although it also appears that there is heterogeneity in which pathways make up a particular tumor's profile.

In addition to the survival validation observed in our analyses, we also saw common characteristics among AD subtypes, such as increased cell proliferation pathway expression and a greater solid component also detected in the Japanese cohort analyses. Further, the EGFR-papillary connection was also detected in the French AD cohort at a similar magnitude as it was found in the North American ADs, although interestingly this was not the case in the Japanese AD cohort. It is known that EGFR mutations are much more frequent in the Asian population of lung AD and this may influence the association with a given subtype however this result is interesting and worthy of additional investigation. The validation analyses also showed the heterogeneity of tumors across data sets and possibly across regions of the World. For example, in our set of North American AD, mTOR was not found to be an important predictor of survival (due to a high correlation with pathways that were not selected out of the model), but in both validation sets higher expression of the mTOR pathway was associated with poorer outcome which is consistent with previous reports [15]. Also, increased cell proliferation and a larger solid component was highly significant in both the North American and the Japanese lung cohorts, although not in the mostly stage 1 French cohort.

These relationships provide an interesting opportunity to examine interactions between tumor pathology and active pathway affecting the progression of the relatively good outcome CIS lesions to those with more poor outcome and often solid morphology. Two of the three datasets showed reduced cell cycle activity and an increase in the interleukin stimulatory pathway (IL+) with elevated CIS component. However, in two of three datasets (as mentioned above) there was an association between increased EGFR and reduced cell cycle activity and the percentage of papillary component. This potentially indicates that papillary tumors progress from CIS tumors resulting from an increase in EGFR pathway expression. Similarly, progression from CIS to acinar may be driven by increased PDGF and decreased angiogenic pathway activity. Solid component tumors showed an association with increased CC+ and decreased complement pathways in two of the three datasets while all three datasets showed an association between solid component and decreased Wnt signaling. Clearly, additional studies are needed to further validate these findings yet they represent interesting data in light of the vast heterogeneity of AD.

It is important to note that our analyses are an attempt to describe meaningful differences between AD, rather than an attempt to assess each individual pathway's importance. For example, we identified the set of pathways that best predicts survival instead of the univariate survival significance of each pathway. This would not imply that a pathway not found to be a significant predictor of survival in our multivariate analyses lacks clinical significance, just that the survival differences were better explained by a set of other pathways. In general however, across AD and representing different regions of the world, many aspects of the profiles of AD are remarkably similar. This along with more uniform classification will allow potential new therapeutic strategies to be developed for lung adenocarcinomas.

## Supporting Information

Figure S1 French validation of clustering results.
(0.29 MB TIF)

Figure S2 French validation of survival differences of clusters.
(0.10 MB TIF)

Figure S3 French validation of solid subtype survival differences.
(0.08 MB TIF)

Figure S4 Japanese validation of clustering results.
(2.45 MB TIF)

Figure S5 Japanese validation of cluster, stage, grade and sex survival differences.
(0.27 MB TIF)

R code S1 Complete R code of methods.
(0.23 MB DOC)

References S1 References for gene lists
(0.05 MB DOC)

Gene Lists S1 Gene lists for all pathways with probe sets.
(1.09 MB XLS)

Table S1 French validation cluster descriptives.
(0.04 MB DOC)

Table S2 French gene enrichment p-values.
(0.05 MB DOC)

Table S3 French validation of pathway survival.
(0.03 MB DOC)

Table S4 French validation of pathway-pathology interactions.
(0.06 MB DOC)

Table S5 Japanese cluster descriptives.
(0.04 MB DOC)

Table S6 Japanese gene enrichment p-values.
(0.04 MB DOC)

Table S7 Japanese pathway survival.
(0.03 MB DOC)

Table S8 Japanese pathway-pathology interactions.
(0.03 MB DOC)

Table S9 Validation overall statistics
(0.03 MB DOC)

Table S10 Additional United States gene enrichment data.
(0.06 MB DOC)

Table S11 Additional United States pathology-pathway data - CIS (+/−).
(0.05 MB DOC)

Table S12 Additional United States pathology-pathway data - acinar (+/−).
(0.04 MB DOC)

Table S13 Additional United States pathology-pathway data - papillary (+/−).
(0.04 MB DOC)

Table S14 Additional United States pathology-pathway data - solid (+/−).
(0.04 MB DOC)

Table S15 United States tests for proportionality of hazards.
(0.04 MB DOC)

Table S16 United States multivariate Pathology Survival using Percent Component
(0.03 MB DOC)
