# Supplementary figures and images for: Clinically Relevant Characterization of Lung Adenocarcinoma Subtypes Based on Cellular Pathways: An International Validation Study
Source: PLoS One. 2010 Jul 22;5(7):e11712. doi: 10.1371/journal.pone.0011712 (PMC2908611; doi:10.1371/journal.pone.0011712)

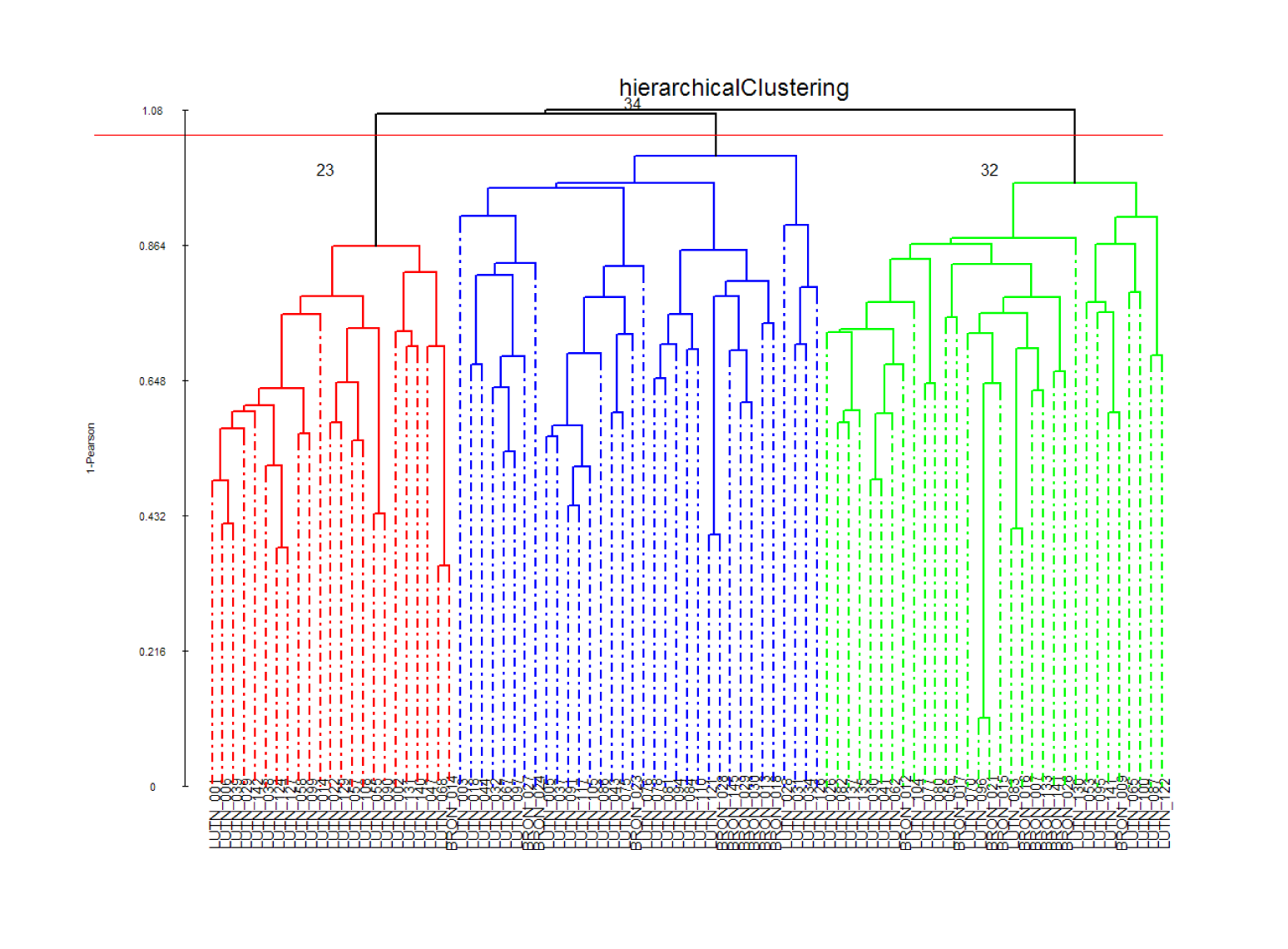

Supplement: Figure S1 — French validation of clustering results. (0.29 MB TIF) [file pone.0011712.s001.tif]

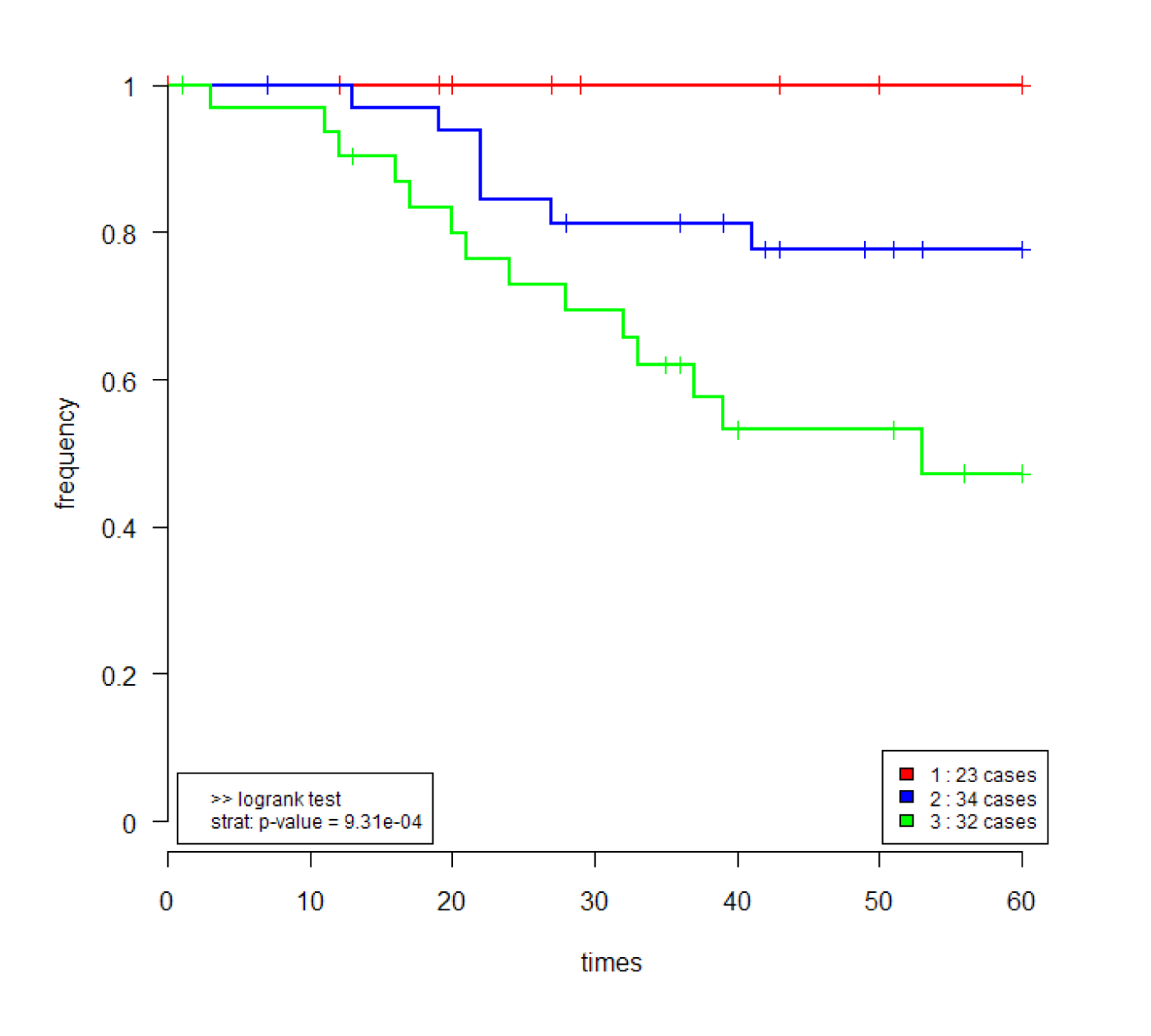

Supplement: Figure S2 — French validation of survival differences of clusters. (0.10 MB TIF) [file pone.0011712.s002.tif]

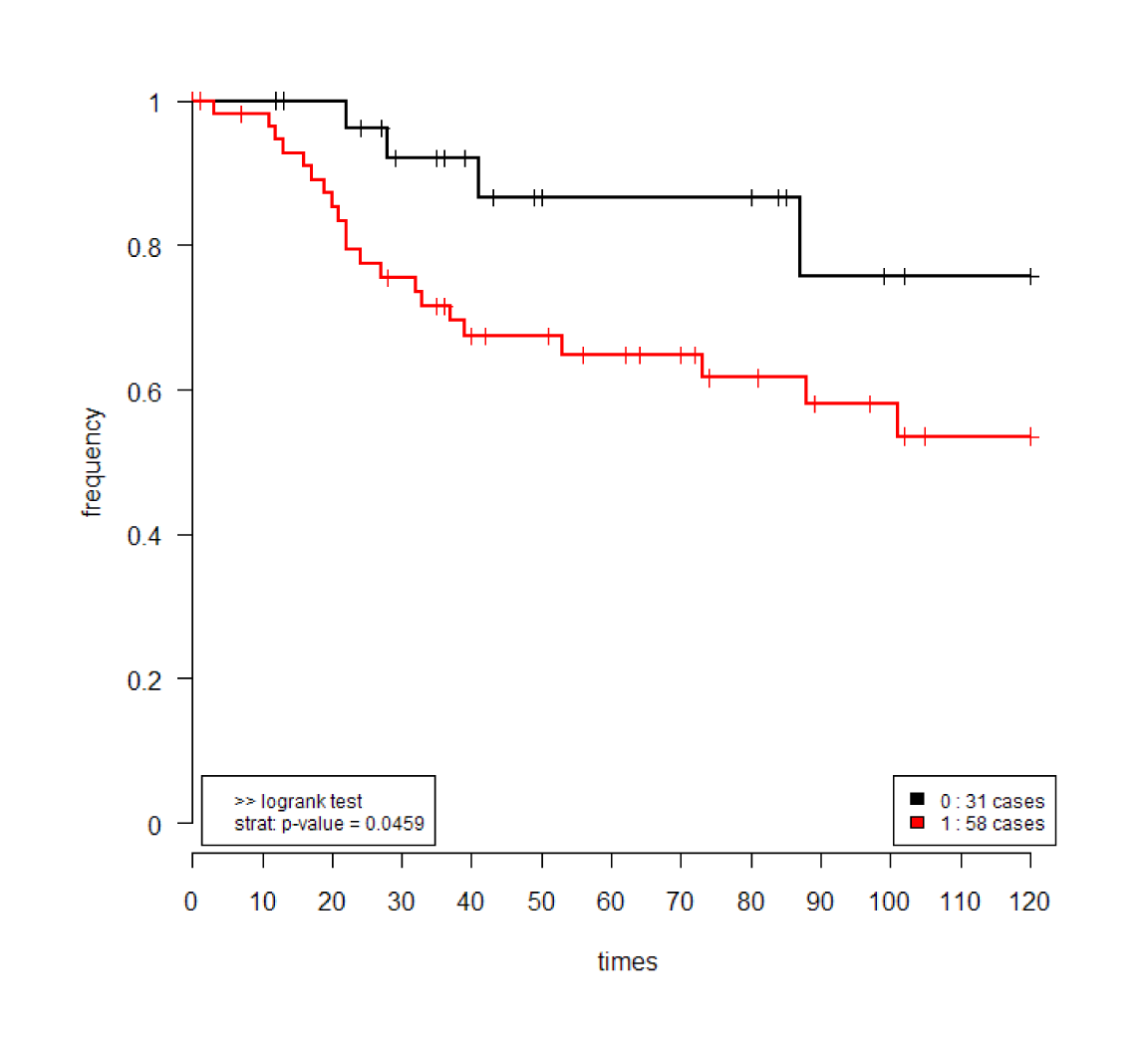

Supplement: Figure S3 — French validation of solid subtype survival differences. (0.08 MB TIF) [file pone.0011712.s003.tif]

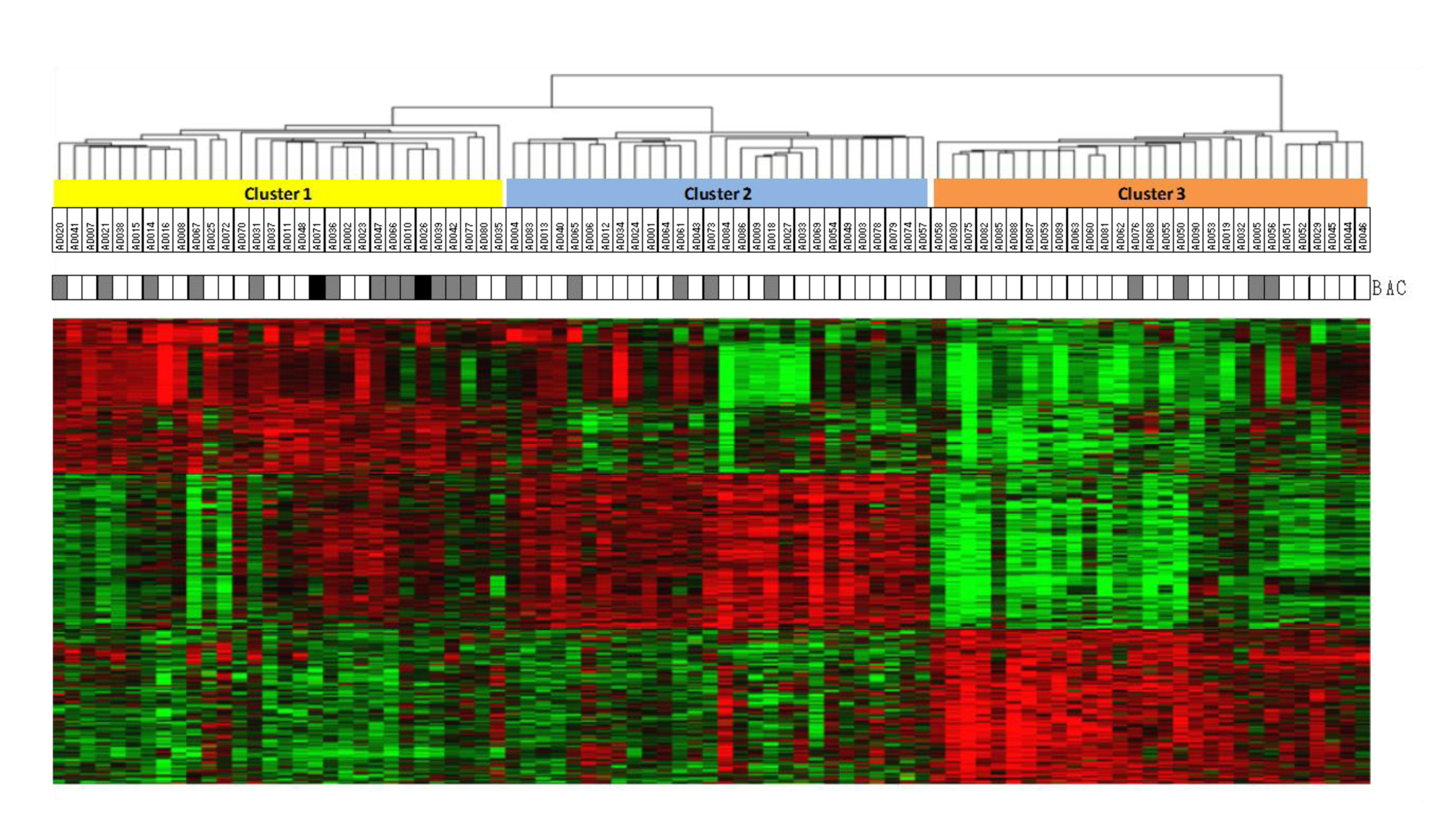

Supplement: Figure S4 — Japanese validation of clustering results. (2.45 MB TIF) [file pone.0011712.s004.tif]

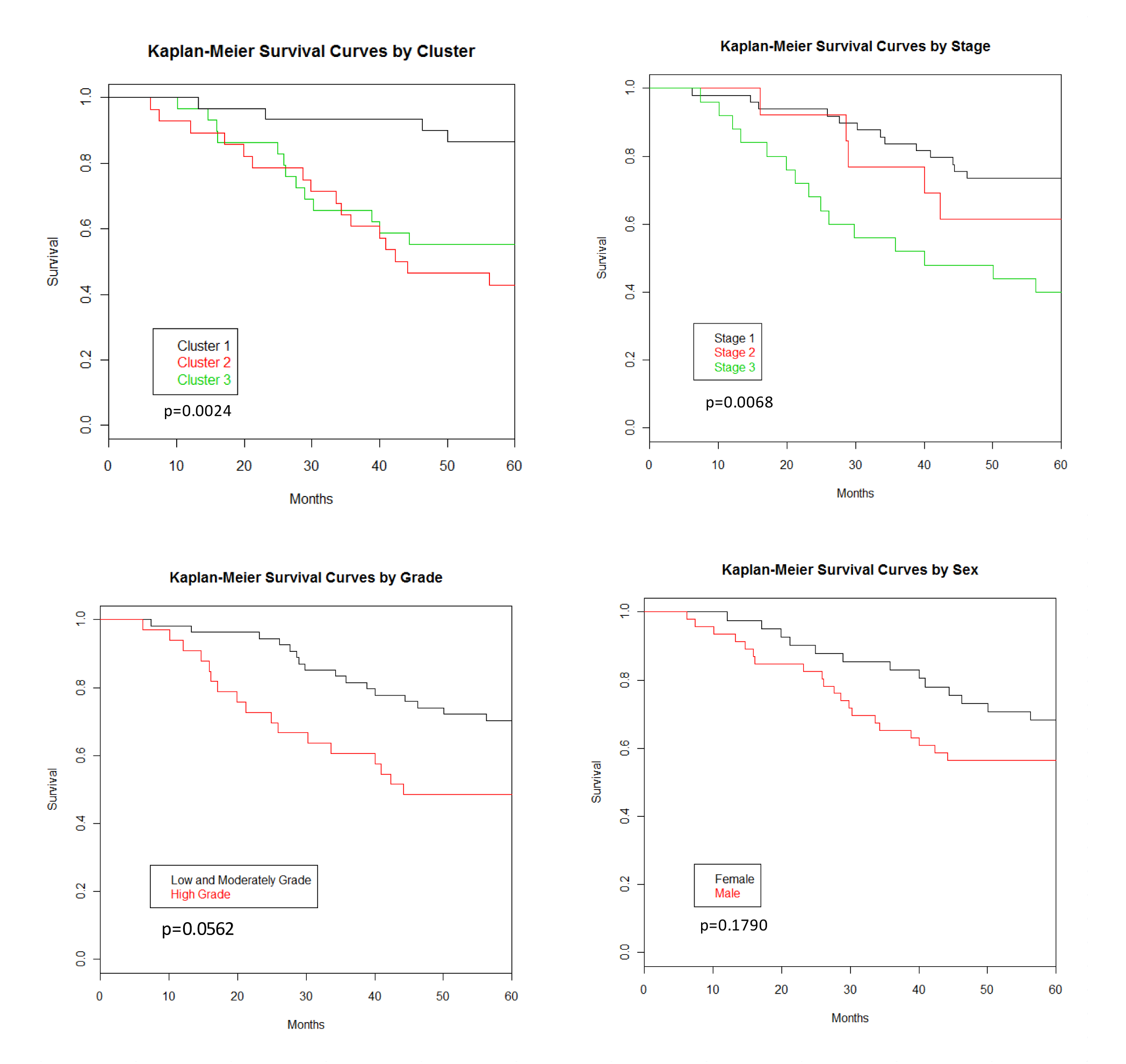

Supplement: Figure S5 — Japanese validation of cluster, stage, grade and sex survival differences. (0.27 MB TIF) [file pone.0011712.s005.tif]
