# Supplementary material for: Clinically Relevant Characterization of Lung Adenocarcinoma Subtypes Based on Cellular Pathways: An International Validation Study
Source: PLoS One. 2010 Jul 22;5(7):e11712. doi: 10.1371/journal.pone.0011712 (PMC2908611; doi:10.1371/journal.pone.0011712)
Supplement: R code S1 — Complete R code of methods. (0.23 MB DOC) [file pone.0011712.s006.doc]

**R Code**

######################### Log-transform, mean center genes within each site, then combine #########################

midata <- read.csv(file=" /Desktop/Data/2008-11-06 - MI Tumors.csv", head=TRUE, row.names=1, sep=",", dec=".")

dim(midata)

midata <- midata + 1

midata <- log2(midata)

midata <- t(scale(t(midata), center=TRUE, scale=FALSE))

midata[1:5,1:5]

hlmdata <- read.csv(file=" /Desktop/Data/2008-11-05 - HLM all genes.csv", head=TRUE, row.names=1, sep=",", dec=".")

dim(hlmdata)

hlmdata <- hlmdata + 1

hlmdata <- log2(hlmdata)

hlmdata <- t(scale(t(hlmdata), center=TRUE, scale=FALSE))

hlmdata[1:5,1:5]

dfdata <- read.csv(file=" /Desktop/Data/2008-11-05 - DF all genes.csv", head=TRUE, row.names=1, sep=",", dec=".")

dim(dfdata)

dfdata <- dfdata + 1

dfdata <- log2(dfdata)

dfdata <- t(scale(t(dfdata), center=TRUE, scale=FALSE))

dfdata[1:5,1:5]

mskdata <- read.csv(file=" /Desktop/Data/2008-11-05 - MSK all genes.csv", head=TRUE, row.names=1, sep=",", dec=".")

dim(mskdata)

mskdata <- mskdata + 1

mskdata <- log2(mskdata)

mskdata <- t(scale(t(mskdata), center=TRUE, scale=FALSE))

mskdata[,35]

mskdata <- mskdata[,-35]

alldata <- mat.or.vec(22214,432)

alldata[,1:167] <- as.matrix(midata)

alldata[1:5,1:5]

alldata[,168:246] <- as.matrix(hlmdata)

alldata[,247:328] <- as.matrix(dfdata)

alldata[,329:432] <- as.matrix(mskdata)

row.names(alldata) <- rownames(midata)

write.csv(alldata, file=" /Desktop/Data/2008-11-06 - All genes and tumors.csv", row.names=TRUE)

######################### Read in the reduced (in Excel) data with 75% most-varying genes, and also read in the #########################

######################### cluster indicator variables #########################

alldata <- read.csv(file=" /Desktop/Data/2008-11-06 - All genes and tumors, reduced.csv", head=TRUE, row.names=1, sep=",", dec=".")

clusters <- read.csv(file=" /Desktop/Data/2008-11-11 - Final Tumor Clusters.csv", head=TRUE, sep=",", dec=".")

inclus1 <- clusters[,3]

inclus2 <- clusters[,4]

inclus3 <- clusters[,5]

cluster <- inclus1+2*inclus2+3*inclus3

alldata <- as.matrix(alldata)

######################### Tests for genes significantly up and down in each cluster #########################

clus1uptests <- mat.or.vec(16660,1)

for(i in 1:16660){clus1uptests[i] <- cor.test(alldata[i,], inclus1, alternative="greater", method="pearson")[3]}

clus1uptests <- as.matrix(as.numeric(clus1uptests))

row.names(clus1uptests) <- rownames(alldata)

clus1uptestsorted <- apply(clus1uptests, 2, sort, index.return=FALSE)

clus1downtests <- mat.or.vec(16660,1)

for(i in 1:16660){clus1downtests[i] <- cor.test(alldata[i,], inclus1, alternative="less", method="pearson")[3]}

clus1downtests <- as.matrix(as.numeric(clus1downtests))

row.names(clus1downtests) <- rownames(alldata)

clus1downtestsorted <- apply(clus1downtests, 2, sort, index.return=FALSE)

clus2uptests <- mat.or.vec(16660,1)

for(i in 1:16660){clus2uptests[i] <- cor.test(alldata[i,], inclus2, alternative="greater", method="pearson")[3]}

clus2uptests <- as.matrix(as.numeric(clus2uptests))

row.names(clus2uptests) <- rownames(alldata)

clus2uptestsorted <- apply(clus2uptests, 2, sort, index.return=FALSE)

clus2downtests <- mat.or.vec(16660,1)

for(i in 1:16660){clus2downtests[i] <- cor.test(fdata[i,], inclus2, alternative="less", method="pearson")[3]}

clus2downtests <- as.matrix(as.numeric(clus2downtests))

row.names(clus2downtests) <- rownames(alldata)

clus2downtestsorted <- apply(clus2downtests, 2, sort, index.return=FALSE)

clus3uptests <- mat.or.vec(16660,1)

for(i in 1:16660){clus3uptests[i] <- cor.test(alldata[i,], inclus3, alternative="greater", method="pearson")[3]}

clus3uptests <- as.matrix(as.numeric(clus3uptests))

row.names(clus3uptests) <- rownames(alldata)

clus3uptestsorted <- apply(clus3uptests, 2, sort, index.return=FALSE)

clus3downtests <- mat.or.vec(16660,1)

for(i in 1:16660){clus3downtests[i] <- cor.test(alldata[i,], inclus3, alternative="less", method="pearson")[3]}

clus3downtests <- as.matrix(as.numeric(clus3downtests))

row.names(clus3downtests) <- rownames(alldata)

clus3downtestsorted <- apply(clus3downtests, 2, sort, index.return=FALSE)

######################### Read in survival data #########################

misurv <- read.csv(file="/Desktop/Data/2008-11-14- MI Clinical.csv", row.names=1, header=FALSE)

restsurv <- read.csv(file="/Desktop/Data/2008-11-14 - Test Set Clinical Vars.csv", header=TRUE)

misurv <- as.matrix(misurv)

months1 <- as.numeric(misurv[2,])

dead1 <- as.numeric(misurv[3,])

dead2 <- mat.or.vec(265,1)

for(i in 1:265){if(restsurv[i,12]=="Dead") dead2[i] <- 1 else dead2[i] <- 0}

months2 <- as.numeric(restsurv[,16])

months <- mat.or.vec(432,1)

dead <- mat.or.vec(432,1)

months[1:167] <- months1

months[168:432] <- months2

dead <- mat.or.vec(432,1)

dead[1:167] <- dead1

dead[168:432] <- dead2

######################### 5-year survival variables #########################

months5 <- months

dead5 <- dead

for(i in 1:99){if(months[i]>60) (months5[i] <- 60) & (dead5[i] <- 0)}

for(i in 101:432){if(months[i]>60) (months5[i] <- 60) & (dead5[i] <- 0)}

######################### Categorical variable for site #########################

site <- mat.or.vec(432,1)

site[1:167] <- 1

site[168:246] <- 2

site[247:328] <- 3

site[329:432] <- 4

site <- as.factor(site)

######################### Stage (1-3) #########################

stage2 <- mat.or.vec(265,1)

nstage <- restsurv[,19]

tstage <- restsurv[,20]

for(i in 1:265){if((nstage[i]=="N0, ACCORDING TO AJCC CRITERIA" & tstage[i]=="T1, ACCORDING TO AJCC CRITERIA") | (nstage[i]=="N0, ACCORDING TO AJCC CRITERIA" & tstage[i]=="T2, ACCORDING TO AJCC CRITERIA")) stage2[i] <- 1 else if((nstage[i]=="N1, ACCORDING TO AJCC CRITERIA" & tstage[i]=="T1, ACCORDING TO AJCC CRITERIA") | (nstage[i]=="N1, ACCORDING TO AJCC CRITERIA" & tstage[i]=="T2, ACCORDING TO AJCC CRITERIA") | (nstage[i]=="N0, ACCORDING TO AJCC CRITERIA" & tstage[i]=="T3, ACCORDING TO AJCC CRITERIA")) stage2[i] <- 2 else stage2[i] <- 3}

stage2[7] <- NA

stage2[11] <- NA

stage1 <- as.numeric(misurv[4,])

stage <- mat.or.vec(432,1)

stage[1:167] <- stage1

stage[168:432] <- stage2

stage <- as.factor(stage)

######################### Age #########################

age1 <- as.numeric(misurv[7,])

age2 <- as.numeric(restsurv[,8])

age <- mat.or.vec(432,1)

age[1:167] <- age1

age[168:432] <- age2

######################### Grade (1=poorly differentiated) #########################

grade1 <- as.numeric(misurv[5,])

grade2 <- mat.or.vec(265,1)

for(i in 1:265){if(restsurv[i,25]=="POORLY DIFFERENTIATED") grade2[i] <- 1 else (if(restsurv[i,25]=="") grade2[i] <- NA else grade2[i] <- 0)}

grade <- mat.or.vec(265,1)

grade[1:167] <- grade1

grade[168:432] <- grade2

######################### Gender (1=male) #########################

male1 <- as.numeric(misurv[8,])

male2 <- as.character(restsurv[,7])

for(i in 1:265){if(male2[i]=="Male") male2[i] <- 1 else male2[i] <- 0}

male2 <- as.numeric(male2)

male <- mat.or.vec(432,1)

male[1:167] <- male1

male[168:432] <- male2

######################### Kaplan-Meier 5-year survival curves for each cluster, with associated logrank test #########################

survdiff(Surv(months5,dead5)~cluster)

plot(survfit(Surv(months,dead)~cluster), col=c(1,2,3), xlim=c(0,60), main="Kaplan-Meier Survival Curves by Cluster", ylab="Survival", xlab="Months")

leg.txt <- c("Cluster 1", "Cluster 2", "Cluster 3")

legend(locator(1), leg.txt, text.col=c(1,2,3))

######################### Pathway expression values (created in Excel) #########################

mgs <- read.csv(file="/Desktop/Data/2009-03-03 - Final Pathways.csv", header=TRUE, row.names=1)

mgs <- as.matrix(mgs)

######################### Additional clinical file with subtype percentages #########################

moreclin <- read.csv(file="/Desktop/Data/2009-02-01 - Final clustering as ordered with stage, grade, pathology.csv", header=TRUE)

subtype <- moreclin$Most.prominent.subtype..MPS

solidness <- as.numeric(as.character(moreclin$X..solid))

bacness <- as.numeric(as.character(moreclin$X..BAC))

papness <- as.numeric(as.character(moreclin$X..Papillary))

acinarness <- as.numeric(as.character(moreclin$X..acinar))

mgs1 <- t(mgs)

mgs2 <- as.data.frame(cbind(mgs1, site, stage, grade, age, male, subtype, solidness, bacness, papness, acinarness, months, dead, months5, dead5, cluster))

mgs2$site <- factor(mgs2$site)

mgs2$stage <- factor(mgs2$stage)

mgs2$subtype <- factor(mgs2$subtype)

mgs2$cluster <- factor(mgs2$cluster)

# Remove na's to perform analyses with subtype

mgs3 <- na.omit(mgs2)

# Remove na's to perform analyses without subtype

mgs4 <- (mgs2[,-33])

mgs4 <- (mgs4[,-33])

mgs4 <- (mgs4[,-33])

mgs4 <- (mgs4[,-33])

mgs4 <- na.omit(mgs4[,-33])

# Data frame of original Pathways, no na's removed

mgs5 <- as.data.frame(mgs1)

######################### Regressions to remove multicollinearity #########################

reg1 <- lm(ESC~mTOR+Ccneg+IFN+Ilpos+Ilneg+EGFR+PGDF+Ang+Hypoxia+AKT+IGF+PTEN+Bcell+Tcell+Antigen+Comp+Wnt+Hh+TGFB+JAKSTAT+Notch+NFKB+Ccpos+Chemokine+Antiapop+Proapop, data=mgs5)

reg2 <- lm(mTOR~ESC+Ccneg+IFN+Ilpos+Ilneg+EGFR+PGDF+Ang+Hypoxia+AKT+IGF+PTEN+Bcell+Tcell+Antigen+Comp+Wnt+Hh+TGFB+JAKSTAT+Notch+NFKB+Ccpos+Chemokine+Antiapop+Proapop, data=mgs5)

reg3 <- lm(Ccneg~mTOR+ESC+IFN+Ilpos+Ilneg+EGFR+PGDF+Ang+Hypoxia+AKT+IGF+PTEN+Bcell+Tcell+Antigen+Comp+Wnt+Hh+TGFB+JAKSTAT+Notch+NFKB+Ccpos+Chemokine+Antiapop+Proapop, data=mgs5)

reg4 <- lm(IFN~Ccneg+mTOR+ESC+Ilpos+Ilneg+EGFR+PGDF+Ang+Hypoxia+AKT+IGF+PTEN+Bcell+Tcell+Antigen+Comp+Wnt+Hh+TGFB+JAKSTAT+Notch+NFKB+Ccpos+Chemokine+Antiapop+Proapop, data=mgs5)

reg5 <- lm(Ilpos~IFN+Ccneg+mTOR+ESC+Ilneg+EGFR+PGDF+Ang+Hypoxia+AKT+IGF+PTEN+Bcell+Tcell+Antigen+Comp+Wnt+Hh+TGFB+JAKSTAT+Notch+NFKB+Ccpos+Chemokine+Antiapop+Proapop, data=mgs5)

reg6 <- lm(Ilneg~Ilpos+IFN+Ccneg+mTOR+ESC+EGFR+PGDF+Ang+Hypoxia+AKT+IGF+PTEN+Bcell+Tcell+Antigen+Comp+Wnt+Hh+TGFB+JAKSTAT+Notch+NFKB+Ccpos+Chemokine+Antiapop+Proapop, data=mgs5)

reg7 <- lm(EGFR~Ilneg+Ilpos+IFN+Ccneg+mTOR+ESC+PGDF+Ang+Hypoxia+AKT+IGF+PTEN+Bcell+Tcell+Antigen+Comp+Wnt+Hh+TGFB+JAKSTAT+Notch+NFKB+Ccpos+Chemokine+Antiapop+Proapop, data=mgs5)

reg8 <- lm(PGDF~EGFR+Ilneg+Ilpos+IFN+Ccneg+mTOR+ESC+Ang+Hypoxia+AKT+IGF+PTEN+Bcell+Tcell+Antigen+Comp+Wnt+Hh+TGFB+JAKSTAT+Notch+NFKB+Ccpos+Chemokine+Antiapop+Proapop, data=mgs5)

reg9 <- lm(Ang~PGDF+EGFR+Ilneg+Ilpos+IFN+Ccneg+mTOR+ESC+Hypoxia+AKT+IGF+PTEN+Bcell+Tcell+Antigen+Comp+Wnt+Hh+TGFB+JAKSTAT+Notch+NFKB+Ccpos+Chemokine+Antiapop+Proapop, data=mgs5)

reg10 <- lm(Hypoxia~Ang+PGDF+EGFR+Ilneg+Ilpos+IFN+Ccneg+mTOR+ESC+AKT+IGF+PTEN+Bcell+Tcell+Antigen+Comp+Wnt+Hh+TGFB+JAKSTAT+Notch+NFKB+Ccpos+Chemokine+Antiapop+Proapop, data=mgs5)

reg11 <- lm(AKT~Hypoxia+Ang+PGDF+EGFR+Ilneg+Ilpos+IFN+Ccneg+mTOR+ESC+IGF+PTEN+Bcell+Tcell+Antigen+Comp+Wnt+Hh+TGFB+JAKSTAT+Notch+NFKB+Ccpos+Chemokine+Antiapop+Proapop, data=mgs5)

reg12 <- lm(IGF~AKT+Hypoxia+Ang+PGDF+EGFR+Ilneg+Ilpos+IFN+Ccneg+mTOR+ESC+PTEN+Bcell+Tcell+Antigen+Comp+Wnt+Hh+TGFB+JAKSTAT+Notch+NFKB+Ccpos+Chemokine+Antiapop+Proapop, data=mgs5)

reg13 <- lm(PTEN~IGF+AKT+Hypoxia+Ang+PGDF+EGFR+Ilneg+Ilpos+IFN+Ccneg+mTOR+ESC+Bcell+Tcell+Antigen+Comp+Wnt+Hh+TGFB+JAKSTAT+Notch+NFKB+Ccpos+Chemokine+Antiapop+Proapop, data=mgs5)

reg14 <- lm(Bcell~PTEN+IGF+AKT+Hypoxia+Ang+PGDF+EGFR+Ilneg+Ilpos+IFN+Ccneg+mTOR+ESC+Tcell+Antigen+Comp+Wnt+Hh+TGFB+JAKSTAT+Notch+NFKB+Ccpos+Chemokine+Antiapop+Proapop, data=mgs5)

reg15 <- lm(Tcell~Bcell+PTEN+IGF+AKT+Hypoxia+Ang+PGDF+EGFR+Ilneg+Ilpos+IFN+Ccneg+mTOR+ESC+Antigen+Comp+Wnt+Hh+TGFB+JAKSTAT+Notch+NFKB+Ccpos+Chemokine+Antiapop+Proapop, data=mgs5)

reg16 <- lm(Antigen~Tcell+Bcell+PTEN+IGF+AKT+Hypoxia+Ang+PGDF+EGFR+Ilneg+Ilpos+IFN+Ccneg+mTOR+ESC+Comp+Wnt+Hh+TGFB+JAKSTAT+Notch+NFKB+Ccpos+Chemokine+Antiapop+Proapop, data=mgs5)

reg17 <- lm(Comp~Antigen+Tcell+Bcell+PTEN+IGF+AKT+Hypoxia+Ang+PGDF+EGFR+Ilneg+Ilpos+IFN+Ccneg+mTOR+ESC+Wnt+Hh+TGFB+JAKSTAT+Notch+NFKB+Ccpos+Chemokine+Antiapop+Proapop, data=mgs5)

reg18 <- lm(Wnt~Comp+Antigen+Tcell+Bcell+PTEN+IGF+AKT+Hypoxia+Ang+PGDF+EGFR+Ilneg+Ilpos+IFN+Ccneg+mTOR+ESC+Hh+TGFB+JAKSTAT+Notch+NFKB+Ccpos+Chemokine+Antiapop+Proapop, data=mgs5)

reg19 <- lm(Hh~Wnt+Comp+Antigen+Tcell+Bcell+PTEN+IGF+AKT+Hypoxia+Ang+PGDF+EGFR+Ilneg+Ilpos+IFN+Ccneg+mTOR+ESC+TGFB+JAKSTAT+Notch+NFKB+Ccpos+Chemokine+Antiapop+Proapop, data=mgs5)

reg20 <- lm(TGFB~Hh+Wnt+Comp+Antigen+Tcell+Bcell+PTEN+IGF+AKT+Hypoxia+Ang+PGDF+EGFR+Ilneg+Ilpos+IFN+Ccneg+mTOR+ESC+JAKSTAT+Notch+NFKB+Ccpos+Chemokine+Antiapop+Proapop, data=mgs5)

reg21 <- lm(JAKSTAT~TGFB+Hh+Wnt+Comp+Antigen+Tcell+Bcell+PTEN+IGF+AKT+Hypoxia+Ang+PGDF+EGFR+Ilneg+Ilpos+IFN+Ccneg+mTOR+ESC+Notch+NFKB+Ccpos+Chemokine+Antiapop+Proapop, data=mgs5)

reg22 <- lm(Notch~JAKSTAT+TGFB+Hh+Wnt+Comp+Antigen+Tcell+Bcell+PTEN+IGF+AKT+Hypoxia+Ang+PGDF+EGFR+Ilneg+Ilpos+IFN+Ccneg+mTOR+ESC+NFKB+Ccpos+Chemokine+Antiapop+Proapop, data=mgs5)

reg23 <- lm(NFKB~Notch+JAKSTAT+TGFB+Hh+Wnt+Comp+Antigen+Tcell+Bcell+PTEN+IGF+AKT+Hypoxia+Ang+PGDF+EGFR+Ilneg+Ilpos+IFN+Ccneg+mTOR+ESC+Ccpos+Chemokine+Antiapop+Proapop, data=mgs5)

reg24 <- lm(Ccpos~NFKB+Notch+JAKSTAT+TGFB+Hh+Wnt+Comp+Antigen+Tcell+Bcell+PTEN+IGF+AKT+Hypoxia+Ang+PGDF+EGFR+Ilneg+Ilpos+IFN+Ccneg+mTOR+ESC+Chemokine+Antiapop+Proapop, data=mgs5)

reg25 <- lm(Chemokine~Ccpos+NFKB+Notch+JAKSTAT+TGFB+Hh+Wnt+Comp+Antigen+Tcell+Bcell+PTEN+IGF+AKT+Hypoxia+Ang+PGDF+EGFR+Ilneg+Ilpos+IFN+Ccneg+mTOR+ESC+Antiapop+Proapop, data=mgs5)

reg26 <- lm(Antiapop~Chemokine+Ccpos+NFKB+Notch+JAKSTAT+TGFB+Hh+Wnt+Comp+Antigen+Tcell+Bcell+PTEN+IGF+AKT+Hypoxia+Ang+PGDF+EGFR+Ilneg+Ilpos+IFN+Ccneg+mTOR+ESC+Proapop, data=mgs5)

reg27 <- lm(Proapop~Antiapop+Chemokine+Ccpos+NFKB+Notch+JAKSTAT+TGFB+Hh+Wnt+Comp+Antigen+Tcell+Bcell+PTEN+IGF+AKT+Hypoxia+Ang+PGDF+EGFR+Ilneg+Ilpos+IFN+Ccneg+mTOR+ESC, data=mgs5)

# Removed ESC

reg2 <- lm(mTOR~Ccneg+IFN+Ilpos+Ilneg+EGFR+PGDF+Ang+Hypoxia+AKT+IGF+PTEN+Bcell+Tcell+Antigen+Comp+Wnt+Hh+TGFB+JAKSTAT+Notch+NFKB+Ccpos+Chemokine+Antiapop+Proapop, data=mgs5)

reg3 <- lm(Ccneg~mTOR+IFN+Ilpos+Ilneg+EGFR+PGDF+Ang+Hypoxia+AKT+IGF+PTEN+Bcell+Tcell+Antigen+Comp+Wnt+Hh+TGFB+JAKSTAT+Notch+NFKB+Ccpos+Chemokine+Antiapop+Proapop, data=mgs5)

reg4 <- lm(IFN~Ccneg+mTOR+Ilpos+Ilneg+EGFR+PGDF+Ang+Hypoxia+AKT+IGF+PTEN+Bcell+Tcell+Antigen+Comp+Wnt+Hh+TGFB+JAKSTAT+Notch+NFKB+Ccpos+Chemokine+Antiapop+Proapop, data=mgs5)

reg5 <- lm(Ilpos~IFN+Ccneg+mTOR+Ilneg+EGFR+PGDF+Ang+Hypoxia+AKT+IGF+PTEN+Bcell+Tcell+Antigen+Comp+Wnt+Hh+TGFB+JAKSTAT+Notch+NFKB+Ccpos+Chemokine+Antiapop+Proapop, data=mgs5)

reg6 <- lm(Ilneg~Ilpos+IFN+Ccneg+mTOR+EGFR+PGDF+Ang+Hypoxia+AKT+IGF+PTEN+Bcell+Tcell+Antigen+Comp+Wnt+Hh+TGFB+JAKSTAT+Notch+NFKB+Ccpos+Chemokine+Antiapop+Proapop, data=mgs5)

reg7 <- lm(EGFR~Ilneg+Ilpos+IFN+Ccneg+mTOR+PGDF+Ang+Hypoxia+AKT+IGF+PTEN+Bcell+Tcell+Antigen+Comp+Wnt+Hh+TGFB+JAKSTAT+Notch+NFKB+Ccpos+Chemokine+Antiapop+Proapop, data=mgs5)

reg8 <- lm(PGDF~EGFR+Ilneg+Ilpos+IFN+Ccneg+mTOR+Ang+Hypoxia+AKT+IGF+PTEN+Bcell+Tcell+Antigen+Comp+Wnt+Hh+TGFB+JAKSTAT+Notch+NFKB+Ccpos+Chemokine+Antiapop+Proapop, data=mgs5)

reg9 <- lm(Ang~PGDF+EGFR+Ilneg+Ilpos+IFN+Ccneg+mTOR+Hypoxia+AKT+IGF+PTEN+Bcell+Tcell+Antigen+Comp+Wnt+Hh+TGFB+JAKSTAT+Notch+NFKB+Ccpos+Chemokine+Antiapop+Proapop, data=mgs5)

reg10 <- lm(Hypoxia~Ang+PGDF+EGFR+Ilneg+Ilpos+IFN+Ccneg+mTOR+AKT+IGF+PTEN+Bcell+Tcell+Antigen+Comp+Wnt+Hh+TGFB+JAKSTAT+Notch+NFKB+Ccpos+Chemokine+Antiapop+Proapop, data=mgs5)

reg11 <- lm(AKT~Hypoxia+Ang+PGDF+EGFR+Ilneg+Ilpos+IFN+Ccneg+mTOR+IGF+PTEN+Bcell+Tcell+Antigen+Comp+Wnt+Hh+TGFB+JAKSTAT+Notch+NFKB+Ccpos+Chemokine+Antiapop+Proapop, data=mgs5)

reg12 <- lm(IGF~AKT+Hypoxia+Ang+PGDF+EGFR+Ilneg+Ilpos+IFN+Ccneg+mTOR+PTEN+Bcell+Tcell+Antigen+Comp+Wnt+Hh+TGFB+JAKSTAT+Notch+NFKB+Ccpos+Chemokine+Antiapop+Proapop, data=mgs5)

reg13 <- lm(PTEN~IGF+AKT+Hypoxia+Ang+PGDF+EGFR+Ilneg+Ilpos+IFN+Ccneg+mTOR+Bcell+Tcell+Antigen+Comp+Wnt+Hh+TGFB+JAKSTAT+Notch+NFKB+Ccpos+Chemokine+Antiapop+Proapop, data=mgs5)

reg14 <- lm(Bcell~PTEN+IGF+AKT+Hypoxia+Ang+PGDF+EGFR+Ilneg+Ilpos+IFN+Ccneg+mTOR+Tcell+Antigen+Comp+Wnt+Hh+TGFB+JAKSTAT+Notch+NFKB+Ccpos+Chemokine+Antiapop+Proapop, data=mgs5)

reg15 <- lm(Tcell~Bcell+PTEN+IGF+AKT+Hypoxia+Ang+PGDF+EGFR+Ilneg+Ilpos+IFN+Ccneg+mTOR+Antigen+Comp+Wnt+Hh+TGFB+JAKSTAT+Notch+NFKB+Ccpos+Chemokine+Antiapop+Proapop, data=mgs5)

reg16 <- lm(Antigen~Tcell+Bcell+PTEN+IGF+AKT+Hypoxia+Ang+PGDF+EGFR+Ilneg+Ilpos+IFN+Ccneg+mTOR+Comp+Wnt+Hh+TGFB+JAKSTAT+Notch+NFKB+Ccpos+Chemokine+Antiapop+Proapop, data=mgs5)

reg17 <- lm(Comp~Antigen+Tcell+Bcell+PTEN+IGF+AKT+Hypoxia+Ang+PGDF+EGFR+Ilneg+Ilpos+IFN+Ccneg+mTOR+Wnt+Hh+TGFB+JAKSTAT+Notch+NFKB+Ccpos+Chemokine+Antiapop+Proapop, data=mgs5)

reg18 <- lm(Wnt~Comp+Antigen+Tcell+Bcell+PTEN+IGF+AKT+Hypoxia+Ang+PGDF+EGFR+Ilneg+Ilpos+IFN+Ccneg+mTOR+Hh+TGFB+JAKSTAT+Notch+NFKB+Ccpos+Chemokine+Antiapop+Proapop, data=mgs5)

reg19 <- lm(Hh~Wnt+Comp+Antigen+Tcell+Bcell+PTEN+IGF+AKT+Hypoxia+Ang+PGDF+EGFR+Ilneg+Ilpos+IFN+Ccneg+mTOR+TGFB+JAKSTAT+Notch+NFKB+Ccpos+Chemokine+Antiapop+Proapop, data=mgs5)

reg20 <- lm(TGFB~Hh+Wnt+Comp+Antigen+Tcell+Bcell+PTEN+IGF+AKT+Hypoxia+Ang+PGDF+EGFR+Ilneg+Ilpos+IFN+Ccneg+mTOR+JAKSTAT+Notch+NFKB+Ccpos+Chemokine+Antiapop+Proapop, data=mgs5)

reg21 <- lm(JAKSTAT~TGFB+Hh+Wnt+Comp+Antigen+Tcell+Bcell+PTEN+IGF+AKT+Hypoxia+Ang+PGDF+EGFR+Ilneg+Ilpos+IFN+Ccneg+mTOR+Notch+NFKB+Ccpos+Chemokine+Antiapop+Proapop, data=mgs5)

reg22 <- lm(Notch~JAKSTAT+TGFB+Hh+Wnt+Comp+Antigen+Tcell+Bcell+PTEN+IGF+AKT+Hypoxia+Ang+PGDF+EGFR+Ilneg+Ilpos+IFN+Ccneg+mTOR+NFKB+Ccpos+Chemokine+Antiapop+Proapop, data=mgs5)

reg23 <- lm(NFKB~Notch+JAKSTAT+TGFB+Hh+Wnt+Comp+Antigen+Tcell+Bcell+PTEN+IGF+AKT+Hypoxia+Ang+PGDF+EGFR+Ilneg+Ilpos+IFN+Ccneg+mTOR+Ccpos+Chemokine+Antiapop+Proapop, data=mgs5)

reg24 <- lm(Ccpos~NFKB+Notch+JAKSTAT+TGFB+Hh+Wnt+Comp+Antigen+Tcell+Bcell+PTEN+IGF+AKT+Hypoxia+Ang+PGDF+EGFR+Ilneg+Ilpos+IFN+Ccneg+mTOR+Chemokine+Antiapop+Proapop, data=mgs5)

reg25 <- lm(Chemokine~Ccpos+NFKB+Notch+JAKSTAT+TGFB+Hh+Wnt+Comp+Antigen+Tcell+Bcell+PTEN+IGF+AKT+Hypoxia+Ang+PGDF+EGFR+Ilneg+Ilpos+IFN+Ccneg+mTOR+Antiapop+Proapop, data=mgs5)

reg26 <- lm(Antiapop~Chemokine+Ccpos+NFKB+Notch+JAKSTAT+TGFB+Hh+Wnt+Comp+Antigen+Tcell+Bcell+PTEN+IGF+AKT+Hypoxia+Ang+PGDF+EGFR+Ilneg+Ilpos+IFN+Ccneg+mTOR+Proapop, data=mgs5)

reg27 <- lm(Proapop~Antiapop+Chemokine+Ccpos+NFKB+Notch+JAKSTAT+TGFB+Hh+Wnt+Comp+Antigen+Tcell+Bcell+PTEN+IGF+AKT+Hypoxia+Ang+PGDF+EGFR+Ilneg+Ilpos+IFN+Ccneg+mTOR, data=mgs5)

# Removed Tcell

reg2 <- lm(mTOR~Ccneg+IFN+Ilpos+Ilneg+EGFR+PGDF+Ang+Hypoxia+AKT+IGF+PTEN+Bcell+Antigen+Comp+Wnt+Hh+TGFB+JAKSTAT+Notch+NFKB+Ccpos+Chemokine+Antiapop+Proapop, data=mgs5)

reg3 <- lm(Ccneg~mTOR+IFN+Ilpos+Ilneg+EGFR+PGDF+Ang+Hypoxia+AKT+IGF+PTEN+Bcell+Antigen+Comp+Wnt+Hh+TGFB+JAKSTAT+Notch+NFKB+Ccpos+Chemokine+Antiapop+Proapop, data=mgs5)

reg4 <- lm(IFN~Ccneg+mTOR+Ilpos+Ilneg+EGFR+PGDF+Ang+Hypoxia+AKT+IGF+PTEN+Bcell+Antigen+Comp+Wnt+Hh+TGFB+JAKSTAT+Notch+NFKB+Ccpos+Chemokine+Antiapop+Proapop, data=mgs5)

reg5 <- lm(Ilpos~IFN+Ccneg+mTOR+Ilneg+EGFR+PGDF+Ang+Hypoxia+AKT+IGF+PTEN+Bcell+Antigen+Comp+Wnt+Hh+TGFB+JAKSTAT+Notch+NFKB+Ccpos+Chemokine+Antiapop+Proapop, data=mgs5)

reg6 <- lm(Ilneg~Ilpos+IFN+Ccneg+mTOR+EGFR+PGDF+Ang+Hypoxia+AKT+IGF+PTEN+Bcell+Antigen+Comp+Wnt+Hh+TGFB+JAKSTAT+Notch+NFKB+Ccpos+Chemokine+Antiapop+Proapop, data=mgs5)

reg7 <- lm(EGFR~Ilneg+Ilpos+IFN+Ccneg+mTOR+PGDF+Ang+Hypoxia+AKT+IGF+PTEN+Bcell+Antigen+Comp+Wnt+Hh+TGFB+JAKSTAT+Notch+NFKB+Ccpos+Chemokine+Antiapop+Proapop, data=mgs5)

reg8 <- lm(PGDF~EGFR+Ilneg+Ilpos+IFN+Ccneg+mTOR+Ang+Hypoxia+AKT+IGF+PTEN+Bcell+Antigen+Comp+Wnt+Hh+TGFB+JAKSTAT+Notch+NFKB+Ccpos+Chemokine+Antiapop+Proapop, data=mgs5)

reg9 <- lm(Ang~PGDF+EGFR+Ilneg+Ilpos+IFN+Ccneg+mTOR+Hypoxia+AKT+IGF+PTEN+Bcell+Antigen+Comp+Wnt+Hh+TGFB+JAKSTAT+Notch+NFKB+Ccpos+Chemokine+Antiapop+Proapop, data=mgs5)

reg10 <- lm(Hypoxia~Ang+PGDF+EGFR+Ilneg+Ilpos+IFN+Ccneg+mTOR+AKT+IGF+PTEN+Bcell+Antigen+Comp+Wnt+Hh+TGFB+JAKSTAT+Notch+NFKB+Ccpos+Chemokine+Antiapop+Proapop, data=mgs5)

reg11 <- lm(AKT~Hypoxia+Ang+PGDF+EGFR+Ilneg+Ilpos+IFN+Ccneg+mTOR+IGF+PTEN+Bcell+Antigen+Comp+Wnt+Hh+TGFB+JAKSTAT+Notch+NFKB+Ccpos+Chemokine+Antiapop+Proapop, data=mgs5)

reg12 <- lm(IGF~AKT+Hypoxia+Ang+PGDF+EGFR+Ilneg+Ilpos+IFN+Ccneg+mTOR+PTEN+Bcell+Antigen+Comp+Wnt+Hh+TGFB+JAKSTAT+Notch+NFKB+Ccpos+Chemokine+Antiapop+Proapop, data=mgs5)

reg13 <- lm(PTEN~IGF+AKT+Hypoxia+Ang+PGDF+EGFR+Ilneg+Ilpos+IFN+Ccneg+mTOR+Bcell+Antigen+Comp+Wnt+Hh+TGFB+JAKSTAT+Notch+NFKB+Ccpos+Chemokine+Antiapop+Proapop, data=mgs5)

reg14 <- lm(Bcell~PTEN+IGF+AKT+Hypoxia+Ang+PGDF+EGFR+Ilneg+Ilpos+IFN+Ccneg+mTOR+Antigen+Comp+Wnt+Hh+TGFB+JAKSTAT+Notch+NFKB+Ccpos+Chemokine+Antiapop+Proapop, data=mgs5)

reg16 <- lm(Antigen~Bcell+PTEN+IGF+AKT+Hypoxia+Ang+PGDF+EGFR+Ilneg+Ilpos+IFN+Ccneg+mTOR+Comp+Wnt+Hh+TGFB+JAKSTAT+Notch+NFKB+Ccpos+Chemokine+Antiapop+Proapop, data=mgs5)

reg17 <- lm(Comp~Antigen+Bcell+PTEN+IGF+AKT+Hypoxia+Ang+PGDF+EGFR+Ilneg+Ilpos+IFN+Ccneg+mTOR+Wnt+Hh+TGFB+JAKSTAT+Notch+NFKB+Ccpos+Chemokine+Antiapop+Proapop, data=mgs5)

reg18 <- lm(Wnt~Comp+Antigen+Bcell+PTEN+IGF+AKT+Hypoxia+Ang+PGDF+EGFR+Ilneg+Ilpos+IFN+Ccneg+mTOR+Hh+TGFB+JAKSTAT+Notch+NFKB+Ccpos+Chemokine+Antiapop+Proapop, data=mgs5)

reg19 <- lm(Hh~Wnt+Comp+Antigen+Bcell+PTEN+IGF+AKT+Hypoxia+Ang+PGDF+EGFR+Ilneg+Ilpos+IFN+Ccneg+mTOR+TGFB+JAKSTAT+Notch+NFKB+Ccpos+Chemokine+Antiapop+Proapop, data=mgs5)

reg20 <- lm(TGFB~Hh+Wnt+Comp+Antigen+Bcell+PTEN+IGF+AKT+Hypoxia+Ang+PGDF+EGFR+Ilneg+Ilpos+IFN+Ccneg+mTOR+JAKSTAT+Notch+NFKB+Ccpos+Chemokine+Antiapop+Proapop, data=mgs5)

reg21 <- lm(JAKSTAT~TGFB+Hh+Wnt+Comp+Antigen+Bcell+PTEN+IGF+AKT+Hypoxia+Ang+PGDF+EGFR+Ilneg+Ilpos+IFN+Ccneg+mTOR+Notch+NFKB+Ccpos+Chemokine+Antiapop+Proapop, data=mgs5)

reg22 <- lm(Notch~JAKSTAT+TGFB+Hh+Wnt+Comp+Antigen+Bcell+PTEN+IGF+AKT+Hypoxia+Ang+PGDF+EGFR+Ilneg+Ilpos+IFN+Ccneg+mTOR+NFKB+Ccpos+Chemokine+Antiapop+Proapop, data=mgs5)

reg23 <- lm(NFKB~Notch+JAKSTAT+TGFB+Hh+Wnt+Comp+Antigen+Bcell+PTEN+IGF+AKT+Hypoxia+Ang+PGDF+EGFR+Ilneg+Ilpos+IFN+Ccneg+mTOR+Ccpos+Chemokine+Antiapop+Proapop, data=mgs5)

reg24 <- lm(Ccpos~NFKB+Notch+JAKSTAT+TGFB+Hh+Wnt+Comp+Antigen+Bcell+PTEN+IGF+AKT+Hypoxia+Ang+PGDF+EGFR+Ilneg+Ilpos+IFN+Ccneg+mTOR+Chemokine+Antiapop+Proapop, data=mgs5)

reg25 <- lm(Chemokine~Ccpos+NFKB+Notch+JAKSTAT+TGFB+Hh+Wnt+Comp+Antigen+Bcell+PTEN+IGF+AKT+Hypoxia+Ang+PGDF+EGFR+Ilneg+Ilpos+IFN+Ccneg+mTOR+Antiapop+Proapop, data=mgs5)

reg26 <- lm(Antiapop~Chemokine+Ccpos+NFKB+Notch+JAKSTAT+TGFB+Hh+Wnt+Comp+Antigen+Bcell+PTEN+IGF+AKT+Hypoxia+Ang+PGDF+EGFR+Ilneg+Ilpos+IFN+Ccneg+mTOR+Proapop, data=mgs5)

reg27 <- lm(Proapop~Antiapop+Chemokine+Ccpos+NFKB+Notch+JAKSTAT+TGFB+Hh+Wnt+Comp+Antigen+Bcell+PTEN+IGF+AKT+Hypoxia+Ang+PGDF+EGFR+Ilneg+Ilpos+IFN+Ccneg+mTOR, data=mgs5)

# Removed Chemokine

reg2 <- lm(mTOR~Ccneg+IFN+Ilpos+Ilneg+EGFR+PGDF+Ang+Hypoxia+AKT+IGF+PTEN+Bcell+Antigen+Comp+Wnt+Hh+TGFB+JAKSTAT+Notch+NFKB+Ccpos+Antiapop+Proapop, data=mgs5)

reg3 <- lm(Ccneg~mTOR+IFN+Ilpos+Ilneg+EGFR+PGDF+Ang+Hypoxia+AKT+IGF+PTEN+Bcell+Antigen+Comp+Wnt+Hh+TGFB+JAKSTAT+Notch+NFKB+Ccpos+Antiapop+Proapop, data=mgs5)

reg4 <- lm(IFN~Ccneg+mTOR+Ilpos+Ilneg+EGFR+PGDF+Ang+Hypoxia+AKT+IGF+PTEN+Bcell+Antigen+Comp+Wnt+Hh+TGFB+JAKSTAT+Notch+NFKB+Ccpos+Antiapop+Proapop, data=mgs5)

reg5 <- lm(Ilpos~IFN+Ccneg+mTOR+Ilneg+EGFR+PGDF+Ang+Hypoxia+AKT+IGF+PTEN+Bcell+Antigen+Comp+Wnt+Hh+TGFB+JAKSTAT+Notch+NFKB+Ccpos+Antiapop+Proapop, data=mgs5)

reg6 <- lm(Ilneg~Ilpos+IFN+Ccneg+mTOR+EGFR+PGDF+Ang+Hypoxia+AKT+IGF+PTEN+Bcell+Antigen+Comp+Wnt+Hh+TGFB+JAKSTAT+Notch+NFKB+Ccpos+Antiapop+Proapop, data=mgs5)

reg7 <- lm(EGFR~Ilneg+Ilpos+IFN+Ccneg+mTOR+PGDF+Ang+Hypoxia+AKT+IGF+PTEN+Bcell+Antigen+Comp+Wnt+Hh+TGFB+JAKSTAT+Notch+NFKB+Ccpos+Antiapop+Proapop, data=mgs5)

reg8 <- lm(PGDF~EGFR+Ilneg+Ilpos+IFN+Ccneg+mTOR+Ang+Hypoxia+AKT+IGF+PTEN+Bcell+Antigen+Comp+Wnt+Hh+TGFB+JAKSTAT+Notch+NFKB+Ccpos+Antiapop+Proapop, data=mgs5)

reg9 <- lm(Ang~PGDF+EGFR+Ilneg+Ilpos+IFN+Ccneg+mTOR+Hypoxia+AKT+IGF+PTEN+Bcell+Antigen+Comp+Wnt+Hh+TGFB+JAKSTAT+Notch+NFKB+Ccpos+Antiapop+Proapop, data=mgs5)

reg10 <- lm(Hypoxia~Ang+PGDF+EGFR+Ilneg+Ilpos+IFN+Ccneg+mTOR+AKT+IGF+PTEN+Bcell+Antigen+Comp+Wnt+Hh+TGFB+JAKSTAT+Notch+NFKB+Ccpos+Antiapop+Proapop, data=mgs5)

reg11 <- lm(AKT~Hypoxia+Ang+PGDF+EGFR+Ilneg+Ilpos+IFN+Ccneg+mTOR+IGF+PTEN+Bcell+Antigen+Comp+Wnt+Hh+TGFB+JAKSTAT+Notch+NFKB+Ccpos+Antiapop+Proapop, data=mgs5)

reg12 <- lm(IGF~AKT+Hypoxia+Ang+PGDF+EGFR+Ilneg+Ilpos+IFN+Ccneg+mTOR+PTEN+Bcell+Antigen+Comp+Wnt+Hh+TGFB+JAKSTAT+Notch+NFKB+Ccpos+Antiapop+Proapop, data=mgs5)

reg13 <- lm(PTEN~IGF+AKT+Hypoxia+Ang+PGDF+EGFR+Ilneg+Ilpos+IFN+Ccneg+mTOR+Bcell+Antigen+Comp+Wnt+Hh+TGFB+JAKSTAT+Notch+NFKB+Ccpos+Antiapop+Proapop, data=mgs5)

reg14 <- lm(Bcell~PTEN+IGF+AKT+Hypoxia+Ang+PGDF+EGFR+Ilneg+Ilpos+IFN+Ccneg+mTOR+Antigen+Comp+Wnt+Hh+TGFB+JAKSTAT+Notch+NFKB+Ccpos+Antiapop+Proapop, data=mgs5)

reg16 <- lm(Antigen~Bcell+PTEN+IGF+AKT+Hypoxia+Ang+PGDF+EGFR+Ilneg+Ilpos+IFN+Ccneg+mTOR+Comp+Wnt+Hh+TGFB+JAKSTAT+Notch+NFKB+Ccpos+Antiapop+Proapop, data=mgs5)

reg17 <- lm(Comp~Antigen+Bcell+PTEN+IGF+AKT+Hypoxia+Ang+PGDF+EGFR+Ilneg+Ilpos+IFN+Ccneg+mTOR+Wnt+Hh+TGFB+JAKSTAT+Notch+NFKB+Ccpos+Antiapop+Proapop, data=mgs5)

reg18 <- lm(Wnt~Comp+Antigen+Bcell+PTEN+IGF+AKT+Hypoxia+Ang+PGDF+EGFR+Ilneg+Ilpos+IFN+Ccneg+mTOR+Hh+TGFB+JAKSTAT+Notch+NFKB+Ccpos+Antiapop+Proapop, data=mgs5)

reg19 <- lm(Hh~Wnt+Comp+Antigen+Bcell+PTEN+IGF+AKT+Hypoxia+Ang+PGDF+EGFR+Ilneg+Ilpos+IFN+Ccneg+mTOR+TGFB+JAKSTAT+Notch+NFKB+Ccpos+Antiapop+Proapop, data=mgs5)

reg20 <- lm(TGFB~Hh+Wnt+Comp+Antigen+Bcell+PTEN+IGF+AKT+Hypoxia+Ang+PGDF+EGFR+Ilneg+Ilpos+IFN+Ccneg+mTOR+JAKSTAT+Notch+NFKB+Ccpos+Antiapop+Proapop, data=mgs5)

reg21 <- lm(JAKSTAT~TGFB+Hh+Wnt+Comp+Antigen+Bcell+PTEN+IGF+AKT+Hypoxia+Ang+PGDF+EGFR+Ilneg+Ilpos+IFN+Ccneg+mTOR+Notch+NFKB+Ccpos+Antiapop+Proapop, data=mgs5)

reg22 <- lm(Notch~JAKSTAT+TGFB+Hh+Wnt+Comp+Antigen+Bcell+PTEN+IGF+AKT+Hypoxia+Ang+PGDF+EGFR+Ilneg+Ilpos+IFN+Ccneg+mTOR+NFKB+Ccpos+Antiapop+Proapop, data=mgs5)

reg23 <- lm(NFKB~Notch+JAKSTAT+TGFB+Hh+Wnt+Comp+Antigen+Bcell+PTEN+IGF+AKT+Hypoxia+Ang+PGDF+EGFR+Ilneg+Ilpos+IFN+Ccneg+mTOR+Ccpos+Antiapop+Proapop, data=mgs5)

reg24 <- lm(Ccpos~NFKB+Notch+JAKSTAT+TGFB+Hh+Wnt+Comp+Antigen+Bcell+PTEN+IGF+AKT+Hypoxia+Ang+PGDF+EGFR+Ilneg+Ilpos+IFN+Ccneg+mTOR+Antiapop+Proapop, data=mgs5)

reg26 <- lm(Antiapop~Ccpos+NFKB+Notch+JAKSTAT+TGFB+Hh+Wnt+Comp+Antigen+Bcell+PTEN+IGF+AKT+Hypoxia+Ang+PGDF+EGFR+Ilneg+Ilpos+IFN+Ccneg+mTOR+Proapop, data=mgs5)

reg27 <- lm(Proapop~Antiapop+Ccpos+NFKB+Notch+JAKSTAT+TGFB+Hh+Wnt+Comp+Antigen+Bcell+PTEN+IGF+AKT+Hypoxia+Ang+PGDF+EGFR+Ilneg+Ilpos+IFN+Ccneg+mTOR, data=mgs5)

# Removed NFKB

reg2 <- lm(mTOR~Ccneg+IFN+Ilpos+Ilneg+EGFR+PGDF+Ang+Hypoxia+AKT+IGF+PTEN+Bcell+Antigen+Comp+Wnt+Hh+TGFB+JAKSTAT+Notch+Ccpos+Antiapop+Proapop, data=mgs5)

reg3 <- lm(Ccneg~mTOR+IFN+Ilpos+Ilneg+EGFR+PGDF+Ang+Hypoxia+AKT+IGF+PTEN+Bcell+Antigen+Comp+Wnt+Hh+TGFB+JAKSTAT+Notch+Ccpos+Antiapop+Proapop, data=mgs5)

reg4 <- lm(IFN~Ccneg+mTOR+Ilpos+Ilneg+EGFR+PGDF+Ang+Hypoxia+AKT+IGF+PTEN+Bcell+Antigen+Comp+Wnt+Hh+TGFB+JAKSTAT+Notch+Ccpos+Antiapop+Proapop, data=mgs5)

reg5 <- lm(Ilpos~IFN+Ccneg+mTOR+Ilneg+EGFR+PGDF+Ang+Hypoxia+AKT+IGF+PTEN+Bcell+Antigen+Comp+Wnt+Hh+TGFB+JAKSTAT+Notch+Ccpos+Antiapop+Proapop, data=mgs5)

reg6 <- lm(Ilneg~Ilpos+IFN+Ccneg+mTOR+EGFR+PGDF+Ang+Hypoxia+AKT+IGF+PTEN+Bcell+Antigen+Comp+Wnt+Hh+TGFB+JAKSTAT+Notch+Ccpos+Antiapop+Proapop, data=mgs5)

reg7 <- lm(EGFR~Ilneg+Ilpos+IFN+Ccneg+mTOR+PGDF+Ang+Hypoxia+AKT+IGF+PTEN+Bcell+Antigen+Comp+Wnt+Hh+TGFB+JAKSTAT+Notch+Ccpos+Antiapop+Proapop, data=mgs5)

reg8 <- lm(PGDF~EGFR+Ilneg+Ilpos+IFN+Ccneg+mTOR+Ang+Hypoxia+AKT+IGF+PTEN+Bcell+Antigen+Comp+Wnt+Hh+TGFB+JAKSTAT+Notch+Ccpos+Antiapop+Proapop, data=mgs5)

reg9 <- lm(Ang~PGDF+EGFR+Ilneg+Ilpos+IFN+Ccneg+mTOR+Hypoxia+AKT+IGF+PTEN+Bcell+Antigen+Comp+Wnt+Hh+TGFB+JAKSTAT+Notch+Ccpos+Antiapop+Proapop, data=mgs5)

reg10 <- lm(Hypoxia~Ang+PGDF+EGFR+Ilneg+Ilpos+IFN+Ccneg+mTOR+AKT+IGF+PTEN+Bcell+Antigen+Comp+Wnt+Hh+TGFB+JAKSTAT+Notch+Ccpos+Antiapop+Proapop, data=mgs5)

reg11 <- lm(AKT~Hypoxia+Ang+PGDF+EGFR+Ilneg+Ilpos+IFN+Ccneg+mTOR+IGF+PTEN+Bcell+Antigen+Comp+Wnt+Hh+TGFB+JAKSTAT+Notch+Ccpos+Antiapop+Proapop, data=mgs5)

reg12 <- lm(IGF~AKT+Hypoxia+Ang+PGDF+EGFR+Ilneg+Ilpos+IFN+Ccneg+mTOR+PTEN+Bcell+Antigen+Comp+Wnt+Hh+TGFB+JAKSTAT+Notch+Ccpos+Antiapop+Proapop, data=mgs5)

reg13 <- lm(PTEN~IGF+AKT+Hypoxia+Ang+PGDF+EGFR+Ilneg+Ilpos+IFN+Ccneg+mTOR+Bcell+Antigen+Comp+Wnt+Hh+TGFB+JAKSTAT+Notch+Ccpos+Antiapop+Proapop, data=mgs5)

reg14 <- lm(Bcell~PTEN+IGF+AKT+Hypoxia+Ang+PGDF+EGFR+Ilneg+Ilpos+IFN+Ccneg+mTOR+Antigen+Comp+Wnt+Hh+TGFB+JAKSTAT+Notch+Ccpos+Antiapop+Proapop, data=mgs5)

reg16 <- lm(Antigen~Bcell+PTEN+IGF+AKT+Hypoxia+Ang+PGDF+EGFR+Ilneg+Ilpos+IFN+Ccneg+mTOR+Comp+Wnt+Hh+TGFB+JAKSTAT+Notch+Ccpos+Antiapop+Proapop, data=mgs5)

reg17 <- lm(Comp~Antigen+Bcell+PTEN+IGF+AKT+Hypoxia+Ang+PGDF+EGFR+Ilneg+Ilpos+IFN+Ccneg+mTOR+Wnt+Hh+TGFB+JAKSTAT+Notch+Ccpos+Antiapop+Proapop, data=mgs5)

reg18 <- lm(Wnt~Comp+Antigen+Bcell+PTEN+IGF+AKT+Hypoxia+Ang+PGDF+EGFR+Ilneg+Ilpos+IFN+Ccneg+mTOR+Hh+TGFB+JAKSTAT+Notch+Ccpos+Antiapop+Proapop, data=mgs5)

reg19 <- lm(Hh~Wnt+Comp+Antigen+Bcell+PTEN+IGF+AKT+Hypoxia+Ang+PGDF+EGFR+Ilneg+Ilpos+IFN+Ccneg+mTOR+TGFB+JAKSTAT+Notch+Ccpos+Antiapop+Proapop, data=mgs5)

reg20 <- lm(TGFB~Hh+Wnt+Comp+Antigen+Bcell+PTEN+IGF+AKT+Hypoxia+Ang+PGDF+EGFR+Ilneg+Ilpos+IFN+Ccneg+mTOR+JAKSTAT+Notch+Ccpos+Antiapop+Proapop, data=mgs5)

reg21 <- lm(JAKSTAT~TGFB+Hh+Wnt+Comp+Antigen+Bcell+PTEN+IGF+AKT+Hypoxia+Ang+PGDF+EGFR+Ilneg+Ilpos+IFN+Ccneg+mTOR+Notch+Ccpos+Antiapop+Proapop, data=mgs5)

reg22 <- lm(Notch~JAKSTAT+TGFB+Hh+Wnt+Comp+Antigen+Bcell+PTEN+IGF+AKT+Hypoxia+Ang+PGDF+EGFR+Ilneg+Ilpos+IFN+Ccneg+mTOR+Ccpos+Antiapop+Proapop, data=mgs5)

reg24 <- lm(Ccpos~Notch+JAKSTAT+TGFB+Hh+Wnt+Comp+Antigen+Bcell+PTEN+IGF+AKT+Hypoxia+Ang+PGDF+EGFR+Ilneg+Ilpos+IFN+Ccneg+mTOR+Antiapop+Proapop, data=mgs5)

reg26 <- lm(Antiapop~Ccpos+Notch+JAKSTAT+TGFB+Hh+Wnt+Comp+Antigen+Bcell+PTEN+IGF+AKT+Hypoxia+Ang+PGDF+EGFR+Ilneg+Ilpos+IFN+Ccneg+mTOR+Proapop, data=mgs5)

reg27 <- lm(Proapop~Antiapop+Ccpos+Notch+JAKSTAT+TGFB+Hh+Wnt+Comp+Antigen+Bcell+PTEN+IGF+AKT+Hypoxia+Ang+PGDF+EGFR+Ilneg+Ilpos+IFN+Ccneg+mTOR, data=mgs5)

######################### Cluster associations including subtype #########################

g <- glm((cluster==1)~Proapop+Antiapop+Ccpos+Notch+JAKSTAT+TGFB+Hh+Wnt+Comp+Antigen+Bcell+PTEN+IGF+AKT+Hypoxia+Ang+PGDF+EGFR+Ilneg+Ilpos+IFN+Ccneg+mTOR+stage+grade+age+male+subtype, data=mgs3, family=binomial)

g1 <- step(g, direction="both")

h <- glm((cluster==2)~Proapop+Antiapop+Ccpos+Notch+JAKSTAT+TGFB+Hh+Wnt+Comp+Antigen+Bcell+PTEN+IGF+AKT+Hypoxia+Ang+PGDF+EGFR+Ilneg+Ilpos+IFN+Ccneg+mTOR+stage+grade+age+male+subtype, data=mgs3, family=binomial)

h1 <- step(h, direction="both")

i <- glm((cluster==3)~Proapop+Antiapop+Ccpos+Notch+JAKSTAT+TGFB+Hh+Wnt+Comp+Antigen+Bcell+PTEN+IGF+AKT+Hypoxia+Ang+PGDF+EGFR+Ilneg+Ilpos+IFN+Ccneg+mTOR+stage+grade+age+male+subtype, data=mgs3, family=binomial)

i1 <- step(i, direction="both")

######################### Cluster associations excluding subtype #########################

j <- glm((cluster==1)~Proapop+Antiapop+Ccpos+Notch+JAKSTAT+TGFB+Hh+Wnt+Comp+Antigen+Bcell+PTEN+IGF+AKT+Hypoxia+Ang+PGDF+EGFR+Ilneg+Ilpos+IFN+Ccneg+mTOR+stage+grade+age+male, data=mgs2, family=binomial)

j1 <- step(j, direction="both")

k <- glm((cluster==2)~Proapop+Antiapop+Ccpos+Notch+JAKSTAT+TGFB+Hh+Wnt+Comp+Antigen+Bcell+PTEN+IGF+AKT+Hypoxia+Ang+PGDF+EGFR+Ilneg+Ilpos+IFN+Ccneg+mTOR+stage+grade+age+male, data=mgs2, family=binomial)

k1 <- step(k, direction="both")

l <- glm((cluster==3)~Proapop+Antiapop+Ccpos+Notch+JAKSTAT+TGFB+Hh+Wnt+Comp+Antigen+Bcell+PTEN+IGF+AKT+Hypoxia+Ang+PGDF+EGFR+Ilneg+Ilpos+IFN+Ccneg+mTOR+stage+grade+age+male, data=mgs4, family=binomial)

l1 <- step(l, direction="both")

######################### Survival model adjusting for stage and clinical, w/ interactions #########################

sviv2 <- coxph(Surv(months5, dead5)~stage*Proapop+stage*Antiapop+stage*Ccpos+stage*Notch+stage*JAKSTAT+stage*TGFB+stage*Hh+stage*Wnt+stage*Comp+stage*Antigen+stage*Bcell+stage*PTEN+stage*IGF+stage*AKT+stage*Hypoxia+stage*Ang+stage*PGDF+stage*EGFR+stage*Ilneg+stage*Ilpos+stage*IFN+stage*Ccneg+stage*mTOR+grade+age+male+as.factor(site==3), data=mgs4)

sviv3 <- step(sviv2, direction="both")

######################### Survival model adjusting for stage and clinical, w/ interactions and with major subtype #########################

sviv4 <- coxph(Surv(months5,dead5)~stage*Proapop+stage*Antiapop+stage*Ccpos+stage*Notch+stage*JAKSTAT+stage*TGFB+stage*Hh+stage*Wnt+stage*Comp+stage*Antigen+stage*Bcell+stage*PTEN+stage*IGF+stage*AKT+stage*Hypoxia+stage*Ang+stage*PGDF+stage*EGFR+stage*Ilneg+stage*Ilpos+stage*IFN+stage*Ccneg+stage*mTOR+grade+age+male+subtype, data=mgs3)

sviv5 <- step(sviv4, direction="both")

######################### Survival model adjusting for stage and clinical, w/ interactions and with percent subtype #########################

sviv6 <- coxph(Surv(months5,dead5)~stage*Proapop+stage*Antiapop+stage*Ccpos+stage*Notch+stage*JAKSTAT+stage*TGFB+stage*Hh+stage*Wnt+stage*Comp+stage*Antigen+stage*Bcell+stage*PTEN+stage*IGF+stage*AKT+stage*Hypoxia+stage*Ang+stage*PGDF+stage*EGFR+stage*Ilneg+stage*Ilpos+stage*IFN+stage*Ccneg+stage*mTOR+grade+age+male+solidness+bacness+papness+acinarness, data=mgs3)

sviv7 <- step(sviv6, direction="both")

######################### Models for presence of each subtype #########################

# BAC

a <- glm((bacness>0)~Proapop+Antiapop+Ccpos+Notch+JAKSTAT+TGFB+Hh+Wnt+Comp+Antigen+Bcell+PTEN+IGF+AKT+Hypoxia+Ang+PGDF+EGFR+Ilneg+Ilpos+IFN+Ccneg+mTOR, data=mgs3, family=binomial)

a1 <- step(a, direction="both")

# Papillary

b <- glm((papness>0)~Proapop+Antiapop+Ccpos+Notch+JAKSTAT+TGFB+Hh+Wnt+Comp+Antigen+Bcell+PTEN+IGF+AKT+Hypoxia+Ang+PGDF+EGFR+Ilneg+Ilpos+IFN+Ccneg+mTOR, data=mgs3, family=binomial)

b1 <- step(b, direction="both")

#Acinar

c <- glm((acinarness>0)~Proapop+Antiapop+Ccpos+Notch+JAKSTAT+TGFB+Hh+Wnt+Comp+Antigen+Bcell+PTEN+IGF+AKT+Hypoxia+Ang+PGDF+EGFR+Ilneg+Ilpos+IFN+Ccneg+mTOR, data=mgs3, family=binomial)

c1 <- step(c, direction="both")

# Solid

d <- glm((solidness>0)~Proapop+Antiapop+Ccpos+Notch+JAKSTAT+TGFB+Hh+Wnt+Comp+Antigen+Bcell+PTEN+IGF+AKT+Hypoxia+Ang+PGDF+EGFR+Ilneg+Ilpos+IFN+Ccneg+mTOR, data=mgs3, family=binomial)

d1 <- step(d, direction="both")

######################### Percent solidness/bacness/papillaryness/acinarness and associated Pathways #########################

s1 <- lm(solidness~Proapop+Antiapop+Ccpos+Notch+JAKSTAT+TGFB+Hh+Wnt+Comp+Antigen+Bcell+PTEN+IGF+AKT+Hypoxia+Ang+PGDF+EGFR+Ilneg+Ilpos+IFN+Ccneg+mTOR, data=mgs3)

s2 <- step(s1, direction="both")

b1 <- lm(bacness~Proapop+Antiapop+Ccpos+Notch+JAKSTAT+TGFB+Hh+Wnt+Comp+Antigen+Bcell+PTEN+IGF+AKT+Hypoxia+Ang+PGDF+EGFR+Ilneg+Ilpos+IFN+Ccneg+mTOR, data=mgs3)

b2 <- step(b1, direction="both")

p1 <- lm(papness~Proapop+Antiapop+Ccpos+Notch+JAKSTAT+TGFB+Hh+Wnt+Comp+Antigen+Bcell+PTEN+IGF+AKT+Hypoxia+Ang+PGDF+EGFR+Ilneg+Ilpos+IFN+Ccneg+mTOR, data=mgs3)

p2 <- step(p1, direction="both")

a1 <- lm(acinarness~Proapop+Antiapop+Ccpos+Notch+JAKSTAT+TGFB+Hh+Wnt+Comp+Antigen+Bcell+PTEN+IGF+AKT+Hypoxia+Ang+PGDF+EGFR+Ilneg+Ilpos+IFN+Ccneg+mTOR, data=mgs3)

a2 <- step(a1, direction="both")

##Kaplan-Meier curves

data <- read.csv(file="C:/All Pathways and clinical.csv", header=TRUE, row.names=1)

library(survival)

colnames(data)

stage1 <- subset(data, stage==1)

cluster12 <- subset(data, cluster<3)

cluster13 <- subset(data, (cluster==1)|(cluster==3))

cluster23 <- subset(data, (cluster==2)|(cluster==3))

gradelow <- subset(data, grade==0)

stage2 <- subset(data, stage==2)

stage3 <- subset(data, stage==3)

anysolid <- subset(data, solidness>0)

Ccposup <- subset(data, Ccpos>0)

survdiff(Surv(months5,dead5)~(solidness>0), data=data)

plot(survfit(Surv(months5,dead5)~(solidness>0), data=data), xlab="Months", ylab="Survival", xlim=c(0,60), ylim=c(0,1), col=c(3,2))

title("Kaplan-Meier Survival Curve - Solid Component", font.main=1)

legend(locator(1), c("w/o Solid", "w/ Solid"), lty=1, bty="n", col=c(3,2))

legend("bottomleft", c(" p=0.000166"), bty="n")

survdiff(Surv(months5,dead5)~(solidness), data=data)

plot(survfit(Surv(months5,dead5)~solidness, data=data), xlab="Months", ylab="Survival", xlim=c(0,60), ylim=c(0,1), col=c(1,2,3,4,5,6,7,8,9,10,11,12))

title("Kaplan-Meier Survival Curve - Solid Component", font.main=1)

legend(locator(1), c("0%", "5%", "10%", "20%", "30%", "40%", "50%", "60%", "70%", "80%", "90%", "100%"), lty=1, bty="n", col=c(1,2,3,4,5,6,7,8,9,10,11,12))

legend("bottomleft", c(" p=0.00118"), bty="n")

survdiff(Surv(months5,dead5)~(acinarness>0), data=data)

plot(survfit(Surv(months5,dead5)~(acinarness>0), data=data), xlab="Months", ylab="Survival", xlim=c(0,60), ylim=c(0,1), col=c(2,3))

title("Kaplan-Meier Survival Curve - Acinar Component", font.main=1)

legend(locator(1), c("w/o Acinar", "w/ Acinar"), lty=1, bty="n", col=c(2,3))

legend("bottomleft", c(" p=0.898"), bty="n")

survdiff(Surv(months5,dead5)~(papness>0), data=data)

plot(survfit(Surv(months5,dead5)~(papness>0), data=data), xlab="Months", ylab="Survival", xlim=c(0,60), ylim=c(0,1), col=c(2,3))

title("Kaplan-Meier Survival Curve - Papillary Component", font.main=1)

legend(locator(1), c("w/o Papillary", "w/ Papillary"), lty=1, bty="n", col=c(2,3))

legend("bottomleft", c(" p=0.119"), bty="n")

survdiff(Surv(months5,dead5)~(bacness>0), data=data)

plot(survfit(Surv(months5,dead5)~(bacness>0), data=data), xlab="Months", ylab="Survival", xlim=c(0,60), ylim=c(0,1), col=c(2,3))

title("Kaplan-Meier Survival Curve - BAC Component", font.main=1)

legend(locator(1), c("w/o BAC", "w/ BAC"), lty=1, bty="n", col=c(2,3))

legend("bottomleft", c(" p=0.0342"), bty="n")

survdiff(Surv(months5,dead5)~(solidness>0), data=stage1)

plot(survfit(Surv(months5,dead5)~(solidness>0), data=stage1), xlab="Months", ylab="Survival", xlim=c(0,60), ylim=c(0,1), col=c(3,2))

title("Kaplan-Meier Survival Curve - Solid Component in Stage 1", font.main=1)

legend(locator(1), c("w/o Solid", "w/ Solid"), lty=1, bty="n", col=c(3,2))

legend("bottomleft", c(" p=0.00562"), bty="n")

survdiff(Surv(months5,dead5)~(acinarness>0), data=stage1)

plot(survfit(Surv(months5,dead5)~(acinarness>0), data=stage1), xlab="Months", ylab="Survival", xlim=c(0,60), ylim=c(0,1), col=c(2,3))

title("Kaplan-Meier Survival Curve - Acinar Component in Stage 1", font.main=1)

legend(locator(1), c("w/o Acinar", "w/ Acinar"), lty=1, bty="n", col=c(2,3))

legend("bottomleft", c(" p=0.391"), bty="n")

survdiff(Surv(months5,dead5)~(papness>0), data=stage1)

plot(survfit(Surv(months5,dead5)~(papness>0), data=stage1), xlab="Months", ylab="Survival", xlim=c(0,60), ylim=c(0,1), col=c(2,3))

title("Kaplan-Meier Survival Curve - Papillary Component in Stage 1", font.main=1)

legend(locator(1), c("w/o Papillary", "w/ Papillary"), lty=1, bty="n", col=c(2,3))

legend("bottomleft", c(" p=0.571"), bty="n")

survdiff(Surv(months5,dead5)~(bacness>0), data=stage1)

plot(survfit(Surv(months5,dead5)~(bacness>0), data=stage1), xlab="Months", ylab="Survival", xlim=c(0,60), ylim=c(0,1), col=c(2,3))

title("Kaplan-Meier Survival Curve - BAC Component in Stage 1", font.main=1)

legend(locator(1), c("w/o BAC", "w/ BAC"), lty=1, bty="n", col=c(2,3))

legend("bottomleft", c(" p=0.232"), bty="n")

survdiff(Surv(months5,dead5)~(solidness>0), data=stage2)

plot(survfit(Surv(months5,dead5)~(solidness>0), data=stage2), xlab="Months", ylab="Survival", xlim=c(0,60), ylim=c(0,1), col=c(3,2))

title("Kaplan-Meier Survival Curve - Solid Component in Stage 2", font.main=1)

legend(locator(1), c("w/o Solid", "w/ Solid"), lty=1, bty="n", col=c(3,2))

legend("bottomleft", c(" p=0.35"), bty="n")

survdiff(Surv(months5,dead5)~(acinarness>0), data=stage2)

plot(survfit(Surv(months5,dead5)~(acinarness>0), data=stage2), xlab="Months", ylab="Survival", xlim=c(0,60), ylim=c(0,1), col=c(2,3))

title("Kaplan-Meier Survival Curve - Acinar Component in Stage 2", font.main=1)

legend(locator(1), c("w/o Acinar", "w/ Acinar"), lty=1, bty="n", col=c(2,3))

legend("bottomleft", c(" p=0.906"), bty="n")

survdiff(Surv(months5,dead5)~(papness>0), data=stage2)

plot(survfit(Surv(months5,dead5)~(papness>0), data=stage2), xlab="Months", ylab="Survival", xlim=c(0,60), ylim=c(0,1), col=c(2,3))

title("Kaplan-Meier Survival Curve - Papillary Component in Stage 2", font.main=1)

legend(locator(1), c("w/o Papillary", "w/ Papillary"), lty=1, bty="n", col=c(2,3))

legend("bottomleft", c(" p=0.741"), bty="n")

survdiff(Surv(months5,dead5)~(bacness>0), data=stage2)

plot(survfit(Surv(months5,dead5)~(bacness>0), data=stage2), xlab="Months", ylab="Survival", xlim=c(0,60), ylim=c(0,1), col=c(2,3))

title("Kaplan-Meier Survival Curve - BAC Component in Stage 2", font.main=1)

legend(locator(1), c("w/o BAC", "w/ BAC"), lty=1, bty="n", col=c(2,3))

legend("bottomleft", c(" p=0.618"), bty="n")

survdiff(Surv(months5,dead5)~(solidness>0), data=stage3)

plot(survfit(Surv(months5,dead5)~(solidness>0), data=stage3), xlab="Months", ylab="Survival", xlim=c(0,60), ylim=c(0,1), col=c(3,2))

title("Kaplan-Meier Survival Curve - Solid Component in Stage 3", font.main=1)

legend(locator(1), c("w/o Solid", "w/ Solid"), lty=1, bty="n", col=c(3,2))

legend("bottomleft", c(" p=0.0295"), bty="n")

survdiff(Surv(months5,dead5)~(acinarness>0), data=stage3)

plot(survfit(Surv(months5,dead5)~(acinarness>0), data=stage3), xlab="Months", ylab="Survival", xlim=c(0,60), ylim=c(0,1), col=c(2,3))

title("Kaplan-Meier Survival Curve - Acinar Component in Stage 3", font.main=1)

legend(locator(1), c("w/ Acinar", "w/o Acinar"), lty=1, bty="n", col=c(3,2))

legend("bottomleft", c(" p=0.225"), bty="n")

survdiff(Surv(months5,dead5)~(papness>0), data=stage3)

plot(survfit(Surv(months5,dead5)~(papness>0), data=stage3), xlab="Months", ylab="Survival", xlim=c(0,60), ylim=c(0,1), col=c(2,3))

title("Kaplan-Meier Survival Curve - Papillary Component in Stage 3", font.main=1)

legend(locator(1), c("w/o Papillary", "w/ Papillary"), lty=1, bty="n", col=c(2,3))

legend("bottomleft", c(" p=0.0113"), bty="n")

survdiff(Surv(months5,dead5)~(bacness>0), data=stage3)

plot(survfit(Surv(months5,dead5)~(bacness>0), data=stage3), xlab="Months", ylab="Survival", xlim=c(0,60), ylim=c(0,1), col=c(2,3))

title("Kaplan-Meier Survival Curve - BAC Component in Stage 3", font.main=1)

legend(locator(1), c("w/o BAC", "w/ BAC"), lty=1, bty="n", col=c(2,3))

legend("bottomleft", c(" p=0.302"), bty="n")

survdiff(Surv(months5,dead5)~(Ccpos>0), data=data)

plot(survfit(Surv(months5,dead5)~(Ccpos>0), data=data), xlab="Months", ylab="Survival", xlim=c(0,60), ylim=c(0,1), col=c(3,2))

title("Kaplan-Meier Survival Curve - Cell Cycle Pathway", font.main=1)

legend(locator(1), c("Cell Cycle>0", "Cell Cycle<0"), lty=1, bty="n", col=c(2,3))

legend("bottomleft", c(" p=0.000113"), bty="n")

survdiff(Surv(months5,dead5)~(ESC>0), data=data)

plot(survfit(Surv(months5,dead5)~(ESC>0), data=data), xlab="Months", ylab="Survival", xlim=c(0,60), ylim=c(0,1), col=c(3,2))

title("Kaplan-Meier Survival Curve - ESC Pathway", font.main=1)

legend(locator(1), c("ESC>0", "ESC<0"), lty=1, bty="n", col=c(2,3))

legend("bottomleft", c(" p=0.0155"), bty="n")

survdiff(Surv(months5,dead5)~(Ilneg>0), data=data)

plot(survfit(Surv(months5,dead5)~(Ilneg>0), data=data), xlab="Months", ylab="Survival", xlim=c(0,60), ylim=c(0,1), col=c(3,2))

title("Kaplan-Meier Survival Curve - Immunosuppressive IL", font.main=1)

legend(locator(1), c("Ilneg>0", "Ilneg<0"), lty=1, bty="n", col=c(2,3))

legend("bottomleft", c(" p=0.0309"), bty="n")

survdiff(Surv(months5,dead5)~cluster, data=data)

plot(survfit(Surv(months5,dead5)~cluster, data=data), xlab="Months", ylab="Survival", xlim=c(0,60), ylim=c(0,1), col=c(4,3,2))

title("Kaplan-Meier Survival Curve - Clusters", font.main=1)

legend(locator(1), c("Cluster 1", "Cluster 2", "Cluster 3"), lty=1, bty="n", col=c(4,3,2))

legend("bottomleft", c(" p=0.000194"), bty="n")

survdiff(Surv(months,dead)~(cluster==1), data=data)

plot(survfit(Surv(months5,dead5)~(cluster==1), data=data), xlab="Months", ylab="Survival", xlim=c(0,60), ylim=c(0,1), col=c(2,3))

title("Kaplan-Meier Survival Curve - Cluster 1", font.main=1)

legend(locator(1), c("Cluster 1", "Not Cluster 1"), lty=1, bty="n", col=c(2,3))

legend("bottomleft", c(" p=0.00422"), bty="n")

survdiff(Surv(months5,dead5)~(cluster==2), data=data)

plot(survfit(Surv(months5,dead5)~(cluster==2), data=data), xlab="Months", ylab="Survival", xlim=c(0,60), ylim=c(0,1), col=c(2,3))

title("Kaplan-Meier Survival Curve - Cluster 2", font.main=1)

legend(locator(1), c("Cluster 2", "Not Cluster 2"), lty=1, bty="n", col=c(2,3))

legend("bottomleft", c(" p=0.217"), bty="n")

survdiff(Surv(months5,dead5)~(cluster==3), data=data)

plot(survfit(Surv(months5,dead5)~(cluster==3), data=data), xlab="Months", ylab="Survival", xlim=c(0,60), ylim=c(0,1), col=c(3,2))

title("Kaplan-Meier Survival Curve - Cluster 3", font.main=1)

legend(locator(1), c("Cluster 3", "Not Cluster 3"), lty=1, bty="n", col=c(2,3))

legend("bottomleft", c(" p=0.0000504"), bty="n")

cluster12 <- subset(data, cluster<3)

survdiff(Surv(months5,dead5)~(cluster==1), data=cluster12)

plot(survfit(Surv(months5,dead5)~(cluster==1), data=cluster12), xlab="Months", ylab="Survival", xlim=c(0,60), ylim=c(0,1), col=c(2,3))

title("Kaplan-Meier Survival Curve - Cluster 1 v. Cluster 2", font.main=1)

legend(locator(1), c("Cluster 1", "Cluster 2"), lty=1, bty="n", col=c(2,3))

legend("bottomleft", c(" p=0.347"), bty="n")

cluster13 <- subset(data, (cluster==1)|(cluster==3))

survdiff(Surv(months5,dead5)~(cluster==1), data=cluster13)

plot(survfit(Surv(months5,dead5)~(cluster==1), data=cluster13), xlab="Months", ylab="Survival", xlim=c(0,60), ylim=c(0,1), col=c(2,3))

title("Kaplan-Meier Survival Curve - Cluster 1 v. Cluster 3", font.main=1)

legend(locator(1), c("Cluster 1", "Cluster 3"), lty=1, bty="n", col=c(2,3))

legend("bottomleft", c(" p=0.000122"), bty="n")

cluster23 <- subset(data, (cluster==2)|(cluster==3))

survdiff(Surv(months5,dead5)~(cluster==2), data=cluster23)

plot(survfit(Surv(months5,dead5)~(cluster==2), data=cluster23), xlab="Months", ylab="Survival", xlim=c(0,60), ylim=c(0,1), col=c(2,3))

title("Kaplan-Meier Survival Curve - Cluster 2 v. Cluster 3", font.main=1)

legend(locator(1), c("Cluster 2", "Cluster 3"), lty=1, bty="n", col=c(3,2))

legend("bottomleft", c(" p=0.00582"), bty="n")

survdiff(Surv(months5,dead5)~(stage), data=data)

plot(survfit(Surv(months5,dead5)~(stage), data=data), xlab="Months", ylab="Survival", xlim=c(0,60), ylim=c(0,1), col=c(4,3,2))

title("Kaplan-Meier Survival Curve - Stage", font.main=1)

legend(locator(1), c("Stage 1", "Stage 2", "Stage 3"), lty=1, bty="n", col=c(4,3,2))

legend("bottomleft", c(" p<0.000001"), bty="n")

survdiff(Surv(months5,dead5)~(Ccpos>0), data=stage1)

plot(survfit(Surv(months5,dead5)~(Ccpos>0), data=stage1), xlab="Months", ylab="Survival", xlim=c(0,60), ylim=c(0,1), col=c(3,2))

title("Kaplan-Meier Survival Curve - Cell Cycle Pathway in Stage 1", font.main=1)

legend(locator(1), c("Cell Cycle>0", "Cell Cycle<0"), lty=1, bty="n", col=c(2,3))

legend("bottomleft", c(" p=0.062"), bty="n")

survdiff(Surv(months5,dead5)~(ESC>0), data=stage1)

plot(survfit(Surv(months5,dead5)~(ESC>0), data=stage1), xlab="Months", ylab="Survival", xlim=c(0,60), ylim=c(0,1), col=c(3,2))

title("Kaplan-Meier Survival Curve - ESC Pathway in Stage 1", font.main=1)

legend(locator(1), c("ESC>0", "ESC<0"), lty=1, bty="n", col=c(2,3))

legend("bottomleft", c(" p=0.305"), bty="n")

survdiff(Surv(months5,dead5)~(Ilneg>0), data=stage1)

plot(survfit(Surv(months5,dead5)~(Ilneg>0), data=stage1), xlab="Months", ylab="Survival", xlim=c(0,60), ylim=c(0,1), col=c(3,2))

title("Kaplan-Meier Survival Curve - Immunosuppressive IL in Stage 1", font.main=1)

legend(locator(1), c("Ilneg>0", "Ilneg<0"), lty=1, bty="n", col=c(2,3))

legend("bottomleft", c(" p=0.33"), bty="n")

survdiff(Surv(months5,dead5)~(Ilneg>0 & Ccpos>0 & ESC>0), data=data)

plot(survfit(Surv(months5,dead5)~(Ilneg>0), data=data), xlab="Months", ylab="Survival", xlim=c(0,60), ylim=c(0,1), col=c(3,2))

title("Kaplan-Meier Survival Curve - Cell Cycle, ESC and Ilneg", font.main=1)

legend(locator(1), c("All overexpressed", "Not all overexpressed"), lty=1, bty="n", col=c(2,3))

legend("bottomleft", c(" p=0.00152"), bty="n")

survdiff(Surv(months5,dead5)~(Ilneg>0 & Ccpos>0 & ESC>0), data=stage1)

plot(survfit(Surv(months5,dead5)~(Ilneg>0), data=stage1), xlab="Months", ylab="Survival", xlim=c(0,60), ylim=c(0,1), col=c(3,2))

title("Kaplan-Meier Survival Curve - Cell Cycle, ESC and Ilneg in Stage 1", font.main=1)

legend(locator(1), c("All overexpressed", "Not all overexpressed"), lty=1, bty="n", col=c(2,3))

legend("bottomleft", c(" p=0.165"), bty="n")

survdiff(Surv(months5,dead5)~(grade), data=data)

plot(survfit(Surv(months5,dead5)~(grade), data=data), xlab="Months", ylab="Survival", xlim=c(0,60), ylim=c(0,1), col=c(3,2))

title("Kaplan-Meier Survival Curve - Grade", font.main=1)

legend(locator(1), c("Low and Moderate Grade", "High Grade"), lty=1, bty="n", col=c(3,2))

legend("bottomleft", c(" p=0.0245"), bty="n")

survdiff(Surv(months5,dead5)~(solidness>0), data=gradelow)

plot(survfit(Surv(months5,dead5)~(solidness>0), data=gradelow), xlab="Months", ylab="Survival", xlim=c(0,60), ylim=c(0,1), col=c(3,2))

title("Kaplan-Meier Survival Curve - Solid Component in Low Grade", font.main=1)

legend(locator(1), c("w/o Solid", "w/ Solid"), lty=1, bty="n", col=c(3,2))

legend("bottomleft", c(" p=0.00112"), bty="n")

survdiff(Surv(months5,dead5)~(Ccpos>0), data=gradelow)

plot(survfit(Surv(months5,dead5)~(Ccpos>0), data=gradelow), xlab="Months", ylab="Survival", xlim=c(0,60), ylim=c(0,1), col=c(3,2))

title("Kaplan-Meier Survival Curve - Cell Cycle Pathway in Low Grade", font.main=1)

legend(locator(1), c("Cell Cycle>0", "Cell Cycle<0"), lty=1, bty="n", col=c(2,3))

legend("bottomleft", c(" p=0.0121"), bty="n")

survdiff(Surv(months5,dead5)~(Ccpos>0), data=stage2)

plot(survfit(Surv(months5,dead5)~(Ccpos>0), data=stage2), xlab="Months", ylab="Survival", xlim=c(0,60), ylim=c(0,1), col=c(3,2))

title("Kaplan-Meier Survival Curve - Cell Cycle Pathway in Stage 2", font.main=1)

legend(locator(1), c("Cell Cycle>0", "Cell Cycle<0"), lty=1, bty="n", col=c(3,2))

legend("bottomleft", c(" p=0.697"), bty="n")

survdiff(Surv(months5,dead5)~(Ccpos>0), data=stage3)

plot(survfit(Surv(months5,dead5)~(Ccpos>0), data=stage3), xlab="Months", ylab="Survival", xlim=c(0,60), ylim=c(0,1), col=c(3,2))

title("Kaplan-Meier Survival Curve - Cell Cycle Pathway in Stage 3", font.main=1)

legend(locator(1), c("Cell Cycle>0", "Cell Cycle<0"), lty=1, bty="n", col=c(2,3))

legend("bottomleft", c(" p=0.0042"), bty="n")

survdiff(Surv(months5,dead5)~(Ccpos>0), data=stage23)

plot(survfit(Surv(months5,dead5)~(Ccpos>0), data=stage23), xlab="Months", ylab="Survival", xlim=c(0,60), ylim=c(0,1), col=c(3,2))

title("Kaplan-Meier Survival Curve - Cell Cycle Pathway in Stage 2 and 3", font.main=1)

legend(locator(1), c("Cell Cycle>0", "Cell Cycle<0"), lty=1, bty="n", col=c(2,3))

legend("bottomleft", c(" p=0.0449"), bty="n")

survdiff(Surv(months5,dead5)~(male==0), data=data)

plot(survfit(Surv(months5,dead5)~(male==0), data=data), xlab="Months", ylab="Survival", xlim=c(0,60), ylim=c(0,1), col=c(2,3))

title("Kaplan-Meier Survival Curve - Sex", font.main=1)

legend(locator(1), c("Female", "Male"), lty=1, bty="n", col=c(3,2))

legend("bottomleft", c(" p=0.0429"), bty="n")

survdiff(Surv(months5,dead5)~(subtype), data=data)

plot(survfit(Surv(months5,dead5)~(subtype), data=data), xlab="Months", ylab="Survival", xlim=c(0,60), ylim=c(0,1), col=c(2,3,4,5,6))

title("Kaplan-Meier Survival Curve - Major Subtype", font.main=1)

legend(locator(1), c("Other", "BAC", "Papillary", "Acinar", "Solid"), lty=1, bty="n", col=c(2,3,4,5,6))

legend("bottomleft", c(" p=0.113"), bty="n")

survdiff(Surv(months5,dead5)~(subtype==4), data=data)

plot(survfit(Surv(months5,dead5)~(subtype==4), data=data), xlab="Months", ylab="Survival", xlim=c(0,60), ylim=c(0,1), col=c(3,2))

title("Kaplan-Meier Survival Curve - Solid Major Subtype", font.main=1)

legend(locator(1), c("Solid", "All others"), lty=1, bty="n", col=c(2,3,4,5,6))

legend("bottomleft", c(" p=0.015"), bty="n")

survdiff(Surv(months5,dead5)~(Ccpos>0), data=anysolid)

plot(survfit(Surv(months5,dead5)~(Ccpos>0), data=anysolid), xlab="Months", ylab="Survival", xlim=c(0,60), ylim=c(0,1), col=c(3,2))

title("Kaplan-Meier Survival Curve - Cell Cycle in Solid Tumors", font.main=1)

legend(locator(1), c("Cell Cycle<0", "Cell Cycle>0"), lty=1, bty="n", col=c(3,2))

legend("bottomleft", c(" p=0.331"), bty="n")

survdiff(Surv(months5,dead5)~(solidness>0), data=Ccposup)

plot(survfit(Surv(months5,dead5)~(solidness>0), data=Ccposup), xlab="Months", ylab="Survival", xlim=c(0,60), ylim=c(0,1), col=c(3,2))

title("Kaplan-Meier Survival Curve - Solidness in Cell Cycle Positive Tumors", font.main=1)

legend(locator(1), c("w/o Solid", "w/ Solid"), lty=1, bty="n", col=c(3,2))

legend("bottomleft", c(" p=0.0995"), bty="n")

##gene enrichment

generich <- read.csv(file="C:/HR and Meta PS.csv", header=TRUE)

colnames(generich)

allprobes <- generich[,1]

cluster1upHR <- generich[,2]

cluster1downHR <- generich[,3]

cluster2upHR <- generich[,4]

cluster2downHR <- generich[,5]

cluster3upHR <- generich[,6]

cluster3downHR <- generich[,7]

intersect(allprobes, cluster1upHR)

NROW(intersect(allprobes, cluster1upHR))

##1747=n

intersect(allprobes, cluster2upHR)

NROW(intersect(allprobes, cluster2upHR))

##1957=n

intersect(allprobes, cluster3upHR)

NROW(intersect(allprobes, cluster3upHR))

##1224=n

intersect(generich[,X], clusterXupHR)

##NROW(intersect(generich[,X], clusterXupHR))-2

##=q

##subtract 2 because it gives an extra matched term and for pvalue

NROW(allprobes)

##16660-q=k

NROW(intersect(generich[,X], generich[,X]))-1

##=m

##subtract 1 because it gives extra matched term

##gene enrichment for cell cycle (+) Pathway

phyper((NROW(intersect(generich[,23], cluster1upHR))-2), (NROW(intersect(generich[,23], generich[,23]))-1), (NROW(allprobes)-(NROW(intersect(generich[,23], generich[,23]))-1)), (NROW(intersect(allprobes, cluster1upHR))), lower.tail=FALSE)

phyper((NROW(intersect(generich[,23], cluster2upHR))-2), (NROW(intersect(generich[,23], generich[,23]))-1), (NROW(allprobes)-(NROW(intersect(generich[,23], generich[,23]))-1)), (NROW(intersect(allprobes, cluster2upHR))), lower.tail=FALSE)

phyper((NROW(intersect(generich[,23], cluster3upHR))-2), (NROW(intersect(generich[,23], generich[,23]))-1), (NROW(allprobes)-(NROW(intersect(generich[,23], generich[,23]))-1)), (NROW(intersect(allprobes, cluster3upHR))), lower.tail=FALSE)

phyper((NROW(intersect(generich[,23], cluster1downHR))-2), (NROW(intersect(generich[,23], generich[,23]))-1), (NROW(allprobes)-(NROW(intersect(generich[,23], generich[,23]))-1)), (NROW(intersect(allprobes, cluster1downHR))), lower.tail=FALSE)

phyper((NROW(intersect(generich[,23], cluster2downHR))-2), (NROW(intersect(generich[,23], generich[,23]))-1), (NROW(allprobes)-(NROW(intersect(generich[,23], generich[,23]))-1)), (NROW(intersect(allprobes, cluster2downHR))), lower.tail=FALSE)

phyper((NROW(intersect(generich[,23], cluster3downHR))-2), (NROW(intersect(generich[,23], generich[,23]))-1), (NROW(allprobes)-(NROW(intersect(generich[,23], generich[,23]))-1)), (NROW(intersect(allprobes, cluster3downHR))), lower.tail=FALSE)

##gene enrichment for mTOR Pathway

phyper((NROW(intersect(generich[,8], cluster1upHR))-2), (NROW(intersect(generich[,8], generich[,8]))-1), (NROW(allprobes)-(NROW(intersect(generich[,8], generich[,8]))-1)), (NROW(intersect(allprobes, cluster1upHR))), lower.tail=FALSE)

phyper((NROW(intersect(generich[,8], cluster2upHR))-2), (NROW(intersect(generich[,8], generich[,8]))-1), (NROW(allprobes)-(NROW(intersect(generich[,8], generich[,8]))-1)), (NROW(intersect(allprobes, cluster2upHR))), lower.tail=FALSE)

phyper((NROW(intersect(generich[,8], cluster3upHR))-2), (NROW(intersect(generich[,8], generich[,8]))-1), (NROW(allprobes)-(NROW(intersect(generich[,8], generich[,8]))-1)), (NROW(intersect(allprobes, cluster3upHR))), lower.tail=FALSE)

phyper((NROW(intersect(generich[,8], cluster1downHR))-2), (NROW(intersect(generich[,8], generich[,8]))-1), (NROW(allprobes)-(NROW(intersect(generich[,8], generich[,8]))-1)), (NROW(intersect(allprobes, cluster1downHR))), lower.tail=FALSE)

phyper((NROW(intersect(generich[,8], cluster2downHR))-2), (NROW(intersect(generich[,8], generich[,8]))-1), (NROW(allprobes)-(NROW(intersect(generich[,8], generich[,8]))-1)), (NROW(intersect(allprobes, cluster2downHR))), lower.tail=FALSE)

phyper((NROW(intersect(generich[,8], cluster3downHR))-2), (NROW(intersect(generich[,8], generich[,8]))-1), (NROW(allprobes)-(NROW(intersect(generich[,8], generich[,8]))-1)), (NROW(intersect(allprobes, cluster3downHR))), lower.tail=FALSE)

##gene enrichment for cell cycle (-) Pathway

phyper((NROW(intersect(generich[,9], cluster1upHR))-2), (NROW(intersect(generich[,9], generich[,9]))-1), (NROW(allprobes)-(NROW(intersect(generich[,9], generich[,9]))-1)), (NROW(intersect(allprobes, cluster1upHR))), lower.tail=FALSE)

phyper((NROW(intersect(generich[,9], cluster2upHR))-2), (NROW(intersect(generich[,9], generich[,9]))-1), (NROW(allprobes)-(NROW(intersect(generich[,9], generich[,9]))-1)), (NROW(intersect(allprobes, cluster2upHR))), lower.tail=FALSE)

phyper((NROW(intersect(generich[,9], cluster3upHR))-2), (NROW(intersect(generich[,9], generich[,9]))-1), (NROW(allprobes)-(NROW(intersect(generich[,9], generich[,9]))-1)), (NROW(intersect(allprobes, cluster3upHR))), lower.tail=FALSE)

phyper((NROW(intersect(generich[,9], cluster1downHR))-2), (NROW(intersect(generich[,9], generich[,9]))-1), (NROW(allprobes)-(NROW(intersect(generich[,9], generich[,9]))-1)), (NROW(intersect(allprobes, cluster1downHR))), lower.tail=FALSE)

phyper((NROW(intersect(generich[,9], cluster2downHR))-2), (NROW(intersect(generich[,9], generich[,9]))-1), (NROW(allprobes)-(NROW(intersect(generich[,9], generich[,9]))-1)), (NROW(intersect(allprobes, cluster2downHR))), lower.tail=FALSE)

phyper((NROW(intersect(generich[,9], cluster3downHR))-2), (NROW(intersect(generich[,9], generich[,9]))-1), (NROW(allprobes)-(NROW(intersect(generich[,9], generich[,9]))-1)), (NROW(intersect(allprobes, cluster3downHR))), lower.tail=FALSE)

##gene enrichment for angiogenesis Pathway

phyper((NROW(intersect(generich[,15], cluster1upHR))-2), (NROW(intersect(generich[,15], generich[,15]))-1), (NROW(allprobes)-(NROW(intersect(generich[,15], generich[,15]))-1)), (NROW(intersect(allprobes, cluster1upHR))), lower.tail=FALSE)

phyper((NROW(intersect(generich[,15], cluster2upHR))-2), (NROW(intersect(generich[,15], generich[,15]))-1), (NROW(allprobes)-(NROW(intersect(generich[,15], generich[,15]))-1)), (NROW(intersect(allprobes, cluster2upHR))), lower.tail=FALSE)

phyper((NROW(intersect(generich[,15], cluster3upHR))-2), (NROW(intersect(generich[,15], generich[,15]))-1), (NROW(allprobes)-(NROW(intersect(generich[,15], generich[,15]))-1)), (NROW(intersect(allprobes, cluster3upHR))), lower.tail=FALSE)

phyper((NROW(intersect(generich[,15], cluster1downHR))-2), (NROW(intersect(generich[,15], generich[,15]))-1), (NROW(allprobes)-(NROW(intersect(generich[,15], generich[,15]))-1)), (NROW(intersect(allprobes, cluster1downHR))), lower.tail=FALSE)

phyper((NROW(intersect(generich[,15], cluster2downHR))-2), (NROW(intersect(generich[,15], generich[,15]))-1), (NROW(allprobes)-(NROW(intersect(generich[,15], generich[,15]))-1)), (NROW(intersect(allprobes, cluster2downHR))), lower.tail=FALSE)

phyper((NROW(intersect(generich[,15], cluster3downHR))-2), (NROW(intersect(generich[,15], generich[,15]))-1), (NROW(allprobes)-(NROW(intersect(generich[,15], generich[,15]))-1)), (NROW(intersect(allprobes, cluster3downHR))), lower.tail=FALSE)

##gene enrichment for Ilpos Pathway

phyper((NROW(intersect(generich[,11], cluster1upHR))-2), (NROW(intersect(generich[,11], generich[,11]))-1), (NROW(allprobes)-(NROW(intersect(generich[,11], generich[,11]))-1)), (NROW(intersect(allprobes, cluster1upHR))), lower.tail=FALSE)

phyper((NROW(intersect(generich[,11], cluster2upHR))-2), (NROW(intersect(generich[,11], generich[,11]))-1), (NROW(allprobes)-(NROW(intersect(generich[,11], generich[,11]))-1)), (NROW(intersect(allprobes, cluster2upHR))), lower.tail=FALSE)

phyper((NROW(intersect(generich[,11], cluster3upHR))-2), (NROW(intersect(generich[,11], generich[,11]))-1), (NROW(allprobes)-(NROW(intersect(generich[,11], generich[,11]))-1)), (NROW(intersect(allprobes, cluster3upHR))), lower.tail=FALSE)

phyper((NROW(intersect(generich[,11], cluster1downHR))-2), (NROW(intersect(generich[,11], generich[,11]))-1), (NROW(allprobes)-(NROW(intersect(generich[,11], generich[,11]))-1)), (NROW(intersect(allprobes, cluster1downHR))), lower.tail=FALSE)

phyper((NROW(intersect(generich[,11], cluster2downHR))-2), (NROW(intersect(generich[,11], generich[,11]))-1), (NROW(allprobes)-(NROW(intersect(generich[,11], generich[,11]))-1)), (NROW(intersect(allprobes, cluster2downHR))), lower.tail=FALSE)

phyper((NROW(intersect(generich[,11], cluster3downHR))-2), (NROW(intersect(generich[,11], generich[,11]))-1), (NROW(allprobes)-(NROW(intersect(generich[,11], generich[,11]))-1)), (NROW(intersect(allprobes, cluster3downHR))), lower.tail=FALSE)

##gene enrichment for Ilneg Pathway

phyper((NROW(intersect(generich[,12], cluster1upHR))-2), (NROW(intersect(generich[,12], generich[,12]))-1), (NROW(allprobes)-(NROW(intersect(generich[,12], generich[,12]))-1)), (NROW(intersect(allprobes, cluster1upHR))), lower.tail=FALSE)

phyper((NROW(intersect(generich[,12], cluster2upHR))-2), (NROW(intersect(generich[,12], generich[,12]))-1), (NROW(allprobes)-(NROW(intersect(generich[,12], generich[,12]))-1)), (NROW(intersect(allprobes, cluster2upHR))), lower.tail=FALSE)

phyper((NROW(intersect(generich[,12], cluster3upHR))-2), (NROW(intersect(generich[,12], generich[,12]))-1), (NROW(allprobes)-(NROW(intersect(generich[,12], generich[,12]))-1)), (NROW(intersect(allprobes, cluster3upHR))), lower.tail=FALSE)

phyper((NROW(intersect(generich[,12], cluster1downHR))-2), (NROW(intersect(generich[,12], generich[,12]))-1), (NROW(allprobes)-(NROW(intersect(generich[,12], generich[,12]))-1)), (NROW(intersect(allprobes, cluster1downHR))), lower.tail=FALSE)

phyper((NROW(intersect(generich[,12], cluster2downHR))-2), (NROW(intersect(generich[,12], generich[,12]))-1), (NROW(allprobes)-(NROW(intersect(generich[,12], generich[,12]))-1)), (NROW(intersect(allprobes, cluster2downHR))), lower.tail=FALSE)

phyper((NROW(intersect(generich[,12], cluster3downHR))-2), (NROW(intersect(generich[,12], generich[,12]))-1), (NROW(allprobes)-(NROW(intersect(generich[,12], generich[,12]))-1)), (NROW(intersect(allprobes, cluster3downHR))), lower.tail=FALSE)

##gene enrichment for Bcell Pathway

phyper((NROW(intersect(generich[,31], cluster1upHR))-2), (NROW(intersect(generich[,31], generich[,31]))-1), (NROW(allprobes)-(NROW(intersect(generich[,31], generich[,31]))-1)), (NROW(intersect(allprobes, cluster1upHR))), lower.tail=FALSE)

phyper((NROW(intersect(generich[,31], cluster2upHR))-2), (NROW(intersect(generich[,31], generich[,31]))-1), (NROW(allprobes)-(NROW(intersect(generich[,31], generich[,31]))-1)), (NROW(intersect(allprobes, cluster2upHR))), lower.tail=FALSE)

phyper((NROW(intersect(generich[,31], cluster3upHR))-2), (NROW(intersect(generich[,31], generich[,31]))-1), (NROW(allprobes)-(NROW(intersect(generich[,31], generich[,31]))-1)), (NROW(intersect(allprobes, cluster3upHR))), lower.tail=FALSE)

phyper((NROW(intersect(generich[,31], cluster1downHR))-2), (NROW(intersect(generich[,31], generich[,31]))-1), (NROW(allprobes)-(NROW(intersect(generich[,31], generich[,31]))-1)), (NROW(intersect(allprobes, cluster1downHR))), lower.tail=FALSE)

phyper((NROW(intersect(generich[,31], cluster2downHR))-2), (NROW(intersect(generich[,31], generich[,31]))-1), (NROW(allprobes)-(NROW(intersect(generich[,31], generich[,31]))-1)), (NROW(intersect(allprobes, cluster2downHR))), lower.tail=FALSE)

phyper((NROW(intersect(generich[,31], cluster3downHR))-2), (NROW(intersect(generich[,31], generich[,31]))-1), (NROW(allprobes)-(NROW(intersect(generich[,31], generich[,31]))-1)), (NROW(intersect(allprobes, cluster3downHR))), lower.tail=FALSE)

##gene enrichment for Tcell Pathway

phyper((NROW(intersect(generich[,32], cluster1upHR))-2), (NROW(intersect(generich[,32], generich[,32]))-1), (NROW(allprobes)-(NROW(intersect(generich[,32], generich[,32]))-1)), (NROW(intersect(allprobes, cluster1upHR))), lower.tail=FALSE)

phyper((NROW(intersect(generich[,32], cluster2upHR))-2), (NROW(intersect(generich[,32], generich[,32]))-1), (NROW(allprobes)-(NROW(intersect(generich[,32], generich[,32]))-1)), (NROW(intersect(allprobes, cluster2upHR))), lower.tail=FALSE)

phyper((NROW(intersect(generich[,32], cluster3upHR))-2), (NROW(intersect(generich[,32], generich[,32]))-1), (NROW(allprobes)-(NROW(intersect(generich[,32], generich[,32]))-1)), (NROW(intersect(allprobes, cluster3upHR))), lower.tail=FALSE)

phyper((NROW(intersect(generich[,32], cluster1downHR))-2), (NROW(intersect(generich[,32], generich[,32]))-1), (NROW(allprobes)-(NROW(intersect(generich[,32], generich[,32]))-1)), (NROW(intersect(allprobes, cluster1downHR))), lower.tail=FALSE)

phyper((NROW(intersect(generich[,32], cluster2downHR))-2), (NROW(intersect(generich[,32], generich[,32]))-1), (NROW(allprobes)-(NROW(intersect(generich[,32], generich[,32]))-1)), (NROW(intersect(allprobes, cluster2downHR))), lower.tail=FALSE)

phyper((NROW(intersect(generich[,32], cluster3downHR))-2), (NROW(intersect(generich[,32], generich[,32]))-1), (NROW(allprobes)-(NROW(intersect(generich[,32], generich[,32]))-1)), (NROW(intersect(allprobes, cluster3downHR))), lower.tail=FALSE)

##gene enrichment for Antigen Pathway

phyper((NROW(intersect(generich[,33], cluster1upHR))-2), (NROW(intersect(generich[,33], generich[,33]))-1), (NROW(allprobes)-(NROW(intersect(generich[,33], generich[,33]))-1)), (NROW(intersect(allprobes, cluster1upHR))), lower.tail=FALSE)

phyper((NROW(intersect(generich[,33], cluster2upHR))-2), (NROW(intersect(generich[,33], generich[,33]))-1), (NROW(allprobes)-(NROW(intersect(generich[,33], generich[,33]))-1)), (NROW(intersect(allprobes, cluster2upHR))), lower.tail=FALSE)

phyper((NROW(intersect(generich[,33], cluster3upHR))-2), (NROW(intersect(generich[,33], generich[,33]))-1), (NROW(allprobes)-(NROW(intersect(generich[,33], generich[,33]))-1)), (NROW(intersect(allprobes, cluster3upHR))), lower.tail=FALSE)

phyper((NROW(intersect(generich[,33], cluster1downHR))-2), (NROW(intersect(generich[,33], generich[,33]))-1), (NROW(allprobes)-(NROW(intersect(generich[,33], generich[,33]))-1)), (NROW(intersect(allprobes, cluster1downHR))), lower.tail=FALSE)

phyper((NROW(intersect(generich[,33], cluster2downHR))-2), (NROW(intersect(generich[,33], generich[,33]))-1), (NROW(allprobes)-(NROW(intersect(generich[,33], generich[,33]))-1)), (NROW(intersect(allprobes, cluster2downHR))), lower.tail=FALSE)

phyper((NROW(intersect(generich[,33], cluster3downHR))-2), (NROW(intersect(generich[,33], generich[,33]))-1), (NROW(allprobes)-(NROW(intersect(generich[,33], generich[,33]))-1)), (NROW(intersect(allprobes, cluster3downHR))), lower.tail=FALSE)

##gene enrichment for complement Pathway

phyper((NROW(intersect(generich[,34], cluster1upHR))-2), (NROW(intersect(generich[,34], generich[,34]))-1), (NROW(allprobes)-(NROW(intersect(generich[,34], generich[,34]))-1)), (NROW(intersect(allprobes, cluster1upHR))), lower.tail=FALSE)

phyper((NROW(intersect(generich[,34], cluster2upHR))-2), (NROW(intersect(generich[,34], generich[,34]))-1), (NROW(allprobes)-(NROW(intersect(generich[,34], generich[,34]))-1)), (NROW(intersect(allprobes, cluster2upHR))), lower.tail=FALSE)

phyper((NROW(intersect(generich[,34], cluster3upHR))-2), (NROW(intersect(generich[,34], generich[,34]))-1), (NROW(allprobes)-(NROW(intersect(generich[,34], generich[,34]))-1)), (NROW(intersect(allprobes, cluster3upHR))), lower.tail=FALSE)

phyper((NROW(intersect(generich[,34], cluster1downHR))-2), (NROW(intersect(generich[,34], generich[,34]))-1), (NROW(allprobes)-(NROW(intersect(generich[,34], generich[,34]))-1)), (NROW(intersect(allprobes, cluster1downHR))), lower.tail=FALSE)

phyper((NROW(intersect(generich[,34], cluster2downHR))-2), (NROW(intersect(generich[,34], generich[,34]))-1), (NROW(allprobes)-(NROW(intersect(generich[,34], generich[,34]))-1)), (NROW(intersect(allprobes, cluster2downHR))), lower.tail=FALSE)

phyper((NROW(intersect(generich[,34], cluster3downHR))-2), (NROW(intersect(generich[,34], generich[,34]))-1), (NROW(allprobes)-(NROW(intersect(generich[,34], generich[,34]))-1)), (NROW(intersect(allprobes, cluster3downHR))), lower.tail=FALSE)

##gene enrichment for interferon Pathway

phyper((NROW(intersect(generich[,10], cluster1upHR))-2), (NROW(intersect(generich[,10], generich[,10]))-1), (NROW(allprobes)-(NROW(intersect(generich[,10], generich[,10]))-1)), (NROW(intersect(allprobes, cluster1upHR))), lower.tail=FALSE)

phyper((NROW(intersect(generich[,10], cluster2upHR))-2), (NROW(intersect(generich[,10], generich[,10]))-1), (NROW(allprobes)-(NROW(intersect(generich[,10], generich[,10]))-1)), (NROW(intersect(allprobes, cluster2upHR))), lower.tail=FALSE)

phyper((NROW(intersect(generich[,10], cluster3upHR))-2), (NROW(intersect(generich[,10], generich[,10]))-1), (NROW(allprobes)-(NROW(intersect(generich[,10], generich[,10]))-1)), (NROW(intersect(allprobes, cluster3upHR))), lower.tail=FALSE)

phyper((NROW(intersect(generich[,10], cluster1downHR))-2), (NROW(intersect(generich[,10], generich[,10]))-1), (NROW(allprobes)-(NROW(intersect(generich[,10], generich[,10]))-1)), (NROW(intersect(allprobes, cluster1downHR))), lower.tail=FALSE)

phyper((NROW(intersect(generich[,10], cluster2downHR))-2), (NROW(intersect(generich[,10], generich[,10]))-1), (NROW(allprobes)-(NROW(intersect(generich[,10], generich[,10]))-1)), (NROW(intersect(allprobes, cluster2downHR))), lower.tail=FALSE)

phyper((NROW(intersect(generich[,10], cluster3downHR))-2), (NROW(intersect(generich[,10], generich[,10]))-1), (NROW(allprobes)-(NROW(intersect(generich[,10], generich[,10]))-1)), (NROW(intersect(allprobes, cluster3downHR))), lower.tail=FALSE)

##gene enrichment for EGFR Pathway

phyper((NROW(intersect(generich[,13], cluster1upHR))-2), (NROW(intersect(generich[,13], generich[,13]))-1), (NROW(allprobes)-(NROW(intersect(generich[,13], generich[,13]))-1)), (NROW(intersect(allprobes, cluster1upHR))), lower.tail=FALSE)

phyper((NROW(intersect(generich[,13], cluster2upHR))-2), (NROW(intersect(generich[,13], generich[,13]))-1), (NROW(allprobes)-(NROW(intersect(generich[,13], generich[,13]))-1)), (NROW(intersect(allprobes, cluster2upHR))), lower.tail=FALSE)

phyper((NROW(intersect(generich[,13], cluster3upHR))-2), (NROW(intersect(generich[,13], generich[,13]))-1), (NROW(allprobes)-(NROW(intersect(generich[,13], generich[,13]))-1)), (NROW(intersect(allprobes, cluster3upHR))), lower.tail=FALSE)

phyper((NROW(intersect(generich[,13], cluster1downHR))-2), (NROW(intersect(generich[,13], generich[,13]))-1), (NROW(allprobes)-(NROW(intersect(generich[,13], generich[,13]))-1)), (NROW(intersect(allprobes, cluster1downHR))), lower.tail=FALSE)

phyper((NROW(intersect(generich[,13], cluster2downHR))-2), (NROW(intersect(generich[,13], generich[,13]))-1), (NROW(allprobes)-(NROW(intersect(generich[,13], generich[,13]))-1)), (NROW(intersect(allprobes, cluster2downHR))), lower.tail=FALSE)

phyper((NROW(intersect(generich[,13], cluster3downHR))-2), (NROW(intersect(generich[,13], generich[,13]))-1), (NROW(allprobes)-(NROW(intersect(generich[,13], generich[,13]))-1)), (NROW(intersect(allprobes, cluster3downHR))), lower.tail=FALSE)

##gene enrichment for PDGF Pathway

phyper((NROW(intersect(generich[,14], cluster1upHR))-2), (NROW(intersect(generich[,14], generich[,14]))-1), (NROW(allprobes)-(NROW(intersect(generich[,14], generich[,14]))-1)), (NROW(intersect(allprobes, cluster1upHR))), lower.tail=FALSE)

phyper((NROW(intersect(generich[,14], cluster2upHR))-2), (NROW(intersect(generich[,14], generich[,14]))-1), (NROW(allprobes)-(NROW(intersect(generich[,14], generich[,14]))-1)), (NROW(intersect(allprobes, cluster2upHR))), lower.tail=FALSE)

phyper((NROW(intersect(generich[,14], cluster3upHR))-2), (NROW(intersect(generich[,14], generich[,14]))-1), (NROW(allprobes)-(NROW(intersect(generich[,14], generich[,14]))-1)), (NROW(intersect(allprobes, cluster3upHR))), lower.tail=FALSE)

phyper((NROW(intersect(generich[,14], cluster1downHR))-2), (NROW(intersect(generich[,14], generich[,14]))-1), (NROW(allprobes)-(NROW(intersect(generich[,14], generich[,14]))-1)), (NROW(intersect(allprobes, cluster1downHR))), lower.tail=FALSE)

phyper((NROW(intersect(generich[,14], cluster2downHR))-2), (NROW(intersect(generich[,14], generich[,14]))-1), (NROW(allprobes)-(NROW(intersect(generich[,14], generich[,14]))-1)), (NROW(intersect(allprobes, cluster2downHR))), lower.tail=FALSE)

phyper((NROW(intersect(generich[,14], cluster3downHR))-2), (NROW(intersect(generich[,14], generich[,14]))-1), (NROW(allprobes)-(NROW(intersect(generich[,14], generich[,14]))-1)), (NROW(intersect(allprobes, cluster3downHR))), lower.tail=FALSE)

##gene enrichment for hypoxia Pathway

phyper((NROW(intersect(generich[,16], cluster1upHR))-2), (NROW(intersect(generich[,16], generich[,16]))-1), (NROW(allprobes)-(NROW(intersect(generich[,16], generich[,16]))-1)), (NROW(intersect(allprobes, cluster1upHR))), lower.tail=FALSE)

phyper((NROW(intersect(generich[,16], cluster2upHR))-2), (NROW(intersect(generich[,16], generich[,16]))-1), (NROW(allprobes)-(NROW(intersect(generich[,16], generich[,16]))-1)), (NROW(intersect(allprobes, cluster2upHR))), lower.tail=FALSE)

phyper((NROW(intersect(generich[,16], cluster3upHR))-2), (NROW(intersect(generich[,16], generich[,16]))-1), (NROW(allprobes)-(NROW(intersect(generich[,16], generich[,16]))-1)), (NROW(intersect(allprobes, cluster3upHR))), lower.tail=FALSE)

phyper((NROW(intersect(generich[,16], cluster1downHR))-2), (NROW(intersect(generich[,16], generich[,16]))-1), (NROW(allprobes)-(NROW(intersect(generich[,16], generich[,16]))-1)), (NROW(intersect(allprobes, cluster1downHR))), lower.tail=FALSE)

phyper((NROW(intersect(generich[,16], cluster2downHR))-2), (NROW(intersect(generich[,16], generich[,16]))-1), (NROW(allprobes)-(NROW(intersect(generich[,16], generich[,16]))-1)), (NROW(intersect(allprobes, cluster2downHR))), lower.tail=FALSE)

phyper((NROW(intersect(generich[,16], cluster3downHR))-2), (NROW(intersect(generich[,16], generich[,16]))-1), (NROW(allprobes)-(NROW(intersect(generich[,16], generich[,16]))-1)), (NROW(intersect(allprobes, cluster3downHR))), lower.tail=FALSE)

##gene enrichment for AKT Pathway

phyper((NROW(intersect(generich[,17], cluster1upHR))-2), (NROW(intersect(generich[,17], generich[,17]))-1), (NROW(allprobes)-(NROW(intersect(generich[,17], generich[,17]))-1)), (NROW(intersect(allprobes, cluster1upHR))), lower.tail=FALSE)

phyper((NROW(intersect(generich[,17], cluster2upHR))-2), (NROW(intersect(generich[,17], generich[,17]))-1), (NROW(allprobes)-(NROW(intersect(generich[,17], generich[,17]))-1)), (NROW(intersect(allprobes, cluster2upHR))), lower.tail=FALSE)

phyper((NROW(intersect(generich[,17], cluster3upHR))-2), (NROW(intersect(generich[,17], generich[,17]))-1), (NROW(allprobes)-(NROW(intersect(generich[,17], generich[,17]))-1)), (NROW(intersect(allprobes, cluster3upHR))), lower.tail=FALSE)

phyper((NROW(intersect(generich[,17], cluster1downHR))-2), (NROW(intersect(generich[,17], generich[,17]))-1), (NROW(allprobes)-(NROW(intersect(generich[,17], generich[,17]))-1)), (NROW(intersect(allprobes, cluster1downHR))), lower.tail=FALSE)

phyper((NROW(intersect(generich[,17], cluster2downHR))-2), (NROW(intersect(generich[,17], generich[,17]))-1), (NROW(allprobes)-(NROW(intersect(generich[,17], generich[,17]))-1)), (NROW(intersect(allprobes, cluster2downHR))), lower.tail=FALSE)

phyper((NROW(intersect(generich[,17], cluster3downHR))-2), (NROW(intersect(generich[,17], generich[,17]))-1), (NROW(allprobes)-(NROW(intersect(generich[,17], generich[,17]))-1)), (NROW(intersect(allprobes, cluster3downHR))), lower.tail=FALSE)

##gene enrichment for IGF Pathway

phyper((NROW(intersect(generich[,18], cluster1upHR))-2), (NROW(intersect(generich[,18], generich[,18]))-1), (NROW(allprobes)-(NROW(intersect(generich[,18], generich[,18]))-1)), (NROW(intersect(allprobes, cluster1upHR))), lower.tail=FALSE)

phyper((NROW(intersect(generich[,18], cluster2upHR))-2), (NROW(intersect(generich[,18], generich[,18]))-1), (NROW(allprobes)-(NROW(intersect(generich[,18], generich[,18]))-1)), (NROW(intersect(allprobes, cluster2upHR))), lower.tail=FALSE)

phyper((NROW(intersect(generich[,18], cluster3upHR))-2), (NROW(intersect(generich[,18], generich[,18]))-1), (NROW(allprobes)-(NROW(intersect(generich[,18], generich[,18]))-1)), (NROW(intersect(allprobes, cluster3upHR))), lower.tail=FALSE)

phyper((NROW(intersect(generich[,18], cluster1downHR))-2), (NROW(intersect(generich[,18], generich[,18]))-1), (NROW(allprobes)-(NROW(intersect(generich[,18], generich[,18]))-1)), (NROW(intersect(allprobes, cluster1downHR))), lower.tail=FALSE)

phyper((NROW(intersect(generich[,18], cluster2downHR))-2), (NROW(intersect(generich[,18], generich[,18]))-1), (NROW(allprobes)-(NROW(intersect(generich[,18], generich[,18]))-1)), (NROW(intersect(allprobes, cluster2downHR))), lower.tail=FALSE)

phyper((NROW(intersect(generich[,18], cluster3downHR))-2), (NROW(intersect(generich[,18], generich[,18]))-1), (NROW(allprobes)-(NROW(intersect(generich[,18], generich[,18]))-1)), (NROW(intersect(allprobes, cluster3downHR))), lower.tail=FALSE)

##gene enrichment for PTEN Pathway

phyper((NROW(intersect(generich[,19], cluster1upHR))-2), (NROW(intersect(generich[,19], generich[,19]))-1), (NROW(allprobes)-(NROW(intersect(generich[,19], generich[,19]))-1)), (NROW(intersect(allprobes, cluster1upHR))), lower.tail=FALSE)

phyper((NROW(intersect(generich[,19], cluster2upHR))-2), (NROW(intersect(generich[,19], generich[,19]))-1), (NROW(allprobes)-(NROW(intersect(generich[,19], generich[,19]))-1)), (NROW(intersect(allprobes, cluster2upHR))), lower.tail=FALSE)

phyper((NROW(intersect(generich[,19], cluster3upHR))-2), (NROW(intersect(generich[,19], generich[,19]))-1), (NROW(allprobes)-(NROW(intersect(generich[,19], generich[,19]))-1)), (NROW(intersect(allprobes, cluster3upHR))), lower.tail=FALSE)

phyper((NROW(intersect(generich[,19], cluster1downHR))-2), (NROW(intersect(generich[,19], generich[,19]))-1), (NROW(allprobes)-(NROW(intersect(generich[,19], generich[,19]))-1)), (NROW(intersect(allprobes, cluster1downHR))), lower.tail=FALSE)

phyper((NROW(intersect(generich[,19], cluster2downHR))-2), (NROW(intersect(generich[,19], generich[,19]))-1), (NROW(allprobes)-(NROW(intersect(generich[,19], generich[,19]))-1)), (NROW(intersect(allprobes, cluster2downHR))), lower.tail=FALSE)

phyper((NROW(intersect(generich[,19], cluster3downHR))-2), (NROW(intersect(generich[,19], generich[,19]))-1), (NROW(allprobes)-(NROW(intersect(generich[,19], generich[,19]))-1)), (NROW(intersect(allprobes, cluster3downHR))), lower.tail=FALSE)

##gene enrichment for proapop Pathway

phyper((NROW(intersect(generich[,20], cluster1upHR))-2), (NROW(intersect(generich[,20], generich[,20]))-1), (NROW(allprobes)-(NROW(intersect(generich[,20], generich[,20]))-1)), (NROW(intersect(allprobes, cluster1upHR))), lower.tail=FALSE)

phyper((NROW(intersect(generich[,20], cluster2upHR))-2), (NROW(intersect(generich[,20], generich[,20]))-1), (NROW(allprobes)-(NROW(intersect(generich[,20], generich[,20]))-1)), (NROW(intersect(allprobes, cluster2upHR))), lower.tail=FALSE)

phyper((NROW(intersect(generich[,20], cluster3upHR))-2), (NROW(intersect(generich[,20], generich[,20]))-1), (NROW(allprobes)-(NROW(intersect(generich[,20], generich[,20]))-1)), (NROW(intersect(allprobes, cluster3upHR))), lower.tail=FALSE)

phyper((NROW(intersect(generich[,20], cluster1downHR))-2), (NROW(intersect(generich[,20], generich[,20]))-1), (NROW(allprobes)-(NROW(intersect(generich[,20], generich[,20]))-1)), (NROW(intersect(allprobes, cluster1downHR))), lower.tail=FALSE)

phyper((NROW(intersect(generich[,20], cluster2downHR))-2), (NROW(intersect(generich[,20], generich[,20]))-1), (NROW(allprobes)-(NROW(intersect(generich[,20], generich[,20]))-1)), (NROW(intersect(allprobes, cluster2downHR))), lower.tail=FALSE)

phyper((NROW(intersect(generich[,20], cluster3downHR))-2), (NROW(intersect(generich[,20], generich[,20]))-1), (NROW(allprobes)-(NROW(intersect(generich[,20], generich[,20]))-1)), (NROW(intersect(allprobes, cluster3downHR))), lower.tail=FALSE)

##gene enrichment for antiapop Pathway

phyper((NROW(intersect(generich[,21], cluster1upHR))-2), (NROW(intersect(generich[,21], generich[,21]))-1), (NROW(allprobes)-(NROW(intersect(generich[,21], generich[,21]))-1)), (NROW(intersect(allprobes, cluster1upHR))), lower.tail=FALSE)

phyper((NROW(intersect(generich[,21], cluster2upHR))-2), (NROW(intersect(generich[,21], generich[,21]))-1), (NROW(allprobes)-(NROW(intersect(generich[,21], generich[,21]))-1)), (NROW(intersect(allprobes, cluster2upHR))), lower.tail=FALSE)

phyper((NROW(intersect(generich[,21], cluster3upHR))-2), (NROW(intersect(generich[,21], generich[,21]))-1), (NROW(allprobes)-(NROW(intersect(generich[,21], generich[,21]))-1)), (NROW(intersect(allprobes, cluster3upHR))), lower.tail=FALSE)

phyper((NROW(intersect(generich[,21], cluster1downHR))-2), (NROW(intersect(generich[,21], generich[,21]))-1), (NROW(allprobes)-(NROW(intersect(generich[,21], generich[,21]))-1)), (NROW(intersect(allprobes, cluster1downHR))), lower.tail=FALSE)

phyper((NROW(intersect(generich[,21], cluster2downHR))-2), (NROW(intersect(generich[,21], generich[,21]))-1), (NROW(allprobes)-(NROW(intersect(generich[,21], generich[,21]))-1)), (NROW(intersect(allprobes, cluster2downHR))), lower.tail=FALSE)

phyper((NROW(intersect(generich[,21], cluster3downHR))-2), (NROW(intersect(generich[,21], generich[,21]))-1), (NROW(allprobes)-(NROW(intersect(generich[,21], generich[,21]))-1)), (NROW(intersect(allprobes, cluster3downHR))), lower.tail=FALSE)

##gene enrichment for chemokine Pathway

phyper((NROW(intersect(generich[,22], cluster1upHR))-2), (NROW(intersect(generich[,22], generich[,22]))-1), (NROW(allprobes)-(NROW(intersect(generich[,22], generich[,22]))-1)), (NROW(intersect(allprobes, cluster1upHR))), lower.tail=FALSE)

phyper((NROW(intersect(generich[,22], cluster2upHR))-2), (NROW(intersect(generich[,22], generich[,22]))-1), (NROW(allprobes)-(NROW(intersect(generich[,22], generich[,22]))-1)), (NROW(intersect(allprobes, cluster2upHR))), lower.tail=FALSE)

phyper((NROW(intersect(generich[,22], cluster3upHR))-2), (NROW(intersect(generich[,22], generich[,22]))-1), (NROW(allprobes)-(NROW(intersect(generich[,22], generich[,22]))-1)), (NROW(intersect(allprobes, cluster3upHR))), lower.tail=FALSE)

phyper((NROW(intersect(generich[,22], cluster1downHR))-2), (NROW(intersect(generich[,22], generich[,22]))-1), (NROW(allprobes)-(NROW(intersect(generich[,22], generich[,22]))-1)), (NROW(intersect(allprobes, cluster1downHR))), lower.tail=FALSE)

phyper((NROW(intersect(generich[,22], cluster2downHR))-2), (NROW(intersect(generich[,22], generich[,22]))-1), (NROW(allprobes)-(NROW(intersect(generich[,22], generich[,22]))-1)), (NROW(intersect(allprobes, cluster2downHR))), lower.tail=FALSE)

phyper((NROW(intersect(generich[,22], cluster3downHR))-2), (NROW(intersect(generich[,22], generich[,22]))-1), (NROW(allprobes)-(NROW(intersect(generich[,22], generich[,22]))-1)), (NROW(intersect(allprobes, cluster3downHR))), lower.tail=FALSE)

##gene enrichment for NFKB Pathway

phyper((NROW(intersect(generich[,24], cluster1upHR))-2), (NROW(intersect(generich[,24], generich[,24]))-1), (NROW(allprobes)-(NROW(intersect(generich[,24], generich[,24]))-1)), (NROW(intersect(allprobes, cluster1upHR))), lower.tail=FALSE)

phyper((NROW(intersect(generich[,24], cluster2upHR))-2), (NROW(intersect(generich[,24], generich[,24]))-1), (NROW(allprobes)-(NROW(intersect(generich[,24], generich[,24]))-1)), (NROW(intersect(allprobes, cluster2upHR))), lower.tail=FALSE)

phyper((NROW(intersect(generich[,24], cluster3upHR))-2), (NROW(intersect(generich[,24], generich[,24]))-1), (NROW(allprobes)-(NROW(intersect(generich[,24], generich[,24]))-1)), (NROW(intersect(allprobes, cluster3upHR))), lower.tail=FALSE)

phyper((NROW(intersect(generich[,24], cluster1downHR))-2), (NROW(intersect(generich[,24], generich[,24]))-1), (NROW(allprobes)-(NROW(intersect(generich[,24], generich[,24]))-1)), (NROW(intersect(allprobes, cluster1downHR))), lower.tail=FALSE)

phyper((NROW(intersect(generich[,24], cluster2downHR))-2), (NROW(intersect(generich[,24], generich[,24]))-1), (NROW(allprobes)-(NROW(intersect(generich[,24], generich[,24]))-1)), (NROW(intersect(allprobes, cluster2downHR))), lower.tail=FALSE)

phyper((NROW(intersect(generich[,24], cluster3downHR))-2), (NROW(intersect(generich[,24], generich[,24]))-1), (NROW(allprobes)-(NROW(intersect(generich[,24], generich[,24]))-1)), (NROW(intersect(allprobes, cluster3downHR))), lower.tail=FALSE)

##gene enrichment for notch Pathway

phyper((NROW(intersect(generich[,25], cluster1upHR))-2), (NROW(intersect(generich[,25], generich[,25]))-1), (NROW(allprobes)-(NROW(intersect(generich[,25], generich[,25]))-1)), (NROW(intersect(allprobes, cluster1upHR))), lower.tail=FALSE)

phyper((NROW(intersect(generich[,25], cluster2upHR))-2), (NROW(intersect(generich[,25], generich[,25]))-1), (NROW(allprobes)-(NROW(intersect(generich[,25], generich[,25]))-1)), (NROW(intersect(allprobes, cluster2upHR))), lower.tail=FALSE)

phyper((NROW(intersect(generich[,25], cluster3upHR))-2), (NROW(intersect(generich[,25], generich[,25]))-1), (NROW(allprobes)-(NROW(intersect(generich[,25], generich[,25]))-1)), (NROW(intersect(allprobes, cluster3upHR))), lower.tail=FALSE)

phyper((NROW(intersect(generich[,25], cluster1downHR))-2), (NROW(intersect(generich[,25], generich[,25]))-1), (NROW(allprobes)-(NROW(intersect(generich[,25], generich[,25]))-1)), (NROW(intersect(allprobes, cluster1downHR))), lower.tail=FALSE)

phyper((NROW(intersect(generich[,25], cluster2downHR))-2), (NROW(intersect(generich[,25], generich[,25]))-1), (NROW(allprobes)-(NROW(intersect(generich[,25], generich[,25]))-1)), (NROW(intersect(allprobes, cluster2downHR))), lower.tail=FALSE)

phyper((NROW(intersect(generich[,25], cluster3downHR))-2), (NROW(intersect(generich[,25], generich[,25]))-1), (NROW(allprobes)-(NROW(intersect(generich[,25], generich[,25]))-1)), (NROW(intersect(allprobes, cluster3downHR))), lower.tail=FALSE)

##gene enrichment for JAKSTAT Pathway

phyper((NROW(intersect(generich[,26], cluster1upHR))-2), (NROW(intersect(generich[,26], generich[,26]))-1), (NROW(allprobes)-(NROW(intersect(generich[,26], generich[,26]))-1)), (NROW(intersect(allprobes, cluster1upHR))), lower.tail=FALSE)

phyper((NROW(intersect(generich[,26], cluster2upHR))-2), (NROW(intersect(generich[,26], generich[,26]))-1), (NROW(allprobes)-(NROW(intersect(generich[,26], generich[,26]))-1)), (NROW(intersect(allprobes, cluster2upHR))), lower.tail=FALSE)

phyper((NROW(intersect(generich[,26], cluster3upHR))-2), (NROW(intersect(generich[,26], generich[,26]))-1), (NROW(allprobes)-(NROW(intersect(generich[,26], generich[,26]))-1)), (NROW(intersect(allprobes, cluster3upHR))), lower.tail=FALSE)

phyper((NROW(intersect(generich[,26], cluster1downHR))-2), (NROW(intersect(generich[,26], generich[,26]))-1), (NROW(allprobes)-(NROW(intersect(generich[,26], generich[,26]))-1)), (NROW(intersect(allprobes, cluster1downHR))), lower.tail=FALSE)

phyper((NROW(intersect(generich[,26], cluster2downHR))-2), (NROW(intersect(generich[,26], generich[,26]))-1), (NROW(allprobes)-(NROW(intersect(generich[,26], generich[,26]))-1)), (NROW(intersect(allprobes, cluster2downHR))), lower.tail=FALSE)

phyper((NROW(intersect(generich[,26], cluster3downHR))-2), (NROW(intersect(generich[,26], generich[,26]))-1), (NROW(allprobes)-(NROW(intersect(generich[,26], generich[,26]))-1)), (NROW(intersect(allprobes, cluster3downHR))), lower.tail=FALSE)

##gene enrichment for TGFB Pathway

phyper((NROW(intersect(generich[,27], cluster1upHR))-2), (NROW(intersect(generich[,27], generich[,27]))-1), (NROW(allprobes)-(NROW(intersect(generich[,27], generich[,27]))-1)), (NROW(intersect(allprobes, cluster1upHR))), lower.tail=FALSE)

phyper((NROW(intersect(generich[,27], cluster2upHR))-2), (NROW(intersect(generich[,27], generich[,27]))-1), (NROW(allprobes)-(NROW(intersect(generich[,27], generich[,27]))-1)), (NROW(intersect(allprobes, cluster2upHR))), lower.tail=FALSE)

phyper((NROW(intersect(generich[,27], cluster3upHR))-2), (NROW(intersect(generich[,27], generich[,27]))-1), (NROW(allprobes)-(NROW(intersect(generich[,27], generich[,27]))-1)), (NROW(intersect(allprobes, cluster3upHR))), lower.tail=FALSE)

phyper((NROW(intersect(generich[,27], cluster1downHR))-2), (NROW(intersect(generich[,27], generich[,27]))-1), (NROW(allprobes)-(NROW(intersect(generich[,27], generich[,27]))-1)), (NROW(intersect(allprobes, cluster1downHR))), lower.tail=FALSE)

phyper((NROW(intersect(generich[,27], cluster2downHR))-2), (NROW(intersect(generich[,27], generich[,27]))-1), (NROW(allprobes)-(NROW(intersect(generich[,27], generich[,27]))-1)), (NROW(intersect(allprobes, cluster2downHR))), lower.tail=FALSE)

phyper((NROW(intersect(generich[,27], cluster3downHR))-2), (NROW(intersect(generich[,27], generich[,27]))-1), (NROW(allprobes)-(NROW(intersect(generich[,27], generich[,27]))-1)), (NROW(intersect(allprobes, cluster3downHR))), lower.tail=FALSE)

##gene enrichment for Hedgehog Pathway

phyper((NROW(intersect(generich[,28], cluster1upHR))-2), (NROW(intersect(generich[,28], generich[,28]))-1), (NROW(allprobes)-(NROW(intersect(generich[,28], generich[,28]))-1)), (NROW(intersect(allprobes, cluster1upHR))), lower.tail=FALSE)

phyper((NROW(intersect(generich[,28], cluster2upHR))-2), (NROW(intersect(generich[,28], generich[,28]))-1), (NROW(allprobes)-(NROW(intersect(generich[,28], generich[,28]))-1)), (NROW(intersect(allprobes, cluster2upHR))), lower.tail=FALSE)

phyper((NROW(intersect(generich[,28], cluster3upHR))-2), (NROW(intersect(generich[,28], generich[,28]))-1), (NROW(allprobes)-(NROW(intersect(generich[,28], generich[,28]))-1)), (NROW(intersect(allprobes, cluster3upHR))), lower.tail=FALSE)

phyper((NROW(intersect(generich[,28], cluster1downHR))-2), (NROW(intersect(generich[,28], generich[,28]))-1), (NROW(allprobes)-(NROW(intersect(generich[,28], generich[,28]))-1)), (NROW(intersect(allprobes, cluster1downHR))), lower.tail=FALSE)

phyper((NROW(intersect(generich[,28], cluster2downHR))-2), (NROW(intersect(generich[,28], generich[,28]))-1), (NROW(allprobes)-(NROW(intersect(generich[,28], generich[,28]))-1)), (NROW(intersect(allprobes, cluster2downHR))), lower.tail=FALSE)

phyper((NROW(intersect(generich[,28], cluster3downHR))-2), (NROW(intersect(generich[,28], generich[,28]))-1), (NROW(allprobes)-(NROW(intersect(generich[,28], generich[,28]))-1)), (NROW(intersect(allprobes, cluster3downHR))), lower.tail=FALSE)

##gene enrichment for Wnt Pathway

phyper((NROW(intersect(generich[,29], cluster1upHR))-2), (NROW(intersect(generich[,29], generich[,29]))-1), (NROW(allprobes)-(NROW(intersect(generich[,29], generich[,29]))-1)), (NROW(intersect(allprobes, cluster1upHR))), lower.tail=FALSE)

phyper((NROW(intersect(generich[,29], cluster2upHR))-2), (NROW(intersect(generich[,29], generich[,29]))-1), (NROW(allprobes)-(NROW(intersect(generich[,29], generich[,29]))-1)), (NROW(intersect(allprobes, cluster2upHR))), lower.tail=FALSE)

phyper((NROW(intersect(generich[,29], cluster3upHR))-2), (NROW(intersect(generich[,29], generich[,29]))-1), (NROW(allprobes)-(NROW(intersect(generich[,29], generich[,29]))-1)), (NROW(intersect(allprobes, cluster3upHR))), lower.tail=FALSE)

phyper((NROW(intersect(generich[,29], cluster1downHR))-2), (NROW(intersect(generich[,29], generich[,29]))-1), (NROW(allprobes)-(NROW(intersect(generich[,29], generich[,29]))-1)), (NROW(intersect(allprobes, cluster1downHR))), lower.tail=FALSE)

phyper((NROW(intersect(generich[,29], cluster2downHR))-2), (NROW(intersect(generich[,29], generich[,29]))-1), (NROW(allprobes)-(NROW(intersect(generich[,29], generich[,29]))-1)), (NROW(intersect(allprobes, cluster2downHR))), lower.tail=FALSE)

phyper((NROW(intersect(generich[,29], cluster3downHR))-2), (NROW(intersect(generich[,29], generich[,29]))-1), (NROW(allprobes)-(NROW(intersect(generich[,29], generich[,29]))-1)), (NROW(intersect(allprobes, cluster3downHR))), lower.tail=FALSE)

##gene enrichment for ESC Pathway

phyper((NROW(intersect(generich[,30], cluster1upHR))-2), (NROW(intersect(generich[,30], generich[,30]))-1), (NROW(allprobes)-(NROW(intersect(generich[,30], generich[,30]))-1)), (NROW(intersect(allprobes, cluster1upHR))), lower.tail=FALSE)

phyper((NROW(intersect(generich[,30], cluster2upHR))-2), (NROW(intersect(generich[,30], generich[,30]))-1), (NROW(allprobes)-(NROW(intersect(generich[,30], generich[,30]))-1)), (NROW(intersect(allprobes, cluster2upHR))), lower.tail=FALSE)

phyper((NROW(intersect(generich[,30], cluster3upHR))-2), (NROW(intersect(generich[,30], generich[,30]))-1), (NROW(allprobes)-(NROW(intersect(generich[,30], generich[,30]))-1)), (NROW(intersect(allprobes, cluster3upHR))), lower.tail=FALSE)

phyper((NROW(intersect(generich[,30], cluster1downHR))-2), (NROW(intersect(generich[,30], generich[,30]))-1), (NROW(allprobes)-(NROW(intersect(generich[,30], generich[,30]))-1)), (NROW(intersect(allprobes, cluster1downHR))), lower.tail=FALSE)

phyper((NROW(intersect(generich[,30], cluster2downHR))-2), (NROW(intersect(generich[,30], generich[,30]))-1), (NROW(allprobes)-(NROW(intersect(generich[,30], generich[,30]))-1)), (NROW(intersect(allprobes, cluster2downHR))), lower.tail=FALSE)

phyper((NROW(intersect(generich[,30], cluster3downHR))-2), (NROW(intersect(generich[,30], generich[,30]))-1), (NROW(allprobes)-(NROW(intersect(generich[,30], generich[,30]))-1)), (NROW(intersect(allprobes, cluster3downHR))), lower.tail=FALSE)

##to test for duplicate probe sets in Pathways

duplicated(generich[1:75,8]

##to test for difference in cluster descriptives

data <- read.csv(file="C:/All Pathways and clinical.csv", header=TRUE, row.names=1)

stg1 <- (data$stage==1)

stg2 <- (data$stage==2)

stg3 <- (data$stage==3)

lig <- (data$grade==0)

hg <- (data$grade==1)

male <- (data$male==1)

female <- (data$male==0)

chisq.test(stg1, data$cluster)

chisq.test(stg2, data$cluster)

chisq.test(stg3, data$cluster)

chisq.test(data$age, data$cluster)

##Pathology Survival

##+/-

summary(coxph(Surv(months5,dead5)~(acinarness>0)+(solidness>0)+(papness>0)+(bacness>0), data=data))

##%

summary(coxph(Surv(months5,dead5)~(acinarness)+(solidness)+(papness)+(bacness), data=data))
