# Supplementary material for: Clinically Relevant Characterization of Lung Adenocarcinoma Subtypes Based on Cellular Pathways: An International Validation Study
Source: PLoS One. 2010 Jul 22;5(7):e11712. doi: 10.1371/journal.pone.0011712 (PMC2908611; doi:10.1371/journal.pone.0011712)
Supplement: References S1 — References for gene lists (0.05 MB DOC) [file pone.0011712.s007.doc]

**References for Gene Lists**

**mTOR**

1. Wouters BG, Koritzinsky M. Hypoxia signaling through mTOR and the unfolded protein response in cancer. Nat Rev Cancer. 2008; 8:851-864.
2. Gridelli C, Maione P, Rossi A. The potential role of mTOR inhibitors in non-small cell lung cancer. Oncologist. 2008; 13:139-147.
3. Chiang GG, Abraham RT. Targeting the mTOR signaling network in cancer. Trends Mol Med. 2007; 13:433-442.
4. Wullschleger S, Loewith R, Hall MN. TOR signaling in growth and metabolism. Cell. 2006; 124:471-484.

**Cell Cycle (stimulating and inhibitory)**

1. Coqueret O. Linking cyclins to transcriptional control. Gene. 2002; 299:35-55.
2. Neganova I, Lako M. G1 to S phase cell cycle transition in somatic and embryonic stem cells. J Anat. 2008; 213:30-44.
3. Dash BC, El-Deiry WS. Cell cycle checkpoint control mechanisms that can be disrupted in cancer. Methods Mol Biol. 2004; 280:99-161.
4. Allshire RC, Karpen GH. Epigenetic regulation of centromeric chromatin: old dogs, new tricks? Nat Rev Genet. 2008; 9:923-937.
5. Eastman A. Cell cycle checkpoints and their impact on anticancer therapeutic strategies. J Cell Biochem. 2004; 91:223-231.
6. Taylor WR, Stark GR. Regulation of the G2/M transition by p53. Oncogene. 2001; 20:1803-1815.
7. Molinari M. Cell cycle checkpoints and their inactivation in human cancer. Cell Prolif. 2000; 33: 261-274.

**Interferons**

1. Pestka S, Krause CD, Walter MR. Interferons, interferon-like cytokines, and their receptors. Immunol Rev. 2004; 202:8-32.
2. Vilcek J. Novel interferons. Nat Immunol. 2003; 4:8-9.
3. David M. Signal transduction by type I interferons. Biotechniques. 2002;Suppl:58-65.
4. Randall RE, Goodbourn S. Interferons and viruses: an interplay between induction, signalling, antiviral responses and virus countermeasures. J Gen Virol. 2008; 89:1-47.
5. Borden EC, Sen GC, Uze G, Silverman RH, Ransohoff RM, Foster GR, Stark GR. Interferons at age 50: past, current and future impact on biomedicine. Nat Rev Drug Discov. 2007; 6:975-990.

**Interleukins (stimulating and inhibitory)**

1. Apte RN, Voronov E. Is interleukin-1 a good or bad 'guy' in tumor immunobiology and immunotherapy? Immunol Rev. 2008; 222:222-241.
2. Naugler WE, Karin M. The wolf in sheep's clothing: the role of interleukin-6 in immunity, inflammation and cancer. Trends Mol Med. 2008; 14:109-19.
3. Mosser DM, Zhang X. Interleukin-10: new perspectives on an old cytokine. Immunol Rev. 2008; 226:205-218.
4. Lyakh L, Trinchieri G, Provezza L, Carra G, Gerosa F. Regulation of interleukin-12/interleukin-23 production and the T-helper 17 response in humans. Immunol Rev. 2008; 226:112-131.
5. Waugh DJ, Wilson C. The interleukin-8 pathway in cancer. Clin Cancer Res. 2008; 14:6735-6741.
6. Olejniczak K, Kasprzak A. Biological properties of interleukin 2 and its role in pathogenesis of selected diseases--a review. Med Sci Monit. 2008; 14:179-189.
7. Andorsky DJ, Timmerman JM. Interleukin-21: biology and application to cancer therapy. Expert Opin Biol Ther. 2008; 8:1295-1307.
8. Francipane MG, Alea MP, Lombardo Y, Todaro M, Medema JP, Stassi G. Crucial role of interleukin-4 in the survival of colon cancer stem cells. Cancer Res. 2008; 68:4022-4025.
9. Park S, Cheon S, Cho D. The dual effects of interleukin-18 in tumor progression. Cell Mol Immunol. 2007; 4:329-335.

**EGFR**

1. Zips D, Krause M, Yaromina A, Dörfler A, Eicheler W, Schütze C, Gurtner K, Baumann M. Epidermal growth factor receptor inhibitors for radiotherapy: biological rationale and preclinical results. J Pharm Pharmacol. 2008; 60:1019-1028.
2. Ciardiello F, Tortora G. EGFR antagonists in cancer treatment. N Engl J Med. 2008; 358:1160-1174.
3. Uberall I, Kolár Z, Trojanec R, Berkovcová J, Hajdúch M. The status and role of ErbB receptors in human cancer. Exp Mol Pathol. 2008; 84:79-89.

**PDGF**

1. Wang Z, Kong D, Li Y, Sarkar FH. PDGF-D signaling: a novel target in cancer therapy. Curr Drug Targets. 2009; 10:38-41.
2. Alvarez RH, Kantarjian HM, Cortes JE. Biology of platelet-derived growth factor and its involvement in disease. Mayo Clin Proc. 2006; 81:1241-1257.

**Angiogenesis and Hypoxia**

1. Goldfarb M. Signaling by fibroblast growth factors: the inside story. Sci STKE. 2001; 2001:PE37.
2. Augustin HG, Koh GY, Thurston G, Alitalo K. Control of vascular morphogenesis and homeostasis through the angiopoietin-Tie system. Nat Rev Mol Cell Biol. 2009 Mar;10(3):165-177.
3. Bertout JA, Patel SA, Simon MC. The impact of O2 availability on human cancer. Nat Rev Cancer. 2008; 8:967-975.
4. Murdoch C, Muthana M, Coffelt SB, Lewis CE. The role of myeloid cells in the promotion of tumour angiogenesis. Nat Rev Cancer. 2008; 8:618-631.
5. Ellis LM, Hicklin DJ. VEGF-targeted therapy: mechanisms of anti-tumour activity. Nat Rev Cancer. 2008; 8:579-591.
6. Dewhirst MW, Cao Y, Moeller B. Cycling hypoxia and free radicals regulate angiogenesis and radiotherapy response. Nat Rev Cancer. 2008 Jun;8(6):425-437.

**AKT/PI3K and PTEN**

1. Ihle NT, Powis G. Take your PIK: phosphatidylinositol 3-kinase inhibitors race through the clinic and toward cancer therapy. Mol Cancer Ther. 2009; 8:1-9.
2. Franke TF. PI3K/Akt: getting it right matters. Oncogene. 2008; 27:6473-6488.
3. Yin Y, Shen WH. PTEN: a new guardian of the genome. Oncogene. 2008; 27:5443-5453.
4. Hennessy BT, Smith DL, Ram PT, Lu Y, Mills GB. Exploiting the PI3K/AKT pathway for cancer drug discovery. Nat Rev Drug Discov. 2005; 4:988-1004.
5. Vivanco I, Sawyers CL. The phosphatidylinositol 3-Kinase AKT pathway in human cancer. Nat Rev Cancer. 2002; 2:489-501.
6. Testa JR, Bellacosa A. AKT plays a central role in tumorigenesis. Proc Natl Acad Sci U S A. 2001; 98:10983-10985.

**IGF-1**

1. Vincent AM, Feldman EL. Control of cell survival by IGF signaling pathways. Growth Horm IGF Res. 2002;12: 193-197.
2. Pollak M. Insulin and insulin-like growth factor signalling in neoplasia. Nat Rev Cancer. 2008; 8:915-928.
3. Miller BS, Yee D. Type I insulin-like growth factor receptor as a therapeutic target in cancer. Cancer Res. 2005; 65:10123-10127.

**Pro- and Anti-Apoptosis**

1. Haupt S, Berger M, Goldberg Z, Haupt Y. Apoptosis - the p53 network. J Cell Sci. 2003; 116:4077-4085.
2. Kang MH, Reynolds CP. Bcl-2 inhibitors: targeting mitochondrial apoptotic pathways in cancer therapy. Clin Cancer Res. 2009; 15:1126-1132.
3. Vazquez A, Bond EE, Levine AJ, Bond GL. The genetics of the p53 pathway, apoptosis and cancer therapy. Nat Rev Drug Discov. 2008; 7:979-987.
4. Melet A, Song K, Bucur O, Jagani Z, Grassian AR, Khosravi-Far R. Apoptotic pathways in tumor progression and therapy. Adv Exp Med Biol. 2008; 615:47-79.
5. Ashkenazi A. Directing cancer cells to self-destruct with pro-apoptotic receptor agonists. Nat Rev Drug Discov. 2008; 7:1001-1012.
6. Reimers K, Choi CY, Bucan V, Vogt PM. The Bax Inhibitor-1 (BI-1) family in apoptosis and tumorigenesis. Curr Mol Med. 2008; 8:148-156.
7. Danial NN. BCL-2 family proteins: critical checkpoints of apoptotic cell death. Clin Cancer Res. 2007; 13:7254-7263.

**Chemokine**

1. Jin T, Xu X, Hereld D. Chemotaxis, chemokine receptors and human disease. Cytokine. 2008; 44:1-8.
2. Vandercappellen J, Van Damme J, Struyf S. The role of CXC chemokines and their receptors in cancer. Cancer Lett. 2008; 267:226-244.
3. Zlotnik A. New insights on the role of CXCR4 in cancer metastasis. J Pathol. 2008; 215:211-213.
4. O'Hayre M, Salanga CL, Handel TM, Allen SJ. Chemokines and cancer: migration, intracellular signalling and intercellular communication in the microenvironment. Biochem J. 2008; 409:635-649.

**NFKB**

1. Pomerantz JL, Baltimore D. Two pathways to NF-kappaB. Mol Cell. 2002; 10:693-695.
2. Cortés Sempere M, Rodríguez Fanjul V, Sánchez Pérez I, Perona R. The role of the NFkappaB signalling pathway in cancer. lin Transl Oncol. 2008; 10:143-147.
3. Baud V, Karin M. Is NF-kappaB a good target for cancer therapy? Hopes and pitfalls. Nat Rev Drug Discov. 2009; 8:33-40.
4. Maeda S, Omata M. Inflammation and cancer: role of nuclear factor-kappaB activation. Cancer Sci. 2008; 99:836-842.
5. Van Waes C. Nuclear factor-kappaB in development, prevention, and therapy of cancer. Clin Cancer Res. 2007; 13:1076-1082.

**Notch**

1. Rizzo P, Osipo C, Foreman K, Golde T, Osborne B, Miele L. Rational targeting of Notch signaling in cancer. Oncogene. 2008; 27:5124-5131.
2. Dufraine J, Funahashi Y, Kitajewski J. Notch signaling regulates tumor angiogenesis by diverse mechanisms. Oncogene. 2008; 27:5132-5127.
3. Yan M, Plowman GD. Delta-like 4/Notch signaling and its therapeutic implications. Clin Cancer Res. 2007; 13:7243-7246.
4. Shih IeM, Wang TL. Notch signaling, gamma-secretase inhibitors, and cancer therapy. Cancer Res. 2007; 67:1879-1882.

**TGFB**

1. Li MO, Flavell RA. TGF-beta: a master of all T cell trades. Cell. 2008; 134:392-404.
2. Massagué J. TGFbeta in Cancer. Cell. 2008; 134:215-230.
3. Schmierer B, Hill CS. TGFbeta-SMAD signal transduction: molecular specificity and functional flexibility. Nat Rev Mol Cell Biol. 2007; 8:970-982.
4. Yingling JM, Blanchard KL, Sawyer JS. Development of TGF-beta signalling inhibitors for cancer therapy. Nat Rev Drug Discov. 2004; 3:1011-1022.
5. Bierie B, Moses HL. Tumour microenvironment: TGFbeta: the molecular Jekyll and Hyde of cancer. Nat Rev Cancer. 2006 Jul;6(7):506-520.

**Wnt**

1. Katoh M, Katoh M. WNT signaling pathway and stem cell signaling network. Clin Cancer Res. 2007; 13:4042-4045.
2. Reya T, Clevers H. Wnt signalling in stem cells and cancer. Nature. 2005; 434:843-850.
3. Malhotra S, Kincade PW. Wnt-related molecules and signaling pathway equilibrium in hematopoiesis. Cell Stem Cell. 2009; 4:27-36.
4. Clevers H. Wnt/beta-catenin signaling in development and disease. Cell. 2006; 127:469-580.
5. Moon RT, Kohn AD, De Ferrari GV, Kaykas A. WNT and beta-catenin signalling: diseases and therapies. Nat Rev Genet. 2004; 5:691-701.

**B-cell**

1. Tolar P, Sohn HW, Pierce SK. Viewing the antigen-induced initiation of B-cell activation in living cells. Immunol Rev. 2008; 221:64-76.
2. Lanzavecchia A, Bernasconi N, Traggiai E, Ruprecht CR, Corti D, Sallusto F. Understanding and making use of human memory B cells. Immunol Rev. 2006; 211:303-309.
3. Shlomchik MJ. Sites and stages of autoreactive B cell activation and regulation. Immunity. 2008; 28:18-28.
4. Harwood NE, Batista FD. New insights into the early molecular events underlying B cell activation. Immunity. 2008; 28:609-619.

**T-cell**

1. Cronin SJ, Penninger JM. From T-cell activation signals to signaling control of anti-cancer immunity. Immunol Rev. 2007; 220:151-168.
2. Burroughs NJ, van der Merwe PA. Stochasticity and spatial heterogeneity in T-cell activation. Immunol Rev. 2007; 216:69-80.

**Antigen**

1. Batista FD, Harwood NE. The who, how and where of antigen presentation to B cells. Nat Rev Immunol. 2009; 9:15-27.

**Complement**

1. Kirschfink M. Targeting complement in therapy. Immunol Rev. 2001; 180:177-189.
2. Marsh JE, Pratt JR, Sacks SH. Targeting the complement system. Curr Opin Nephrol Hypertens. 1999; 8:557-562.
3. Toapanta FR, Ross TM. Complement-mediated activation of the adaptive immune responses: role of C3d in linking the innate and adaptive immunity. Immunol Res. 2006; 36:197-210.

**Hedgehog**

1. Katoh Y, Katoh M. Hedgehog signaling, epithelial-to-mesenchymal transition and miRNA. Int J Mol Med. 2008; 22:271-275.
2. Jacob L, Lum L. Hedgehog signaling pathway. Sci STKE. 2007; 2007:cm6.
3. Katoh M. Networking of WNT, FGF, Notch, BMP, and Hedgehog signaling pathways during carcinogenesis. Stem Cell Rev. 2007; 3:30-38.
4. Chari NS, McDonnell TJ. The sonic hedgehog signaling network in development and neoplasia. Adv Anat Pathol. 2007; 14:344-352.

**JAK/STAT**

1. Aaronson DS, Horvath CM. A road map for those who don't know JAK-STAT. Science. 2002 May; 296:1653-1655.
2. Nefedova Y, Gabrilovich DI. Targeting of Jak/STAT pathway in antigen presenting cells in cancer. Curr Cancer Drug Targets. 2007; 7:71-77.
3. Boudny V, Kovarik J. JAK/STAT signaling pathways and cancer. Janus kinases/signal transducers and activators of transcription. Neoplasma. 2002; 49:349-355.
