# Supplementary material for: Clinically Relevant Characterization of Lung Adenocarcinoma Subtypes Based on Cellular Pathways: An International Validation Study
Source: PLoS One. 2010 Jul 22;5(7):e11712. doi: 10.1371/journal.pone.0011712 (PMC2908611; doi:10.1371/journal.pone.0011712)
Supplement: Table S1 — French validation cluster descriptives. (0.04 MB DOC) [file pone.0011712.s009.doc]

| **French Cluster Descriptives** |  |  |  |  |  |
| --- | --- | --- | --- | --- | --- |
| **Variable** | **Overall (%)** | **Cluster 1 (%)** | **Cluster 2 (%)** | **Cluster 3 (%)** | **p-value** |
| **Number of Tumors** | 89 (100) | 23 (26) | 34 (38) | 32 (36) | N/A |
| **Stage 1** | 84 (94) | 23 (27) | 34 (41) | 27 (32) | 0.33 |
| **Stage 2** | 4 (4) | 0 | 0 | 4 (100) | 0.018 |
| **Stage 3** | 1 (1) | 0 | 0 | 1 (100) | 0.37 |
| **Unknown Stage** | 0 | 0 | 0 | 0 | N/A |
| **Male** | 18 (20) | 9 (50) | 4 (22) | 5 (38) | 0.31 |
| **Female** | 71 (80) | 14 (20) | 30 (42) | 27 (38) | 0.047 |
| **Age at Diagnosis** | 61.8 | 64.3 | 59.6 | 61 | 0.38 |
| **Percent BAC (Mean)** | 16.9 | 31.1 | 15.6 | 4.1 | <0.0001 |
| **Percent Papillary (Mean)** | 20.8 | 18.3 | 30.4 | 13.8 | 0.35 |
| **Percent Acinar (Mean)** | 18.0 | 13.7 | 22.5 | 17.7 | 0.59 |
| **Percent Solid (Mean)** | 26.8 | 10.7 | 18.4 | 51.4 | <0.0001 |
