# Supplementary material for: Clinically Relevant Characterization of Lung Adenocarcinoma Subtypes Based on Cellular Pathways: An International Validation Study
Source: PLoS One. 2010 Jul 22;5(7):e11712. doi: 10.1371/journal.pone.0011712 (PMC2908611; doi:10.1371/journal.pone.0011712)
Supplement: Table S2 — French gene enrichment p-values. (0.05 MB DOC) [file pone.0011712.s010.doc]

| **Pathway Name** | **Cluster 1 (+)** | **Cluster 2 (+)** | **Cluster 3 (+)** | **Cluster 1 (-)** | **Cluster 2 (-)** | **Cluster 3 (-)** |
| --- | --- | --- | --- | --- | --- | --- |
| **Cell cycle down** | 0.27 | NS | NS | 0.18 | NS | NS |
| **Il pos** | NS | NS | NS | 0.19 | 0.31 | NS |
| **EGFR** | 0.29 | NS | NS | NS | 0.052 | NS |
| **Angiogenesis** | 0.33 | NS | NS | 0.024 | 0.0084 | NS |
| **AKT** | 0.091 | NS | NS | NS | 0.43 | NS |
| **IGF** | NS | 0.14 | NS | NS | NS | NS |
| **PTEN** | 0.27 | NS | NS | NS | NS | NS |
| **Proapop** | NS | NS | NS | <0.0001 | 0.27 | NS |
| **Antiapop** | NS | NS | NS | 0.00087 | 0.0069 | NS |
| **Chemokine** | NS | NS | NS | 0.15 | <0.0001 | NS |
| **Cell cycle pos** | 0.78 | <0.0001 | NS | NS | 0.0052 | NS |
| **NFKB** | 0.42 | NS | NS | 0.28 | 0.12 | NS |
| **Notch** | NS | NS | 0.017 | 0.20 | NS | NS |
| **JAKSTAT** | NS | NS | NS | 0.19 | 0.00037 | NS |
| **TGFB** | NS | NS | NS | 0.20 | NS | NS |
| **Hedgehog** | 0.16 | NS | NS | NS | NS | 0.081 |
| **Wnt** | NS | 0.57 | NS | NS | NS | 0.062 |
| **ESC** | 0.31 | <0.0001 | 0.32 | 0.96 | <0.0001 | 0.12 |
| **Tcell** | NS | NS | NS | 0.035 | <0.0001 | NS |
| **Antigen** | NS | NS | NS | NS | 0.0090 | NS |
