# Supplementary material for: Clinically Relevant Characterization of Lung Adenocarcinoma Subtypes Based on Cellular Pathways: An International Validation Study
Source: PLoS One. 2010 Jul 22;5(7):e11712. doi: 10.1371/journal.pone.0011712 (PMC2908611; doi:10.1371/journal.pone.0011712)
Supplement: Table S3 — French validation of pathway survival. (0.03 MB DOC) [file pone.0011712.s011.doc]

| **Pathway Name or Other Variable** | **Coefficient** | **P-value** |
| --- | --- | --- |
| **Proapop** | -0.84 | 0.036 |
| **JAK/STAT** | 0.97 | 0.022 |
| **Complement** | -0.47 | 0.080 |
| **Antigen** | -1.65 | 0.0023 |
| **IGF-1** | -0.83 | 0.022 |
| **AKT/PI3K** | 0.62 | 0.043 |
| **Angiogenesis** | 0.52 | 0.12 |
| **IL-suppressive** | 1.33 | 0.00021 |
| **IL-stimulatory** | 0.94 | 0.018 |
| **Interferon** | -0.75 | 0.013 |
| **Cell cycle (-)** | 0.44 | 0.16 |
| **mTOR** | 0.88 | 0.0035 |
