# Supplementary material for: Clinically Relevant Characterization of Lung Adenocarcinoma Subtypes Based on Cellular Pathways: An International Validation Study
Source: PLoS One. 2010 Jul 22;5(7):e11712. doi: 10.1371/journal.pone.0011712 (PMC2908611; doi:10.1371/journal.pone.0011712)
Supplement: Table S4 — French validation of pathway-pathology interactions. (0.06 MB DOC) [file pone.0011712.s012.doc]

|  | **BAC-ness (%)** | | **Acinar- ness (%)** | | **Papillary-ness (%)** | | **Solid-ness (%)** | |
| --- | --- | --- | --- | --- | --- | --- | --- | --- |
| **Pathway Name** | **Coefficient** | **P-value** | **Coefficient** | **P-value** | **Coefficient** | **P-value** | **Coefficient** | **P-value** |
| **Intercept** | 15.45 | <0.0001 | 18.48 | <0.0001 | 1.29 | <0.0001 | 28.26 | <0.0001 |
| **Cell Cycle (+)** | NA | NA | 5.44 | 0.080 | NA | NA | NA | NA |
| **ESC** | NA | NA | NA | NA | NA | NA | NA | NA |
| **B-cell** | NA | NA | NA | NA | NA | NA | NA | NA |
| **T-cell** | NA | NA | NA | NA | NA | NA | NA | NA |
| **Antigen** | NA | NA | NA | NA | NA | NA | NA | NA |
| **AKT/PI3K** | 4.83 | 0.052 | NA | NA | -9.07 | 0.00034 | 4.39 | 0.17 |
| **IGF-1** | NA | NA | NA | NA | -5.26 | 0.057 | NA | NA |
| **Chemokine** | NA | NA | NA | NA | NA | NA | NA | NA |
| **NF-κB** | NA | NA | NA | NA | NA | NA | NA | NA |
| **Notch** | -4.86 | 0.060 | NA | NA | NA | NA | NA | NA |
| **JAK/STAT** | -3.84 | 0.15 | NA | NA | NA | NA | 5.42 | 0.077 |
| **Complement** | 4.61 | 0.067 | NA | NA | 6.91 | 0.0033 | -9.39 | 0.00093 |
| **mTOR** | NA | NA | -4.09 | 0.16 | 4.19 | 0.13 | 8.00 | 0.0089 |
| **Cell Cycle (-)** | NA | NA | -4.70 | 0.0436 | NA | NA | NA | NA |
| **Angiogenesis** | 7.44 | 0.021 | -7.12 | 0.012 | NA | NA | NA | NA |
| **IL-stimulatory** | -8.98 | 0.0024 | 7.50 | 0.012 | -12.85 | <0.0001 | 13.23 | <0.0001 |
| **IL-suppressive** | NA | NA | NA | NA | NA | NA | NA | NA |
| **Interferon** | NA | NA | -4.15 | 0.063 | 6.04 | 0.0091 | NA | NA |
| **EGFR** | NA | NA | NA | NA | 5.19 | 0.025 | NA | NA |
| **PDGF** | NA | NA | 5.12 | 0.030 | NA | NA | -4.68 | 0.15 |
| **Hypoxia** | 5.20 | 0.064 | NA | NA | -6.14 | 0.012 | NA | NA |
| **PTEN** | NA | NA | 4.22 | 0.084 | NA | NA | NA | NA |
| **Pro-apoptosis** | NA | NA | NA | NA | NA | NA | NA | NA |
| **Anti-apoptosis** | NA | NA | NA | NA | 4.41 | 0.079 | NA | NA |
| **TGF-β** | NA | NA | NA | NA | NA | NA | -6.28 | 0.053 |
| **Hedgehog** | NA | NA | NA | NA | NA | NA | NA | NA |
| **Wnt** | NA | NA | NA | NA | 10.53 | <0.0001 | -7.65 | 0.011 |
