# Supplementary material for: Clinically Relevant Characterization of Lung Adenocarcinoma Subtypes Based on Cellular Pathways: An International Validation Study
Source: PLoS One. 2010 Jul 22;5(7):e11712. doi: 10.1371/journal.pone.0011712 (PMC2908611; doi:10.1371/journal.pone.0011712)
Supplement: Table S5 — Japanese cluster descriptives. (0.04 MB DOC) [file pone.0011712.s013.doc]

| **Variable** | **Overall (%)** | **Cluster 1 (%)** | **Cluster 2 (%)** | **Cluster 3 (%)** | **p-value** |
| --- | --- | --- | --- | --- | --- |
| **Number of Tumors** | 87 (100) | 30 (34) | 28 (32) | 29 (33) | N/A |
| **Stage 1** | 49 (56) | 21 (43) | 12 (24) | 16 (33) | 0.11 |
| **Stage 2** | 13 (15) | 2 (15) | 5 (38) | 6 (46) | 0.28 |
| **Stage 3** | 25 (29) | 7 (28) | 11 (44) | 7 (28) | 0.32 |
| **Unknown Stage** | 0 | 0 | 0 | 0 | N/A |
| **High Grade** | 33 (38) | 2 (6) | 14 (42) | 17 (52) | <0.0001 |
| **Low/ Intermediate Grade** | 54 (62) | 28 (52) | 14 (26) | 12 (22) | <0.0001 |
| **Unknown Grade** | 0 | 0 | 0 | 0 | N/A |
| **Male** | 46 (53) | 13 (28) | 15 (33) | 18 (39) | 0.35 |
| **Female** | 41 (47) | 17 (41) | 13 (32) | 11 (27) | 0.35 |
| **Age at Diagnosis** | 60.9 | 60.4 | 60.8 | 61.4 | 0.30 |
| **Percent BAC (Mean)** | 17.7 | 35.2 | 7.9 | 9.1 | <0.0001 |
| **Percent Papillary (Mean)** | 30.7 | 33.8 | 26.6 | 31.4 | 0.62 |
| **Percent Acinar (Mean)** | 27.9 | 22.7 | 39.1 | 22.4 | 0.05 |
| **Percent Solid (Mean)** | 15.8 | 1.7 | 21.3 | 25.2 | 0.00 |
