# Supplementary material for: Clinically Relevant Characterization of Lung Adenocarcinoma Subtypes Based on Cellular Pathways: An International Validation Study
Source: PLoS One. 2010 Jul 22;5(7):e11712. doi: 10.1371/journal.pone.0011712 (PMC2908611; doi:10.1371/journal.pone.0011712)
Supplement: Table S6 — Japanese gene enrichment p-values. (0.04 MB DOC) [file pone.0011712.s014.doc]

| **Pathway Name** | **Cluster 1 (+)** | **Cluster 2 (+)** | **Cluster 3 (+)** | **Cluster 1 (-)** | **Cluster 2 (-)** | **Cluster 3 (-)** |
| --- | --- | --- | --- | --- | --- | --- |
| **anti-apoptosis** | 0.019 | NS | 0.033 | NS | NS | NS |
| **PDGF** | 0.044 | NS | NS | NS | NS | NS |
| **Cell Cycle (-)** | NS | 0.0047 | NS | 0.015 | NS | NS |
| **mTOR** | NS | NS | <0.0001 | 0.025 | 0.031 | 0.029 |
| **Cell Cycle (+)** | NS | NS | 0.0035 | <0.0001 | 0.021 | 0.0038 |
| **notch** | NS | NS | 0.022 | 0.020 | NS | 0.023 |
| **ESC** | NS | NS | <0.0001 | <0.0001 | NS | NS |
| **JAK** | NS | NS | NS | 0.037 | 0.046 | 0.0038 |
| **Antigen** | NS | NS | NS | NS | 0.014 | 0.013 |
| **NFKB** | NS | NS | NS | NS | 0.032 | 0.028 |
| **PTEN** | NS | NS | NS | NS | 0.039 | 0.035 |
| **EGFR** | NS | NS | NS | NS | NS | 0.049 |
| **IGF-1** | NS | NS | NS | NS | NS | 0.00030 |
