# Supplementary material for: Clinically Relevant Characterization of Lung Adenocarcinoma Subtypes Based on Cellular Pathways: An International Validation Study
Source: PLoS One. 2010 Jul 22;5(7):e11712. doi: 10.1371/journal.pone.0011712 (PMC2908611; doi:10.1371/journal.pone.0011712)
Supplement: Table S7 — Japanese pathway survival. (0.03 MB DOC) [file pone.0011712.s015.doc]

| **Pathway Name or Other Variable** | **Coefficient** | **P-value** |
| --- | --- | --- |
| **Stage 2** | 0.39 | 0.43 |
| **Stage 3** | 2.14 | <0.0001 |
| **Cell Cycle (+)** | 0.48 | 0.020 |
| **PDGF** | 0.63 | 0.0040 |
| **Angiogenesis** | -0.33 | 0.16 |
| **AKT/PI3K** | 0.67 | 0.0020 |
| **B-cell** | -0.38 | 0.14 |
| **Complement** | -0.85 | 0.00022 |
| **mTOR** | 0.62 | 0.0019 |
