# Supplementary material for: Clinically Relevant Characterization of Lung Adenocarcinoma Subtypes Based on Cellular Pathways: An International Validation Study
Source: PLoS One. 2010 Jul 22;5(7):e11712. doi: 10.1371/journal.pone.0011712 (PMC2908611; doi:10.1371/journal.pone.0011712)
Supplement: Table S9 — Validation overall statistics (0.03 MB DOC) [file pone.0011712.s017.doc]

| **Number of pathways with at least one cohort association to survival** | **Number of pathways with validation across two or more cohorts** | **Percent validation** |
| --- | --- | --- |
| **19** | **3** | **15.8%** |
| **Number of pathways with at least one cohort association to pathology** | **Number of pathways with validation across two or more cohorts** | **Percent validation** |
| **19** | **8** | **42.1%** |
| **Number of pathways with at least one cohort association to acinar subtype** | **Number of pathways with validation across two or more cohorts** | **Percent validation** |
| **14** | **3** | **21.4%** |
| **Number of pathways with at least one cohort association to papillary subtype** | **Number of pathways with validation across two or more cohorts** | **Percent validation** |
| **14** | **2** | **14.3%** |
| **Number of pathways with at least one cohort association to CIS subtype** | **Number of pathways with validation across two or more cohorts** | **Percent validation** |
| **5** | **2** | **40.0%** |
| **Number of pathways with at least one cohort association to solid subtype** | **Number of pathways with validation across two or more cohorts** | **Percent validation** |
| **11** | **3** | **27.3%** |
