# Supplementary material for: Clinically Relevant Characterization of Lung Adenocarcinoma Subtypes Based on Cellular Pathways: An International Validation Study
Source: PLoS One. 2010 Jul 22;5(7):e11712. doi: 10.1371/journal.pone.0011712 (PMC2908611; doi:10.1371/journal.pone.0011712)
Supplement: Table S10 — Additional United States gene enrichment data. (0.06 MB DOC) [file pone.0011712.s018.doc]

| **Pathway Name** | **Cluster 1 (+) Highly Ranked** | **Cluster 2 (+) Highly Ranked** | **Cluster 3 (+) Highly Ranked** | **Cluster 1 (-) Highly Ranked** | **Cluster 2 (-) Highly Ranked** | **Cluster 3 (-) Highly Ranked** |
| --- | --- | --- | --- | --- | --- | --- |
| **Cell Cycle (+)** | 0.9922743 | 0.9833811 | 1.92314e-27 | 2.07076e-23 | 0.7558203 | 0.9915762 |
| **ESC** | 0.941322 | 0.9999972 | 2.84527e-56 | 4.994906e-26 | 0.07083931 | 0.9994593 |
| **B-cell** | 1 | 0.008650041 | 1 | 1 | 1 | 0.5712174 |
| **T-cell** | 1 | 3.30449e-05 | 0.8400208 | 0.001916397 | 1 | 0.5160874 |
| **Antigen** | 1 | 0.000860358 | 1 | 1 | 1 | 0.02847337 |
| **AKT/PI3K** | 0.2636509 | 0.02172720 | 1 | 0.932536 | 1 | 0.00060895 |
| **IGF-1** | 1 | 0.02680033 | 0.5791623 | 0.04346713 | 1 | 0.785968 |
| **Chemokine** | 0.9732005 | 1.62312e-09 | 0.9780996 | 4.863784e-06 | 1 | 0.5113133 |
| **NFKB** | 0.9374347 | 0.001468725 | 0.8517913 | 0.932536 | 1 | 0.077429 |
| **Notch** | 1 | 0.01789729 | 1 | 0.7789404 | 1 | 1 |
| **JAKSTAT** | 0.7632271 | 0.0568906 | 1 | 0.1403379 | 1 | 0.1265260 |
| **Complement** | 0.0154033 | 0.2546943 | 1 | 1 | 1 | 9.79766e-05 |
| **mTOR** | 1 | 1 | 0.3030140 | 0.05924532 | 1 | 1 |
| **Cell Cycle (-)** | 0.704456 | 0.7471578 | 0.5681392 | 0.6944934 | 1 | 0.5222179 |
| **Angiogenesis** | 0.8782728 | 0.1766968 | 0.7655869 | 0.306195 | 1 | 0.5769602 |
| **IL-stimulatory** | 1 | 0.6716629 | 1 | 0.5920819 | 1 | 0.8136097 |
| **IL-suppressive** | 1 | 0.4646718 | 1 | 0.4166046 | 1 | 1 |
| **Interferon** | 1 | 1 | 1 | 1 | 1 | 1 |
| **EGFR** | 0.2030324 | 0.8466796 | 0.3030140 | 0.1923835 | 1 | 0.9183907 |
| **PDGF** | 0.4856079 | 0.5275741 | 1 | 1 | 1 | 0.2324141 |
| **Hypoxia** | 0.6311072 | 1 | 1 | 0.6209644 | 1 | 0.1489842 |
| **PTEN** | 0.830225 | 0.288237 | 1 | 0.8218358 | 1 | 0.4558766 |
| **Pro-apoptosis** | 0.9924096 | 0.02873582 | 0.9653366 | 1 | 0.2537517 | 0.2270273 |
| **Anti-apoptosis** | 0.9769567 | 0.3696088 | 0.9255146 | 0.974468 | 0.03860831 | 0.9122308 |
| **TGFB** | 1 | 0.576285 | 1 | 1 | 1 | 0.930952 |
| **Hedgehog** | 0.7632271 | 0.4621377 | 0.6293059 | 0.7537652 | 1 | 0.6164515 |
| **Wnt** | 0.3511136 | 0.9191047 | 0.8614431 | 0.9563554 | 0.2702068 | 0.2694602 |
