# Supplementary material for: Clinically Relevant Characterization of Lung Adenocarcinoma Subtypes Based on Cellular Pathways: An International Validation Study
Source: PLoS One. 2010 Jul 22;5(7):e11712. doi: 10.1371/journal.pone.0011712 (PMC2908611; doi:10.1371/journal.pone.0011712)
Supplement: Table S11 — Additional United States pathology-pathway data - CIS (+/−). (0.05 MB DOC) [file pone.0011712.s019.doc]

**CIS** component (+/-):

| **Pathway Name** | **Coefficient** | **P-value** |
| --- | --- | --- |
| **Intercept** | -1.2263 | <0.0001 |
| **Cell Cycle (+)** | -0.8639 | 0.00000245 |
| **ESC** | NA | NA |
| **B-cell** | NA | NA |
| **T-cell** | NA | NA |
| **Antigen** | 0.5167 | 0.01265 |
| **AKT/PI3K** | NA | NA |
| **IGF-1** | -0.4575 | 0.01709 |
| **Chemokine** | NA | NA |
| **NF-κB** | NA | NA |
| **Notch** | NA | NA |
| **JAK/STAT** | NA | NA |
| **Complement** | NA | NA |
| **mTOR** | NA | NA |
| **Cell Cycle (-)** | NA | NA |
| **Angiogenesis** | NA | NA |
| **IL-stimulatory** | -0.2977 | 0.12279 |
| **IL-suppressive** | -0.3229 | 0.05527 |
| **Interferon** | -0.2443 | 0.11184 |
| **EGFR** | 0.328 | 0.03777 |
| **PDGF** | NA | NA |
| **Hypoxia** | NA | NA |
| **PTEN** | NA | NA |
| **Pro-apoptosis** | NA | NA |
| **Anti-apoptosis** | 0.3353 | 0.12311 |
| **TGF-β** | NA | NA |
| **Hedgehog** | NA | NA |
| **Wnt** | 0.5548 | 0.00101 |
