# Supplementary material for: Clinically Relevant Characterization of Lung Adenocarcinoma Subtypes Based on Cellular Pathways: An International Validation Study
Source: PLoS One. 2010 Jul 22;5(7):e11712. doi: 10.1371/journal.pone.0011712 (PMC2908611; doi:10.1371/journal.pone.0011712)
Supplement: Table S13 — Additional United States pathology-pathway data - papillary (+/−). (0.04 MB DOC) [file pone.0011712.s021.doc]

Papillary component (+/-):

| **Pathway Name** | **Coefficient** | **P-value** |
| --- | --- | --- |
| **Intercept** | 1.4659 | <0.0001 |
| **Cell Cycle (+)** | -0.894 | 0.00000474 |
| **ESC** | NA | NA |
| **B-cell** | NA | NA |
| **T-cell** | NA | NA |
| **Antigen** | NA | NA |
| **AKT/PI3K** | NA | NA |
| **IGF-1** | NA | NA |
| **Chemokine** | NA | NA |
| **NF-κB** | NA | NA |
| **Notch** | NA | NA |
| **JAK/STAT** | NA | NA |
| **Complement** | 0.278 | 0.12708 |
| **mTOR** | NA | NA |
| **Cell Cycle (-)** | NA | NA |
| **Angiogenesis** | -0.3289 | 0.07119 |
| **IL-stimulatory** | -0.4321 | 0.01854 |
| **IL-suppressive** | NA | NA |
| **Interferon** | NA | NA |
| **EGFR** | 0.4619 | 0.00553 |
| **PDGF** | 0.2782 | 0.10542 |
| **Hypoxia** | NA | NA |
| **PTEN** | NA | NA |
| **Pro-apoptosis** | NA | NA |
| **Anti-apoptosis** | NA | NA |
| **TGF-β** | NA | NA |
| **Hedgehog** | 0.3219 | 0.04909 |
| **Wnt** | NA | NA |
