# Supplementary material for: Clinically Relevant Characterization of Lung Adenocarcinoma Subtypes Based on Cellular Pathways: An International Validation Study
Source: PLoS One. 2010 Jul 22;5(7):e11712. doi: 10.1371/journal.pone.0011712 (PMC2908611; doi:10.1371/journal.pone.0011712)
Supplement: Table S15 — United States tests for proportionality of hazards. (0.04 MB DOC) [file pone.0011712.s023.doc]

Tests for proportionality of hazards

| **Variable** | **Rho** | **Chi-Square** | **p-value** |
| --- | --- | --- | --- |
| **Stage 2** | -0.1043 | 1.8856 | 0.1697 |
| **Stage 3** | -0.065 | 0.7251 | 0.39447 |
| **Cell Cycle (+)** | -0.1221 | 2.3647 | 0.12411 |
| **Notch** | -0.1805 | 6.7553 | 0.00935 |
| **Hedgehog** | -0.0331 | 0.1984 | 0.65603 |
| **B-cell** | 0.0605 | 0.5974 | 0.43956 |
| **Hypoxia** | 0.1895 | 6.3827 | 0.01152 |
| **EGFR** | 0.0157 | 0.0522 | 0.81934 |
| **IL-suppressive** | -0.0973 | 2.2487 | 0.13373 |
| **Cell Cycle (-)** | 0.0284 | 0.1804 | 0.67099 |
| **Age** | -0.0938 | 1.6068 | 0.20494 |
| **Stage 2 : Notch** | 0.0804 | 1.381 | 0.23994 |
| **Stage 3 : Notch** | 0.1681 | 4.6637 | 0.03081 |
| **Stage 2 : B-cell** | 0.0304 | 0.2358 | 0.62729 |
| **Stage 3 : B-Cell** | -0.0532 | 0.5491 | 0.45868 |
| **Stage 2 : Hypoxia** | -0.1933 | 6.2772 | 0.01223 |
| **Stage 3 : Hypoxia** | -0.0632 | 0.9882 | 0.32017 |
| **Stage 2 : IL-suppressive** | -0.0251 | 0.1734 | 0.67713 |
| **Stage 3 : IL-suppressive** | 0.0996 | 1.9217 | 0.16567 |
| **GLOBAL** | NA | 25.3064 | 0.1507 |
