# Supplementary material for: Clinically Relevant Characterization of Lung Adenocarcinoma Subtypes Based on Cellular Pathways: An International Validation Study
Source: PLoS One. 2010 Jul 22;5(7):e11712. doi: 10.1371/journal.pone.0011712 (PMC2908611; doi:10.1371/journal.pone.0011712)
Supplement: Table S16 — United States multivariate Pathology Survival using Percent Component (0.03 MB DOC) [file pone.0011712.s024.doc]

Multi-variate Pathology Survival using Percent Component

| **Pathological Subtype** | **Coefficient** | **P-value** |
| --- | --- | --- |
| **Acinar-ness** | 1.00 | 0.71 |
| **Solid-ness** | 1.01 | 0.14 |
| **Papillary-ness** | 1.00 | 0.96 |
| **BAC-ness** | 0.99 | 0.22 |
